# Network Pharmacology Analysis and Experimental Verification Strategies Reveal the Action Mechanism of Danshen Decoction in Treating Ischemic Cardiomyopathy

**DOI:** 10.1155/2022/7578055

**Published:** 2022-05-02

**Authors:** Mengnan Liu, Gang Yuan, Gang Luo, Xin Guo, Mingtai Chen, Huayi Yang, Fan He, Tingfu Yang, Xinyue Zhang, Qibiao Wu, Hua Zhou, Sijin Yang

**Affiliations:** ^1^State Key Laboratory of Quality Research in Chinese Medicine, Macau Institute for Applied Research in Medicine and Health, Macau University of Science and Technology, Macau, China; ^2^National Traditional Chinese Medicine Clinical Research Base and Department of Cardiovascular Medicine, The Affiliated Traditional Chinese Medicine Hospital of Southwest Medical University, Luzhou, China; ^3^Institute of Integrated Chinese and Western Medicine, Southwest Medical University, Luzhou, China; ^4^Department of Cardiovascular Disease, Shenzhen Traditional Chinese Medicine Hospital, Shenzhen, China; ^5^Guangdong Provincial Hospital of Chinese Medicine, Guangdong Provincial Academy of Chinese Medical Sciences, State Key Laboratory of Dampness Syndrome of Chinese Medicine, Second Affiliated Hospital of Guangzhou University of Chinese Medicine, Guangdong-Hong Kong-Macau Joint Lab on Chinese Medicine and Immune Disease Research, Guangzhou, China; ^6^School of Integrated Traditional Chinese and Western Medicine, Southwest Medical University, Luzhou, Sichuan, China; ^7^Guangdong-Hong Kong-Macao Joint Laboratory for Contaminants Exposure and Health, Guangzhou, China

## Abstract

**Background:**

Danshen Decoction comprises *Salvia miltiorrhiza*, *Santalum album*, and *Amomum villosum*. It can promote blood circulation and remove blood stasis, and is commonly used in the treatment of gastric and duodenal ulcers, coronary heart disease, angina pectoris, etc. This research is based on network pharmacology and is experimentally verified to explore the potential mechanism of Danshen Decoction in the treatment of ischemic cardiomyopathy (ICM).

**Methods:**

The effective components and targets of Danshen Decoction were firstly extracted from Traditional Chinese Medicine Systems Pharmacology (TCMSP) Database and Analysis Platform, the drug-component-target-disease network was then constructed, the protein-protein interaction (PPI) network was constructed, the Gene Ontology (GO) enrichment analysis was carried out, and the Kyoto Encyclopedia of Genes and Genomes (KEGG) pathway was analyzed in order to find the potential active components and therapeutic mechanisms. Finally, the *in vitro* hypoxia/reoxygenation model in H9c2 cells was established to verify the predicted active components and therapeutic mechanisms.

**Results:**

The results showed that Danshen Decoction has 67 potential active components and 109 therapeutic targets in treating ICM. These targets were rich in a variety of gene functions and different signaling pathways; the main gene targets include TP53, c-Jun, and Akt1. Go enrichment analysis showed that response to drug, membrane raft, and G protein-coupled amine receiver activity rank first in each process, and the main signaling pathways include PI3K-Akt signaling pathway. Through molecular docking and experimental verification of the major active components and core therapeutic targets, the active components of Danshen Decoction demonstrated an ability to reduce the cell damage caused by hypoxia/reoxygenation in H9c2 cells by regulating the core therapeutic target including Akt1, c-Jun, and TP53.

**Conclusion:**

Danshen Decoction has the effect of treating ICM in multiple ways, which is consistent with the results of network pharmacology. This laid a foundation for further study in exploring the active principles and pharmacological mechanism of Danshen Decoction.

## 1. Introduction

Ischemic cardiomyopathy (ICM) is diffuse myocardial fibrosis caused by long-term myocardial ischemia. It belongs to the advanced stage of coronary heart disease (CHD) [1]. Its pathophysiological basis is that coronary atherosclerosis causes myocardial ischemia and hypoxia, as well as myocardial cell reduction, necrosis, myocardial fibrosis, and myocardial scar formation; it produces clinical syndromes similar to primary dilated cardiomyopathy, often manifested as angina pectoris, heart failure, and arrhythmia [2]. It was reported that in North America and Europe, ischemic heart disease is the main cause of more than 50% of heart failure (HF) patients, and HF affects more than 26 million people worldwide [1, 3]. ICM is mainly treated with cardiotonic, diuretic, lipid-lowering, symptomatic treatment of heart failure combined with anti-atherosclerosis and surgery, if necessary. Patients are often hospitalized repeatedly due to heart failure or arrhythmia, and the prognosis is poor [4].

Traditional Chinese medicine (TCM) has unique advantages in the treatment of myocardial ischemia. Danshen Decoction, composed of *Salvia miltiorrhiza* Bunge, root, and rhizome (Salviae Miltiorrhizae Radix et Rhizoma) (Danshen), *Santalum album* L., lignum (Santali Albi Lignum) (Tanxiang) and *Amomum villosum* Lour, fruit (Amomi Fructus) (Sharen), is commonly used in TCM for the treatment of “different pains in the heart and abdomen” [5]. Danshen Decoction was first recorded in the Chinese Qing Dynasty medical book “*Shi Fang Ge Kuo*.” The ratio of these three herbs is 10 : 1 : 1, each dose of medicine is 44.64 g and decocted with water [6]. It can promote blood circulation and remove blood stasis, promote *qi* and relieve pain, and is currently often used in the treatment of gastric and duodenal ulcers, coronary heart disease, angina pectoris, etc. Clinical and basic studies have verified that it can inhibit blood blot, and promote fibrinolysis, anti-thrombosis, and anti-atherosclerosis [5, 7, 8]. However, there are many effective ingredients in TCM decoctions; the curative effect has been clear, and the understanding of the ingredients that exert pharmacological effects is still quite limited. Although there have been some reports on the study of Danshen Decoction or Salvia miltiorrhiza in the treatment of diabetic cardiomyopathy, angina pectoris, and CHD [5, 9], we still have the value of perfecting the research on the related mechanisms of Danshen Decoction in the treatment of ICM.

**Figure 1:**
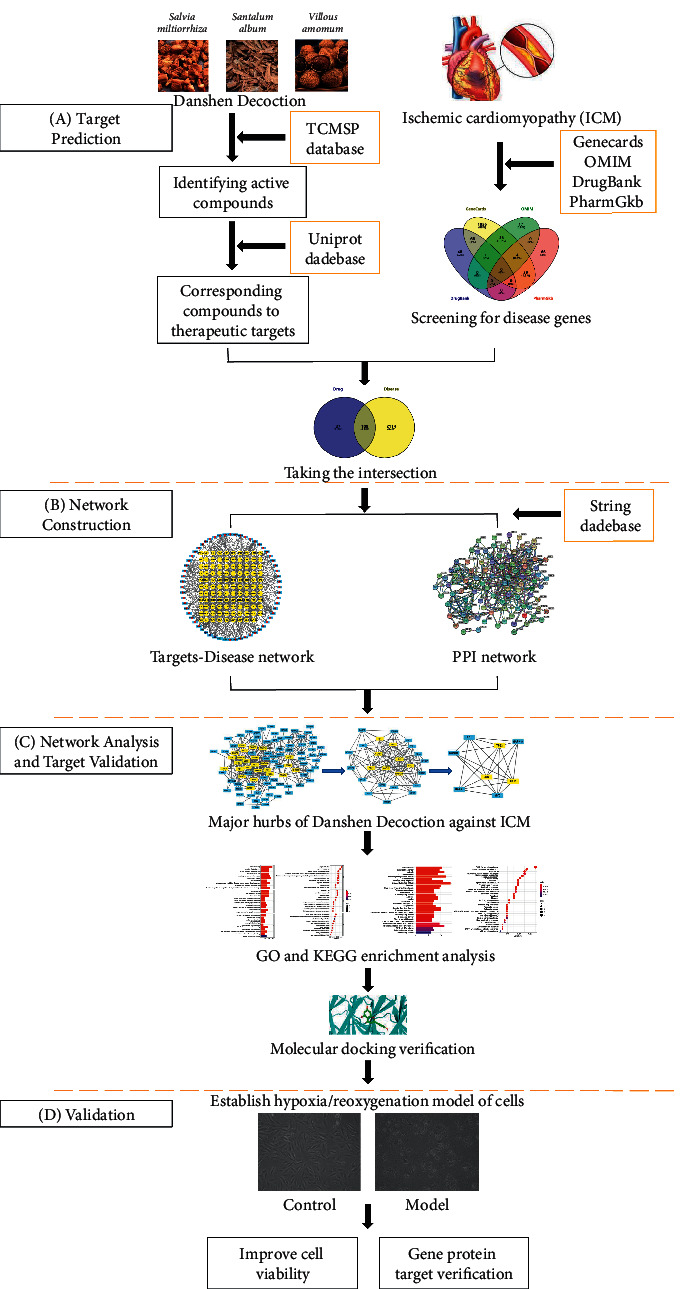
Workflow for dissecting the mechanisms of Danshen Decoction in the treatment of ischemic cardiomyopathy. The first step was to screen the target, the second step was to establish the relationship between the compound and the target, the third step was core gene screening and molecular docking, and finally *in vitro* experiments were carried out.

Network pharmacology is the use of computers, high-throughput omics data analysis, and other technologies to predict the pharmacological mechanism of Chinese medicine, which provides a new way for the study of action mechanism of Chinese medicine prescriptions, and the steps of our research also refer to the requirements of Network Pharmacology Evaluation Method Guidance [10–12]. This study uses a large database to screen the effective bioactive components and targets of Danshen Decoction, analyzes its key targets and signal pathways in the treatment of ICM, verifies it with molecular docking and *in vitro* experiments for further research on the Chinese medicine decoction, and provides guidance for disease treatment. The detailed workflow of this research is shown in Figure 1.

## 2. Methods

### 2.1. Network Pharmacology Study

#### 2.1.1. Chemical Constituents Screening

All the effective ingredients of Chinese herbal medicine contained in Danshen Decoction, including *Salvia miltiorrhiza*, *Santalum album*, and *Amomum villosum*, were retrieved through the Traditional Chinese Medicine Systems Pharmacology Database and Analysis Platform (TCMSP, https://tcmspw.com/index.php) [13, 14]; and then the candidate active compounds were screened by oral bioavailability (OB) ≥ 30% and drug similarity (DL) ≥ 0.18 as conditions, where OB refers to the relative amount and rate of absorption of the drug into the blood circulation by the body after oral administration and DL reflects the similarity between the specific functional gene in the compound and the known drug, and both are of great significance for the evaluation of the activity of the chemical components of TCM [15].

#### 2.1.2. Genes Related to ICM Retrieval

The keyword “ischemic cardiomyopathy” was entered into Genecards database (https://www.genecards.org/), OMIM database (https://omim.org/), and DrugBank database (https://www.drugbank.ca/) to collect disease-related genes with PharmGkb database (https://www.pharmgkb.org/), and all collected genes were combined and deduplicated.

#### 2.1.3. Danshen Decoction-ICM Co-Action Targets Screening and Regulatory Network Visualization

The Uniprot database (https://www.uniprot.org/) was used to search for the human gene name corresponding to the therapeutic targets of the compounds contained in the Danshen Decoction, which were then compared with the protein target gene of ICM screened in the previous step. Then, the common chemical components and common protein target genes of Danshen Decoction-ICM were introduced into Cytoscape 3.8.2 software for visualization. The targets were presented in the form of a network diagram, in which compounds and targets were represented by nodes, and the interaction relationship between nodes were represented by edges to screen core compounds. The STRING data platform (https://string-db.org/) was used to construct the protein-protein interaction (PPI) network of the target protein of Danshen Decoction in the treatment of ICM [10]. The obtained intersection targets were imported into the STRING database, the species was set to “Same Homo sapiens,” the minimum interaction threshold was set to “0.9 medium confidence,” and “hide disconnected nodes” was set, and the resulting network was saved in TSV format for future use [16]. The TSV file was imported into Cytoscape 3.8.2 software, CytoNCA plugin was used and select “Betweenness,” “Closeness,” “Degree,” “Eigenvector,” “Local Average Connectivity-Based Method,” and “Network” as the scoring basis. The larger these values, the more important the position of the node in the network [17]. After obtaining the scoring file, all genes greater than the median value were selected to obtain the core network, and the screening was repeated 3 times to obtain the core gene.

#### 2.1.4. Gene Ontology and Kyoto Encyclopedia of Genes and Genomes Enrichment Analysis

The Bioconductor database (https://www.bioconductor.org/) was used to query the gene IDs of common target genes for drugs-diseases. The R language was used to install the relevant installation package of the Bioconductor platform, *P* value = 0.05, *q* value = 0.05 were set for Gene Ontology (GO) enrichment analysis and Kyoto Encyclopedia of Genes and Genomes (KEGG) enrichment analysis, respectively, and the relevant pathway diagrams have been queried [18].

#### 2.1.5. Molecular Docking Verification

The obtained core gene was used as a protein receptor. The 2D structure of the relevant small molecule ligand was searched in the PubChem database (https://pubchem.ncbi.nlm.nih.gov/), and the ChemOffice software was used to convert it into a 3D structure; protein receptors were searched from PDB database (http://www.rcsb.org/); the Auto Dock Vina docking model was used. The lower the binding energy of the ligand and the receptor, the more stable the binding between the two. The binding energy ≤−5.0 kcal/mol^−1^ is usually used as the standard to predict the binding between the core active ingredient and the core target [19].

### 2.2. Experimental Verification

#### 2.2.1. Reagents Used in Validation

Tanshinone IIA, luteolin, and *β*-sitosterol were purchased from Chengdu Must Bio-Technology Co., Ltd. (Chengdu, China), with a purity of >98%, and the catalog numbers were A0057, A0108, and A0197, respectively. Propranolol hydrochloride was purchased from Shanghai Macklin Biochemical Co., Ltd. (Shanghai, China), with a purity of >98%, and the catalog number was P854704. TP53, c-Jun, p-c-Jun (Ser73), Akt1, and p-Akt1 (Thr308) antibodies were purchased from Affinity Biosciences Co., Ltd. (USA), the catalog numbers were DF7238, AF6090, AF3095, AF4718, and AF0832, respectively. Lactate dehydrogenase (LDH) and Malondialdehyde (MDA) kits were purchased from Beijing Solarbio Science & Technology Co., Ltd. (Beijing, China), and the catalog numbers were BC0685 and CA1410, respectively.

#### 2.2.2. Cells Culture

H9c2 cells were purchased from ATCC (Rockville, MD, United States) and cultivated under conventional conditions (37°C, 95% air/5% CO_2_) using high-sugar DMEM medium with 100 *μ*g/mL streptomycin, 100 U/mL penicillin, and 10% fatal bovine serum (FBS). The cells were passaged every 2–3 days according to cell status.

#### 2.2.3. Cell Models

The hypoxia chamber (Stem Cell Technology, USA) was used to establish a hypoxia/reoxygenation (H/R) model to create an *in vitro* hypoxic environment. The method refers to our previously published literature [20, 21]. 1 × 10^5^ cells were seeded in a six-well plate and cultured in a normal culture environment (95% air/5% CO_2_) until the cell confluence reached 80%, and then washed twice with KRB buffer (composition in mM: NaCl 115, KCl 4.7, CaCl 2.5, KH_2_PO 4.2, MgSO_4_ 1.2, NaHCO_3_ 24, HEPES 10, pH 7.4), pre-equilibrate with N_2_ overnight at 4°C. Then, 100 *μ*L KRB and 0.01% (w/v) bovine serum albumin (BSA) were added to each well and the cells were placed in the hypoxia chamber and replaced the gas with N_2_ at a flow rate of 30 mL/min for 5 min, then all the connectors of the hypoxia chamber were closed, and the chamber was moved into the 37°C incubator for 2 h. After that, the KRB buffer in the well was replaced with fresh complete medium and incubated in a normal culture environment for 1 h. Dosing intervention should be started 12 h before modeling and continue until the end of modeling.

#### 2.2.4. Cell Viability

The MTT method was used to detect the effects of the selected compounds on the proliferation of H9c2 cells. H9c2 cells in logarithmic growth phase were seeded in 96-well plates at a concentration of 5 × 10^3^ cells. After 24 h, the medium was changed and standards were added with concentrations of 1, 10, and 100 *μ*mol/L and incubated for 12 h, repeated 5 times for each group. After incubating for modeling, 20 *μ*L of MTT solution was added and incubated at 37°C for 4 h. The supernatant was aspirated and 150 *μ*L SDS hydrochloric acid was added to dissolve the crystals; microplate reader was used to detect the OD value at a wavelength of 490 nm. Cell proliferation rate = average value of experimental group/average value of control group × 100%. More accurate effective concentration was further explored based on the above experimental results. Optical microscope was used to observe cell morphology observation.

#### 2.2.5. Investigation of Predicted Compounds in Protecting from H/R Injury

H9c2 cells were processed according to the method in the section “Cell models.” They were divided into control group (cultivating under normal conditions), H/R model group, H/R + compound group (adding different compounds to incubate for 12 h during the establishment of H/R model), and H/R + propranolol group (incubating with positive control drug for 12 h during the establishment of H/R model). The process was repeated three times for each group, the supernatant was collected, and the detection of LDH and MDA were performed in accordance with the kit instructions.

#### 2.2.6. Western Blot

Protein extraction was completed on ice after modeling. The concentration of the protein sample was determined by the Bradford method, and the protein loading buffer was added in proportion to the denaturation in a metal bath at 100°C for 10 min. The proteins were separated by SDS-bisacrylamide gel electrophoresis and then transferred onto the PVDF membrane. The TBST solution containing 5% BSA was used for blocking, then TP53, c-Jun, p-c-Jun, Akt1, and p-Akt1 (1 : 1000) antibodies were added and incubated at 4°C overnight. After washing the next day, the secondary antibody (1 : 5000) was added and incubated at room temperature for 2 h. After washing again, the Amersham ImageQuant 800 system (Global Life Sciences Solutions USA LLC, Marlborough, MA, USA) was used to acquire images and analyze the gray values of the bands.

### 2.3. PCR

The Trizol method was used to extract total RNA from cells. After reverse transcription and amplification, fluorescent quantitative PCR instrument (Roche, LightCycler 480II) was used for routine melting curve analysis to determine the Ct value, and GAPDH was used as an internal reference for control. The primers needed for the experiment were synthesized by Shanghai Shenggong Company. The primer sequences used in the present study were listed as follows: Akt1-q-F: CACAGGTCGCTACTATGCCATGAAG, Akt1-q-R: GCAGGACACGGTTCTCAGTAAGC, TP53-q-F: GTACCGTATGAGCCACCTGAG,

TP53-q-R: TCCAGCGTGATGATGGTAAG, c-Jun-q-F: GTCCTCCATAAATGCCTGTTCC, c-Jun-q-R: GATGCAACCCACTGACCAGAT, GAPDH-q-F: GGACCTCATGGCCTACATGG, GAPDH-q-R:TAGGGCCT CTC TTGCTCAGT.

### 2.4. Statistical Analysis

GraphPad Prism (Ver 8.2.1, GraphPad Software, San Diego, CA, USA) was used to perform statistical analysis on the results. The data were expressed as mean ± standard deviation. One-way ANOVA analysis of variance was used in statistics. The LSD-t test was used for pairwise comparisons between groups, and *P* < 0.05 was considered as statistically significant.

## 3. Results

### 3.1. Identification of Putative Ingredient Targets

The compounds contained in *Salvia miltiorrhiza*, *Santalum album*, and *Amomum villosum* were 202, 70, and 165, respectively. According to the characteristics of the OB and DL of the compounds, 58, 3, and 10 compounds were screened out; these compounds correspond to 932, 78, and 100 targets, respectively; the full name of targets were converted to Gene Symbol through analysis and conversion in the Uniprot database, the compounds contained in *Salvia miltiorrhiza*, *Santalum album*, and *Amomum villosum* correspond to 824, 89, and 63 genes, respectively. The active compounds of each component herb involved in this study are shown in Table 1, and the detailed information of the hypothetical components of the target genes are shown in Table S1.

#### 3.1.1. Identification of Disease-Related Genes

There are 2092, 8, 99, and 115 ICM-related gene targets found in the four databases of GeneCards, OMIM, PharmGkb, and DrugBank, respectively, and the targets obtained from the four databases were combined and deduplicated to determine them as targets related to ICM, and a total of 2223 ICM targets were obtained. The genes of these databases were integrated in Table S2. The Venn diagram of overlapping genes is shown in Supplementary Figure S1.

#### 3.1.2. Network Visualization

**Figure 2:**
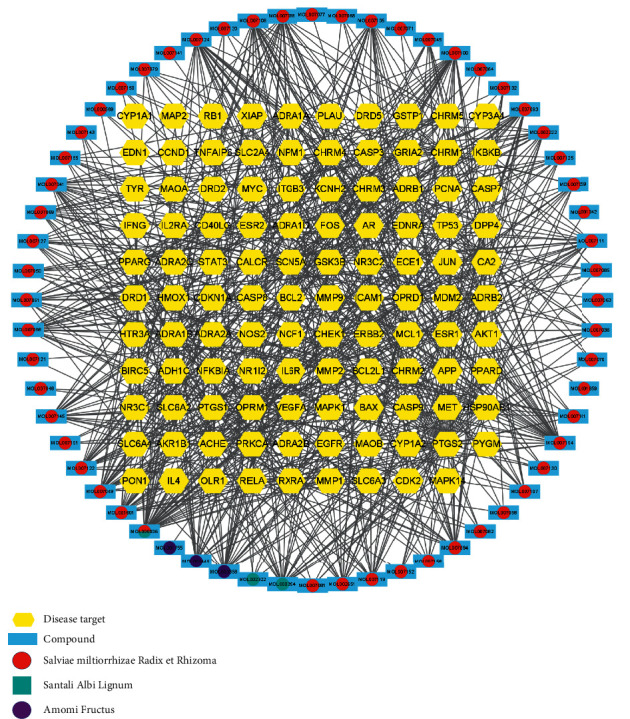
Ingredient–target network. Yellow represents the disease target, blue represents the compound, red represents *Salvia miltiorrhiza*, cyan represents *Santalum album*, purple represents *Amomum villosum*, and the connection of lines represents their correlation.

The Venny 2.1 online tool was used to find the interconnection of 136 therapeutic targets contained in Danshen Decoction and 2223 ICM-related targets, and a total of 109 overlapping genes were obtained; the Venn diagram is shown in Figure S2. The active compounds of Danshen Decoction and their corresponding disease-drug overlapping targets were introduced into Cytoscape 3.8.2 to form a network diagram of Danshen Decoction-compounds-ICM targets as shown in Figure 2. The network consists of 169 nodes and 737 edges. The nodes represent the active compounds and target proteins that meet the relevant targets, and the edges represent the interaction between the active compounds and the target proteins. Among these targets, they were ranked by the number of interconnections as shown in Table S3. Luteolin involves 43 targets, tanshinone IIA involves 35 targets, and *β*-sitosterol involves 24 targets. The top 5 of these compounds were used as candidate compounds to verify their relationship with the targets.

**Figure 3:**
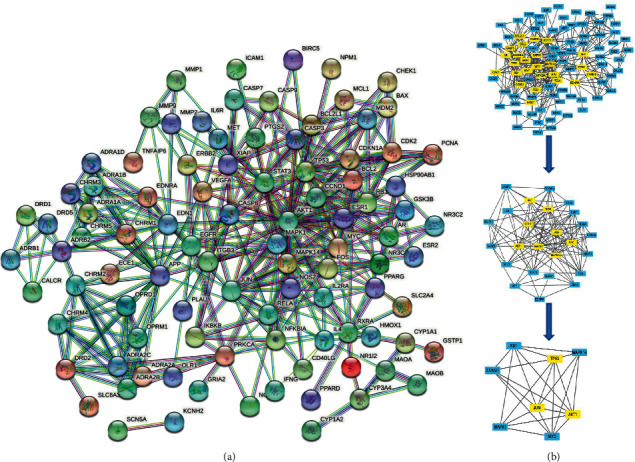
Overlapped genes interaction. (a) PPI network showing interactions between the involved genes. The more connections between each gene indicates that they are more closely related. (b) Core gene target screening. Yellow represents the selected genes.

Subsequently, 109 drug-disease overlapping genes were submitted to the STRING database, and 96 PPI network target genes were obtained. The network relationship consisting of 96 nodes and 388 edges is shown in Figure 3(a). The edges represent the degree of correlation between proteins. The TSV file was downloaded in STRING and imported into Cytoscape 3.8.2 software, CytoNCA was used to perform PPI network analysis on the TSV file to obtain the core gene network, then 3 core genes were finally screened out. The screening process is shown in Figure 3(b), and the scoring data are shown in Table S4. These genes include TP53, Akt1, and Jun. In addition, some nodes with high degree such as EGFR, BCL2, VEGFA, FOS, CASP3, and CASP8 were also considered to be important targets for the treatment of ICM.

#### 3.1.3. GO and KEGG Enrichment Analyses

**Figure 4:**
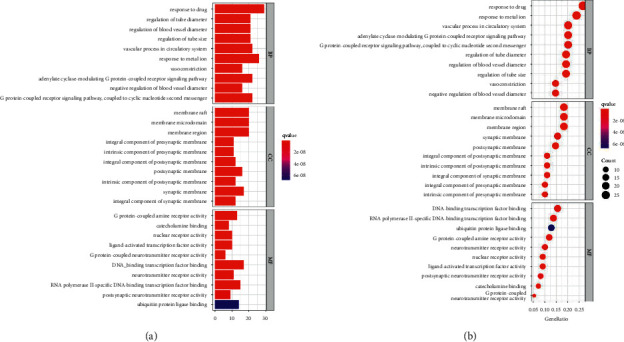
GO enrichment analyses. (a) Box plot of GO enrichment. (b) Dot plot of GO enrichment. The color changes from red to blue, indicating that the *P* value of the path changes from large to small; and the larger the surface area, the greater the enrichment degree.

To further clarify the possible effects of the 109 candidate targets, GO enrichment analysis and KEGG biological pathway analysis were carried out on the 109 candidate targets of Danshen Decoction in the treatment of ICM. There are 2005 entries in biological process (BP), 930 entries in cellular component (CC), and 664 entries in molecular function (MF). They rank first in response to drug, membrane raft, and G protein-coupled amine receiver activity. The top 10 entries in each process are shown in Figure 4. In BP, the response to drugs, the response to metal ion ranked first; the regulation of cell membranes and cell synapses are ranked first in CC; in MF, it is closely related to DNA-binding transcription factor binding.

**Figure 5:**
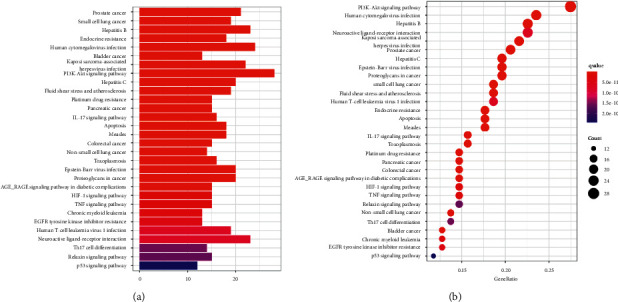
KEGG enrichment analyses. (a) Box plot of KEGG enrichment. (b) Dot plot of KEGG enrichment. The color changes from red to blue, indicating that the *P* value of the path changes from large to small; and the larger the surface area, the greater the enrichment degree.

KEGG biological pathway analyzed a total of 159 related signal pathways, the top three related signal pathways include PI3K-Akt signaling pathway, human cytomegalovirus infection, and hepatitis B, and the top 30 entries are shown in Figure 5. The results showed that the mechanism of Danshen Decoction in the treatment of ICM mainly relates to multiple tumor-related pathways. The PI3K-Akt signal pathway with the highest degree of enrichment was selected to draw a pathway diagram, as shown in Figure S3.

#### 3.1.4. Molecular Docking Verification

The TP53, Akt1, and c-Jun screened out in the PPI network were used as core receptors for molecular docking with corresponding compounds. Among them, TP53 corresponds to tanshinone IIA and luteolin; Akt1 corresponds to luteolin; c-Jun corresponds to tanshinone IIA, luteolin, and *β*-sitosterol. Next, Pymol 2.3.2 software was used for visual analysis of molecular docking. When the three compounds were docked with the corresponding targets, the analysis found that multiple active sites could form hydrogen bonds with different amino acids, and the average affinity of this docking was less than −5.0 kcal/mol^−1^. However, although tanshinone IIA and *β*-sitosterol cannot bind to JUN at the hydrogen bonding site, the potential binding of other sites were still shown in the simulated docking. Therefore, they all showed good binding activity to the target, which may be the therapeutic agent for the corresponding target, as shown in Table 2.

#### 3.1.5. Intervention Concentration Screening

**Figure 6:**
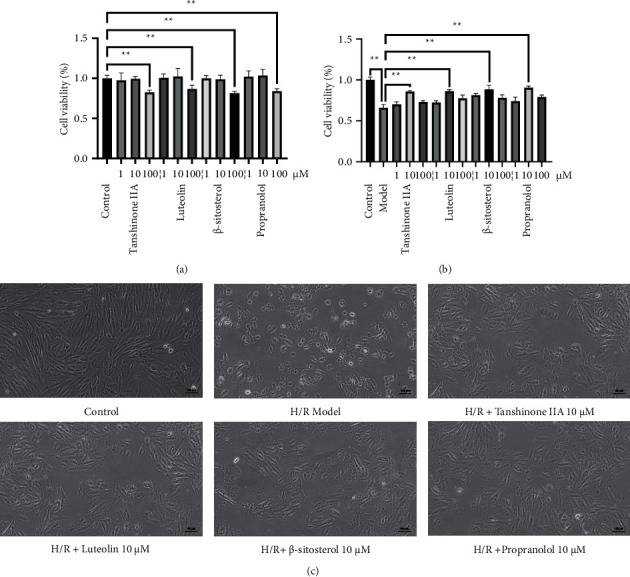
The effect of predicted compounds on cell viability. (a) Different concentrations of active compounds on cell viability. (b) Exploration of optimal dosage of active compounds on improvement rate (*n* = 5). ^∗∗^*P* < 0.01 vs. Model group. (c) Alterations of cellular morphology. The cells were placed under an inverted microscope for observation (×200).

Three potential effective compounds including tanshinone IIA, luteolin, and *β*-sitosterol (Table 2) were selected for *in vitro* experiments to verify the virtual screening, and propranolol was used as the positive control drug. The concentrations of 1, 10, and 100 *μ*M were firstly used and the results showed that when the concentration reached 100 *μ*M, the cell activity decreased significantly, so 10 *μ*M was selected for further experiment (Figure 6(a)). Then the improvement rate under different concentrations was studied to obtain the best dosage. After H/R intervention, we got similar results, the optimal dosages of tanshinone IIA, luteolin, *β*-sitosterol and propranolol were 10 *μ*M respectively, when the concentration exceeds 100 *μ*M, the relative cell viability of each test decreases to varying degrees (Figure 6(b)). In terms of cell morphology, the control group has a large number of cells, complete morphology, and monolayer cluster growth; the model group has a large number of nuclear shrinkages, the cells become rounded, and floated in the medium; after the intervention of the compounds, the cell morphology has improved to varying degrees with fewer floating cells (Figure 6(c)).

#### 3.1.6. Predicted Effective Compounds Protect H9c2 Cells from H/R Injury

**Figure 7:**
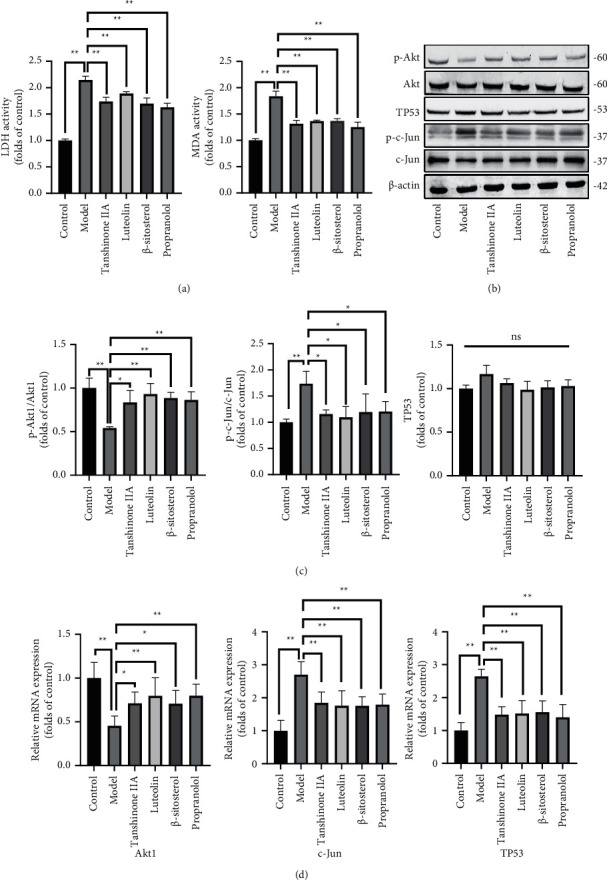
The effect of predicted compounds on improving cell hypoxia in multiple ways. (a) The expressions of LDH and MDA from different groups (*n* = 3). (b) Western blot bands. (c) The expressions of TP53, p-c-Jun, and p-Akt from different groups were detected by Western blot assay (*n* = 3). (d) The expression of Akt, c-Jun, and TP53 mRNA in different groups was detected by PCR (*n* = 6). The results were presented as mean ± SD. ^∗^*P* < 0.05, ^∗∗^*P* < 0.01 vs. Model group.

Based on the findings of this study for predicting therapeutic targets and related signal pathways, the effect of the core compounds of Danshen Decoction on the H/R model was verified. After H/R treatment, the content of LDH and MDA in the cell supernatant was reduced by these compounds (*P* < 0.05), which reflects the different degrees of protection of these compounds on cells (Figure 7(a)). Then, the indicators of TP53, c-Jun, and Akt1 were used to detect protein expression, and the immunoblotting bands were shown in Figure 7(b). Compared with the H/R group, these compounds all had a significant inhibitory effect on the expression of p-c-Jun protein (*P* < 0.05), and had a significant enhancement effect on the expression of p-Akt1 protein (*P* < 0.05); however, these compounds did not show a significant effect on TP53 compared with the model group (*P* > 0.05) (Figure 7(c)). However, at the level of gene expression, we found that these compounds significantly increased Akt and decreased c-Jun, TP53 mRNA expression (*P* > 0.05) (Figure 7(d)). In short, the active compounds of Danshen Decoction can regulate Jun and Akt1 as protein targets to improve myocardial hypoxia.

## 4. Discussion

Network pharmacology is designed to study drug treatments with unclear targets or multiple targets to predict possible therapeutic effects [15]. Danshen Decoction is a mixture of 3 kinds of herbs with multiple compounds and targets, the network pharmacology study of Danshen Decoction in the treatment of ICM conforms to the above point of view, and the compounds we studied were closed to the protective effect of the positive control drug propranolol on the H/R mode; this provides a reference for future in-depth research. TCM with Danshen as the main ingredient has been verified in some studies to have the effect of treating or preventing cardiovascular diseases. For example, Danshen injection can significantly prevent myocardial fibrosis, cardiac hypertrophy, hemodynamic deterioration, and systolic and diastolic dysfunction characterized by failed hearts [8]. Danshen-Gegen Decoction can inhibit cell apoptosis induced by H/R by inhibiting the transition of mitochondrial permeability [22]. Many studies published in Chinese journals also showed that Danshen Decoction has a definite effect on myocardial ischemia [23]. Therefore, it is not necessary for us to carry out repeated in vitro experimental verification. We focused on these active ingredients and the most closely interacting targets screened by network pharmacology in order to illustrate the material basis of Danshen Decoction against myocardial ischemia.

Three effective ingredients were screened out in this study. Luteolin is a widely distributed flavonoid compound that can be found in a variety of plants. A large number of studies have found that luteolin is a potential cardioprotective agent, and it also has many evidences of cardioprotection in epidemiology [24]. *β*-sitosterol can also be found in a variety of plants. The current research showed that the content of sitosterol in the circulating blood may have a certain relationship with the cardiovascular disease [25]. An in vitro study by Huang et al. showed that *β*-sitosterol regulates the cell cycle by promoting cellular glutathione production and protects H9c2 cells from apoptosis induced by H/R [26]. Tanshinone IIA is obviously the most specific compound among these three herbs. It is the main fat-soluble component of *Salvia miltiorrhiza*. Modern pharmacological studies have shown that tanshinone IIA has anti-inflammatory and antioxidant activities and has a wide range of treatments for cardiovascular diseases [27]. In this experimental verification, we were able to determine the role of the compound itself, which on the other hand also shows that the method of network pharmacology has a certain degree of reliability. However, it cannot be ignored that although these three compounds have been confirmed to have the effect of saving cell death in our experimental verification, the dose-response relationship cannot be well reflected due to the large drug dose gap set in our study. But there is no doubt that the therapeutic effects of these compounds have been demonstrated.

GO function analysis showed that the core targets of Danshen Decoction in the treatment of ICM were TP53, Akt1, Jun, etc. TP53 is a human tumor suppressor gene, and it has been confirmed that the expression of this gene is closely related to the suppression of tumor cells [28]. Regulating the expression of TP53 protein can promote the repair process of heart endothelial cells and help vascular remodeling to reduce cardiovascular damage [29]. In our study, the protective effect of TP53 on cardiomyocytes was not significant, but its trend was obvious. We considered that this was related to our short-term modeling method, and the protein expression did not reach the peak at this time. Akt involves in the occurrence and development of inflammation, cancer, diabetes, and cardiovascular diseases; the functions include the regulation of cell cycle and transcription [30]. Akt1 plays an important role in multiple signaling pathways such as PI3K-Akt, NF-*κ*B, and Toll-like receptors. Research has shown that the expression and activation of Akt1 are involved in the progression of myocardial fibrosis [31]; Jun can serve as an important transcription factor in the cell and widely involves in tumor regulation, smooth muscle proliferation, and apoptosis [32, 33]. According to the enrichment of targets, we also found that some other important targets, such as CASP3, play a central role in the execution of apoptosis, and can regulate cell proliferation, survival, migration, and other physiological functions to protect damaged myocardium [34]. NF-*κ*B participates in the response of cells to external stimuli, such as cytokines, radiation, heavy metals, viruses, etc., and plays a key role in the process of cellular inflammation and immune response [35]. IL-1*β*, as a downstream inflammatory factor of inflammation-related signal pathways, has been proved to have extensive regulation on the inflammatory response of blood vessels and myocardium [35, 36]. MAPK14 and MAPK1 can regulate various physiological and pathological processes and the inflammatory responses of cells through the MAPK signaling pathway [37]. VEGFA is the central factor in the regulation of neovascularization, which can accelerate the proliferation and differentiation of vascular endothelium [38]. From the verification of molecular docking, it was found that the core active ingredients predicted by Danshen Decoction have good binding activity with the core target for the treatment of ICM, which is consistent with the results of network pharmacology screening and has potential therapeutic significance. Our experiments also verified that TP53, Akt1, and Jun were involved in the repair of cardiomyocyte H/R model, which is consistent with the trend of research reported in the literature [5, 39–42]. Reactive oxygen species (ROS) are highly active molecules *in vivo*. In the human body, ROS acts on lipid to produce peroxidation reaction, the end product of oxidation is MDA, which has cytotoxicity and causes cross-linking polymerization of proteins, nucleic acids, and other life macromolecules. In clinical practice, LDH is often used as an auxiliary diagnostic index of acute myocardial infarction. The determination of three indexes was used to investigate the protective effect of core compounds on the H/R model. The experiment showed that these indexes significantly decreased compared with the model group after using the compounds intervention, indicating that the predicted core compounds to protect heart is likely the active ingredient of Danshen Decoction.

The enrichment of the KEGG pathway of core genes showed that the core targets were involved in multiple pathways related to tumors. Others include PI3K-Akt, fluid shear stress and atherosclerosis, IL-17 signaling pathway, apoptosis, HIF-1, tumor necrosis factor, EGFR, etc. These signaling pathways have been studied in depth in cardiovascular diseases [39, 41]. PI3K-Akt signaling pathway enriched most genes in this study, which involves in the regulation of cell proliferation and plays an important role in combating myocardial H/R injury and inhibiting cell apoptosis. Regarding the preliminary verification of this pathway, we have found that Akt1 is involved in the repair process of cell H/R injury, demonstrating the reliability of this network pharmacology study.

Although this study has been partially verified, the following problems still exist due to the limitations of the network pharmacology method: First, the components directly extracted from TCMSP may be inconsistent with the exact compounds absorbed by the patient; second, the compounds included in this study need to meet the OB and DL limits, and there is excessive screening; third, due to the diversity of the corresponding targets of the compound, there may be errors in the enrichment analysis of GO and KEGG; fourth, with the continuous update of the data of each database, the results of future research may be more abundant. However, there must be some errors in the current research. Such problems have been exposed in this study; for example, *β*-sitosterol in this study, the corresponding therapeutic targets were Akt1 only, and the corresponding targets of tanshinone IIA include TP53 and c-Jun. However, after experimental verification, we found that these compounds have regulatory effects on Akt1, TP53, and c-Jun, which may be bound up with the delay in updating the network pharmacology–related database. Furthermore, the interaction between multiple compounds was not considered in this study, and the most attractive part of traditional Chinese medicine itself is that many compounds play a wide range of regulatory roles, which should be paid attention to in future research. In addition, we did not detect the contents of various predicted compounds in Danshen Decoction, and the data of these substances were obtained from the database. Unfortunately, the proportion of these three compounds verified by our experiments has not been obtained through literature review, which makes it impossible for us to mix these three compounds in proper proportion to observe their improvement on cell damage. Last but not least, verification was carried out at the level of *in vitro* experiments, and verification in animal models will also be required in future studies with higher levels of evidence. In future research, we will continue to reveal the mechanism of Danshen Decoction in the treatment of related diseases through *in vitro* and *in vivo* experiments.

## 5. Conclusion

Our research provides molecular evidence for reference to prove that Danshen Decoction has the effect of treating ischemic cardiomyopathy, and the mechanism of such treatment is the participation of multiple compounds, pathways, and targets. Its core components can regulate cytokine levels and improve hypoxia from multiple aspects, so as to protect the cardiomyocytes of ICM patients. Our network pharmacology research and experimental verification have laid the foundation for further study on the pharmacological mechanism of Danshen Decoction.

## Figures and Tables

**Table 1:** Detailed information of active ingredients of Danshen Decoction.

Hurb	Mol ID	Compound	Formula	OB (%)	DL
Salvia miltiorrhiza	MOL001601	1,2,5,6-tetrahydrotanshinone	C_18_H_16_O_3_	38.75	0.36
	MOL001659	Poriferasterol	C_29_H_50_O	43.83	0.76
	MOL001942	Isoimperatorin	C_16_H_14_O_4_	45.46	0.23
	MOL002222	Sugiol	C_20_H_28_O_2_	36.11	0.28
	MOL002651	Dehydrotanshinone IIA	C_19_H_16_O_3_	43.76	0.4
	MOL000569	Digallate	C_14_H_9_O_9_^−^	40.12	0.26
	MOL007036	5,6-dihydroxy-7-isopropyl-1,1-dimethyl-2,3-dihydrophenanthren-4-one	C_19_H_22_O_3_	36.16	0.29
	MOL007041	2-isopropyl-8-methylphenanthrene-3,4-dione	C_18_H_16_O_2_	39.51	0.23
	MOL007045	3*α*-hydroxytanshinoneIIa	C_19_H_18_O_4_	33.77	0.44
	MOL007048	(E)-3-[2-(3,4-dihydroxyphenyl)-7-hydroxy-benzofuran-4-yl]acrylic acid	C_17_H_12_O_6_	40.86	0.31
	MOL007049	4-methylenemiltirone	C_18_H_18_O_2_	44.93	0.23
	MOL007050	2-(4-hydroxy-3-methoxyphenyl)-5-(3-hydroxypropyl)-7-methoxy-3-benzofurancarboxaldehyde	C_20_H_20_O_6_	48.24	0.4
	MOL007058	Formyltanshinone	C_18_H_10_O_4_	34.35	0.42
	MOL007059	3-beta-Hydroxymethyllenetanshiquinone	C_18_H_14_O_4_	62.78	0.41
	MOL007061	Methylenetanshinquinone	C_18_H_14_O_3_	46.69	0.36
	MOL007063	Przewalskin a	C_23_H_30_O_6_	73.44	0.65
	MOL007064	Przewalskin b	C_20_H_26_O_4_	32.16	0.44
	MOL007068	Przewaquinone B	C_18_H_12_O_4_	37.07	0.41
	MOL007069	Przewaquinone c	C_18_H_16_O_4_	37.11	0.4
	MOL007070	(6S,7R)-6,7-dihydroxy-1,6-dimethyl-8,9-dihydro-7H-naphtho[8,7-g]benzofuran-10,11-dione	C_18_H_16_O_5_	110.32	0.45
	MOL007071	Przewaquinone f	C_18_H_16_O_5_	62.24	0.46
	MOL007077	Sclareol	C_20_H_36_O_2_	55.74	0.21
	MOL007079	Tanshinaldehyde	C_19_H_18_O_4_	41.31	0.45
	MOL007081	Salvia miltiorrhizaol B	C_22_H_26_O_4_	40.31	0.56
	MOL007082	Salvia miltiorrhizaol A	C_21_H_20_O_4_	43.67	0.52
	MOL007085	Salvilenone	C_20_H_20_O_2_	52.47	0.38
	MOL007088	Cryptotanshinone	C_19_H_20_O_3_	57.95	0.4
	MOL007093	Dan-shexinkum d	C_21_H_20_O_4_	56.97	0.55
	MOL007094	Salvia miltiorrhizaspiroketallactone	C_20_H_20_O_5_	30.38	0.31
	MOL007098	Deoxyneocryptotanshinone	C_19_H_22_O_3_	52.34	0.29
	MOL007100	Dihydrotanshinlactone	Not found	38.88	0.32
	MOL007101	Dihydrotanshinone I	C_18_H_14_O_3_	50.43	0.36
	MOL007105	EpiSalvia miltiorrhizaspiroketallactone	C_17_H_16_O_3_	49.4	0.31
	MOL007107	C09092	C_20_H_30_O	38.68	0.25
	MOL007108	Isocryptotanshi-none	C_19_H_20_O_3_	45.04	0.39
	MOL007111	Isotanshinone II	C_18_H_12_O_3_	68.27	0.4
	MOL007115	Manool	C_20_H_34_O	36.07	0.2
	MOL007119	Miltionone I	C_19_H_20_O_4_	54.98	0.32
	MOL007120	Miltionone II	C_19_H_20_O_4_	49.92	0.44
	MOL007121	Miltipolone	C_19_H_24_O_3_	45.04	0.37
	MOL007122	Miltirone	C_19_H_22_O_2_	39.61	0.25
	MOL007124	Neocryptotanshinone II	C_19_H_22_O_3_	49.68	0.23
	MOL007125	Neocryptotanshinone	C_19_H_22_O_4_	71.03	0.32
	MOL007127	1-methyl-8,9-dihydro-7H-naphtho[5,6-g]benzofuran-6,10,11-trione	C_17_H_12_O_4_	36.56	0.37
	MOL007130	Prolithospermic acid	C_17_H_14_O_6_	38.76	0.31
	MOL007132	(2R)-3-(3,4-dihydroxyphenyl)-2-[(Z)-3-(3,4-dihydroxyphenyl)acryloyl]oxy-propionic acid	C_18_H_16_O_8_	44.95	0.35
	MOL007141	Salvianolic acid g	C_18_H_12_O_7_	39.46	0.61
	MOL007142	Salvianolic acid j	C_27_H_22_O_12_	52.49	0.72
	MOL007143	Salvilenone I	C_20_H_20_O_2_	34.72	0.23
	MOL007145	Salviolone	C_18_H_20_O_2_	64.37	0.24
	MOL007150	(6S)-6-hydroxy-1-methyl-6-methylol-8,9-dihydro-7H-naphtho[8,7-g]benzofuran-10,11-quinone	C_18_H_16_O_5_	109.38	0.46
	MOL007151	Tanshindiol B	C_18_H_12_O_4_	88.54	0.45
	MOL007152	Przewaquinone E	C_18_H_16_O_5_	45.56	0.45
	MOL007154	Tanshinone IIA	C_19_H_18_O_3_	43.38	0.4
	MOL007155	(6S)-6-(hydroxymethyl)-1,6-dimethyl-8,9-dihydro-7H-naphtho[8,7-g]benzofuran-10,11-dione	C_19_H_18_O_4_	32.43	0.45
	MOL007156	Tanshinone VI	C_18_H_16_O_4_	31.72	0.3

Santalum album, Salvia miltiorrhiza	MOL000006	Luteolin	C_15_H_10_O_6_	75.39	0.25

Santalum album	MOL000354	Isorhamnetin	C_16_H_12_O_7_	34.49	0.31
	MOL002322	Isovitexin	C_21_H_20_O_10_	42.67	0.72

Salvia miltiorrhiza, Villous amomum fruit	MOL001771	Poriferast-5-en-3beta-ol	C_29_H_50_O	49.89	0.75

Villous amomum fruit	MOL001755	24-Ethylcholest-4-en-3-one	C_29_H_48_O	42.85	0.76
	MOL001973	Sitosteryl acetate	C_31_H_52_O_2_	65.26	0.85
	MOL000358	*β*-sitosterol	C_29_H_50_O	45.64	0.75
	MOL000449	Stigmasterol	C_29_H_48_O	36.16	0.76
	MOL007514	Methyl icosa-11,14-dienoate	C_21_H_38_O_2_	49.6	0.23
	MOL007535	(5S,8S,9S,10R,13R,14S,17R)-17-[(1R,4R)-4-ethyl-1,5-dimethylhexyl]-10,13-dimethyl-2,4,5,7,8,9,11,12,14,15,16,17-dodecahydro-1H-cyclopenta[a]phenanthrene-3,6-dione	C_29_H_48_O_2_	31.29	0.79
	MOL007536	Stigmasta-5,22-dien-3-beta-yl acetate	C_31_H_50_O_2_	39.67	0.86

**Table 2:** Molecular docking verification.

Target gene	PDB ID	Compound	Formula	Molecular docking diagram	Binding site	Docking diagrams (kcal/mol)
TP53	2J21	Luteolin	C_15_H_10_O_6_	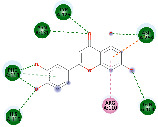	ASP A:268, ASN A:131, TYR A:126, GLN A:104, PHE A:113, HIS A:115, ARG A:110	−7.0
		Tanshinone IIA	C_19_H_18_O_3_	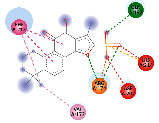	GLN A:192, ARG A:174, HIS A:214, ASP A:207, VAL A:172, PHE A:212	−7.1

AKT1	4GV1	Luteolin	C_15_H_10_O_6_	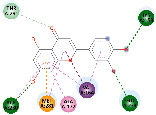	GLU-234, ALA-230, LYS-158, THR A:291, MET A:281, ALA A:177, VAL A:164	−8.1

JUN	1Jun	Luteolin	C_15_H_10_O_6_	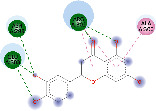	GLU A:303, ASN A:299, ARG A:302, ALA A:306	−5.1
		Tanshinone IIA	C_19_H_18_O_3_	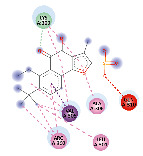	LYS A:309, GLN A:310, ALA A:306, VAL A:305, ARG:320, LEU A:301	−6.0
		*β*-sitosterol	C_29_H_50_O	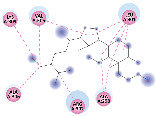	LYS A:309, VAL A:305, LEU A:301, ALA A:306, ARG A:302, ALA A:298	−5.8

## Data Availability

The datasets used or analyzed during the current study are available from the corresponding author.

## Abbreviations

Akt: Protein kinase B
BCL: B-cell lymphoma
BP: Biological process
CASP: Cysteinyl aspartate specific proteinase
CC: Cellular component
CHD: Coronary heart disease
DL: Drug similarity
EGFR: Epidermal growth factor receptor
GO: Gene ontology
KEGG: Kyoto encyclopedia of genes and genomes
HF: Heart failure
ICM: Ischemic cardiomyopathy
IL: Interleukin
Jun: Jun activation domain-binding protein
LDH: Lactate dehydrogenase
MDA: Malondialdehyde
MF: Molecular function
OB: Oral bioavailability
ROS: Reactive oxygen species
TCM: Traditional Chinese medicine
TCMSP: Traditional Chinese medicine systems pharmacology database and analysis platform
TP53: Tumor protein P53
VEGFA: Vascular endothelial growth factor.

## Supplementary Materials

Supplementary Materials Figure S1. Venn diagram showing gene targets related to ICM. Figure S2. Venn diagram showing the overlapped genes between Danshen Decoction and ICM. Figure S3. PI3K-Akt signal pathway. Table S1. Eligible and putative ingredients with targeted genes. Table S2. ICM-related genes. Table S3. Sequence of compounds linkage gene targets. Table S4. Core gene target score.
