# Correction to “Network Pharmacology Analysis and Experimental Verification Strategies Reveal the Action Mechanism of Danshen Decoction in Treating Ischemic Cardiomyopathy”

**DOI:** 10.1155/ecam/9813598

**Published:** 2025-09-23

M. Liu, G. Yuan, G. Luo, et al., “Network Pharmacology Analysis and Experimental Verification Strategies Reveal the Action Mechanism of Danshen Decoction in Treating Ischemic Cardiomyopathy,” *Evidence-Based Complementary and Alternative Medicine* 2022 (2022): 7578055, https://doi.org/10.1155/2022/7578055.

In the article, there is an error in Figure 6 where the H/R + tanshinone IIA 10 μM panel is mistakenly duplicated with the H/R + luteolin IIA 10 μM panel. The correct Figure 1 is shown below.

We apologize for this error.

## Figures and Tables

**Figure 1:**
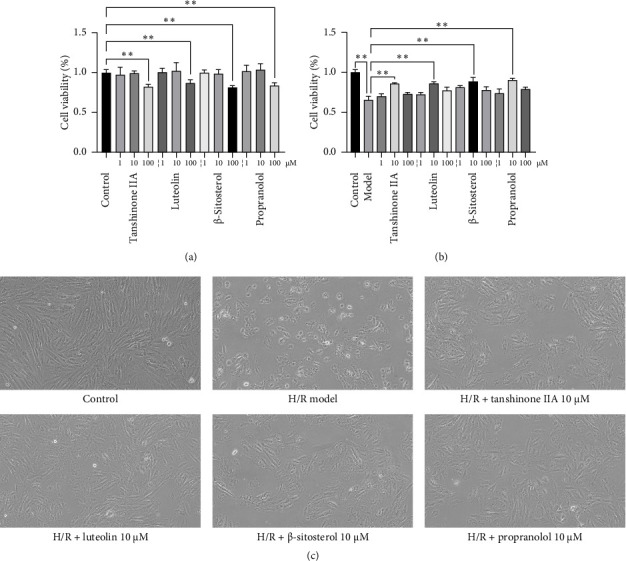
The effect of predicted compounds on cell viability. (a) Different concentrations of active compounds on cell viability. (b) Exploration of optimal dosage of active compounds on improvement rate (*n* = 5). ^∗∗^*p* < 0.01 vs. model group. (c) Alterations of cellular morphology. The cells were placed under an inverted microscope for observation (× 200).

