# Supplementary material for: Network Pharmacology Analysis and Experimental Verification Strategies Reveal the Action Mechanism of Danshen Decoction in Treating Ischemic Cardiomyopathy
Source: Evid Based Complement Alternat Med. 2022 May 2;2022:7578055. doi: 10.1155/2022/7578055 (PMC9205745; doi:10.1155/2022/7578055)
Supplement: Supplementary Materials — Figure S1. Venn diagram showing gene targets related to ICM. Figure S2. Venn diagram showing the overlapped genes between Danshen Decoction and ICM. Figure S3. PI3K-Akt signal pathway. Table S1. Eligible and putative ingredients with targeted genes. Table S2. ICM-related genes. Table S3. Sequence of compounds linkage gene targets. Table S4. Core gene target score. [file 7578055.f1.docx]

| Table S1. Eligible and putative ingredients with targeted genes | | | |  |  |  |  |  |  |
| --- | --- | --- | --- | --- | --- | --- | --- | --- | --- |
| Drug | Mol_Id | Mol_Name | Gene_Symbol |  |  |  |  |  |  |
| Salviae Miltiorrhizae Radix et Rhizoma | MOL001601 | 1,2,5,6-tetrahydrotanshinone | PTGS1 |  |  |  |  |  |  |
| Salviae Miltiorrhizae Radix et Rhizoma | MOL001601 | 1,2,5,6-tetrahydrotanshinone | CHRM3 |  |  |  |  |  |  |
| Salviae Miltiorrhizae Radix et Rhizoma | MOL001601 | 1,2,5,6-tetrahydrotanshinone | CHRM1 |  |  |  |  |  |  |
| Salviae Miltiorrhizae Radix et Rhizoma | MOL001601 | 1,2,5,6-tetrahydrotanshinone | SCN5A |  |  |  |  |  |  |
| Salviae Miltiorrhizae Radix et Rhizoma | MOL001601 | 1,2,5,6-tetrahydrotanshinone | CHRM5 |  |  |  |  |  |  |
| Salviae Miltiorrhizae Radix et Rhizoma | MOL001601 | 1,2,5,6-tetrahydrotanshinone | PTGS2 |  |  |  |  |  |  |
| Salviae Miltiorrhizae Radix et Rhizoma | MOL001601 | 1,2,5,6-tetrahydrotanshinone | HTR3A |  |  |  |  |  |  |
| Salviae Miltiorrhizae Radix et Rhizoma | MOL001601 | 1,2,5,6-tetrahydrotanshinone | CA2 |  |  |  |  |  |  |
| Salviae Miltiorrhizae Radix et Rhizoma | MOL001601 | 1,2,5,6-tetrahydrotanshinone | CHRM4 |  |  |  |  |  |  |
| Salviae Miltiorrhizae Radix et Rhizoma | MOL001601 | 1,2,5,6-tetrahydrotanshinone | RXRA |  |  |  |  |  |  |
| Salviae Miltiorrhizae Radix et Rhizoma | MOL001601 | 1,2,5,6-tetrahydrotanshinone | OPRD1 |  |  |  |  |  |  |
| Salviae Miltiorrhizae Radix et Rhizoma | MOL001601 | 1,2,5,6-tetrahydrotanshinone | ADRA1A |  |  |  |  |  |  |
| Salviae Miltiorrhizae Radix et Rhizoma | MOL001601 | 1,2,5,6-tetrahydrotanshinone | CHRM2 |  |  |  |  |  |  |
| Salviae Miltiorrhizae Radix et Rhizoma | MOL001601 | 1,2,5,6-tetrahydrotanshinone | ADRA1B |  |  |  |  |  |  |
| Salviae Miltiorrhizae Radix et Rhizoma | MOL001601 | 1,2,5,6-tetrahydrotanshinone | SLC6A3 |  |  |  |  |  |  |
| Salviae Miltiorrhizae Radix et Rhizoma | MOL001601 | 1,2,5,6-tetrahydrotanshinone | ADRB2 |  |  |  |  |  |  |
| Salviae Miltiorrhizae Radix et Rhizoma | MOL001601 | 1,2,5,6-tetrahydrotanshinone | ADRA1D |  |  |  |  |  |  |
| Salviae Miltiorrhizae Radix et Rhizoma | MOL001601 | 1,2,5,6-tetrahydrotanshinone | OPRM1 |  |  |  |  |  |  |
| Salviae Miltiorrhizae Radix et Rhizoma | MOL001601 | 1,2,5,6-tetrahydrotanshinone | GABRA1 |  |  |  |  |  |  |
| Salviae Miltiorrhizae Radix et Rhizoma | MOL001601 | 1,2,5,6-tetrahydrotanshinone | HSP90AA1 |  |  |  |  |  |  |
| Salviae Miltiorrhizae Radix et Rhizoma | MOL001601 | 1,2,5,6-tetrahydrotanshinone | NCOA2 |  |  |  |  |  |  |
| Salviae Miltiorrhizae Radix et Rhizoma | MOL001601 | 1,2,5,6-tetrahydrotanshinone | NCOA1 |  |  |  |  |  |  |
| Salviae Miltiorrhizae Radix et Rhizoma | MOL001601 | 1,2,5,6-tetrahydrotanshinone | DRD1 |  |  |  |  |  |  |
| Salviae Miltiorrhizae Radix et Rhizoma | MOL001601 | 1,2,5,6-tetrahydrotanshinone | SLC6A4 |  |  |  |  |  |  |
| Salviae Miltiorrhizae Radix et Rhizoma | MOL001601 | 1,2,5,6-tetrahydrotanshinone | IGHG1 |  |  |  |  |  |  |
| Salviae Miltiorrhizae Radix et Rhizoma | MOL001659 | Poriferasterol | PGR |  |  |  |  |  |  |
| Salviae Miltiorrhizae Radix et Rhizoma | MOL001659 | Poriferasterol | NR3C2 |  |  |  |  |  |  |
| Salviae Miltiorrhizae Radix et Rhizoma | MOL001771 | poriferast-5-en-3beta-ol | PGR |  |  |  |  |  |  |
| Salviae Miltiorrhizae Radix et Rhizoma | MOL001771 | poriferast-5-en-3beta-ol | NCOA2 |  |  |  |  |  |  |
| Salviae Miltiorrhizae Radix et Rhizoma | MOL001942 | isoimperatorin | PTGS2 |  |  |  |  |  |  |
| Salviae Miltiorrhizae Radix et Rhizoma | MOL002222 | sugiol | CHRM3 |  |  |  |  |  |  |
| Salviae Miltiorrhizae Radix et Rhizoma | MOL002222 | sugiol | CHRM1 |  |  |  |  |  |  |
| Salviae Miltiorrhizae Radix et Rhizoma | MOL002222 | sugiol | SCN5A |  |  |  |  |  |  |
| Salviae Miltiorrhizae Radix et Rhizoma | MOL002222 | sugiol | CHRM5 |  |  |  |  |  |  |
| Salviae Miltiorrhizae Radix et Rhizoma | MOL002222 | sugiol | PTGS2 |  |  |  |  |  |  |
| Salviae Miltiorrhizae Radix et Rhizoma | MOL002222 | sugiol | CA2 |  |  |  |  |  |  |
| Salviae Miltiorrhizae Radix et Rhizoma | MOL002222 | sugiol | CHRM4 |  |  |  |  |  |  |
| Salviae Miltiorrhizae Radix et Rhizoma | MOL002222 | sugiol | OPRD1 |  |  |  |  |  |  |
| Salviae Miltiorrhizae Radix et Rhizoma | MOL002222 | sugiol | ACHE |  |  |  |  |  |  |
| Salviae Miltiorrhizae Radix et Rhizoma | MOL002222 | sugiol | ADRA1A |  |  |  |  |  |  |
| Salviae Miltiorrhizae Radix et Rhizoma | MOL002222 | sugiol | CHRM2 |  |  |  |  |  |  |
| Salviae Miltiorrhizae Radix et Rhizoma | MOL002222 | sugiol | ADRA1B |  |  |  |  |  |  |
| Salviae Miltiorrhizae Radix et Rhizoma | MOL002222 | sugiol | ADRB2 |  |  |  |  |  |  |
| Salviae Miltiorrhizae Radix et Rhizoma | MOL002222 | sugiol | ADRA1D |  |  |  |  |  |  |
| Salviae Miltiorrhizae Radix et Rhizoma | MOL002222 | sugiol | DRD2 |  |  |  |  |  |  |
| Salviae Miltiorrhizae Radix et Rhizoma | MOL002222 | sugiol | OPRM1 |  |  |  |  |  |  |
| Salviae Miltiorrhizae Radix et Rhizoma | MOL002651 | Dehydrotanshinone II A | DRD1 |  |  |  |  |  |  |
| Salviae Miltiorrhizae Radix et Rhizoma | MOL002651 | Dehydrotanshinone II A | CHRM3 |  |  |  |  |  |  |
| Salviae Miltiorrhizae Radix et Rhizoma | MOL002651 | Dehydrotanshinone II A | HTR |  |  |  |  |  |  |
| Salviae Miltiorrhizae Radix et Rhizoma | MOL002651 | Dehydrotanshinone II A | CHRM1 |  |  |  |  |  |  |
| Salviae Miltiorrhizae Radix et Rhizoma | MOL002651 | Dehydrotanshinone II A | ESR1 |  |  |  |  |  |  |
| Salviae Miltiorrhizae Radix et Rhizoma | MOL002651 | Dehydrotanshinone II A | AR |  |  |  |  |  |  |
| Salviae Miltiorrhizae Radix et Rhizoma | MOL002651 | Dehydrotanshinone II A | SCN5A |  |  |  |  |  |  |
| Salviae Miltiorrhizae Radix et Rhizoma | MOL002651 | Dehydrotanshinone II A | PPARG |  |  |  |  |  |  |
| Salviae Miltiorrhizae Radix et Rhizoma | MOL002651 | Dehydrotanshinone II A | CHRM5 |  |  |  |  |  |  |
| Salviae Miltiorrhizae Radix et Rhizoma | MOL002651 | Dehydrotanshinone II A | PTGS2 |  |  |  |  |  |  |
| Salviae Miltiorrhizae Radix et Rhizoma | MOL002651 | Dehydrotanshinone II A | CHRM4 |  |  |  |  |  |  |
| Salviae Miltiorrhizae Radix et Rhizoma | MOL002651 | Dehydrotanshinone II A | OPRD1 |  |  |  |  |  |  |
| Salviae Miltiorrhizae Radix et Rhizoma | MOL002651 | Dehydrotanshinone II A | ACHE |  |  |  |  |  |  |
| Salviae Miltiorrhizae Radix et Rhizoma | MOL002651 | Dehydrotanshinone II A | ADRA1A |  |  |  |  |  |  |
| Salviae Miltiorrhizae Radix et Rhizoma | MOL002651 | Dehydrotanshinone II A | ADRB2 |  |  |  |  |  |  |
| Salviae Miltiorrhizae Radix et Rhizoma | MOL002651 | Dehydrotanshinone II A | OPRM1 |  |  |  |  |  |  |
| Salviae Miltiorrhizae Radix et Rhizoma | MOL002651 | Dehydrotanshinone II A | GABRA1 |  |  |  |  |  |  |
| Salviae Miltiorrhizae Radix et Rhizoma | MOL002651 | Dehydrotanshinone II A | DPP4 |  |  |  |  |  |  |
| Salviae Miltiorrhizae Radix et Rhizoma | MOL002651 | Dehydrotanshinone II A | NCOA1 |  |  |  |  |  |  |
| Salviae Miltiorrhizae Radix et Rhizoma | MOL000569 | digallate | PTGS2 |  |  |  |  |  |  |
| Salviae Miltiorrhizae Radix et Rhizoma | MOL000569 | digallate | HSP90AA1 |  |  |  |  |  |  |
| Salviae Miltiorrhizae Radix et Rhizoma | MOL000569 | digallate | AKR1B1 |  |  |  |  |  |  |
| Salviae Miltiorrhizae Radix et Rhizoma | MOL000006 | luteolin | PTGS1 |  |  |  |  |  |  |
| Salviae Miltiorrhizae Radix et Rhizoma | MOL000006 | luteolin | AR |  |  |  |  |  |  |
| Salviae Miltiorrhizae Radix et Rhizoma | MOL000006 | luteolin | PTGS2 |  |  |  |  |  |  |
| Salviae Miltiorrhizae Radix et Rhizoma | MOL000006 | luteolin | HSP90AA1 |  |  |  |  |  |  |
| Salviae Miltiorrhizae Radix et Rhizoma | MOL000006 | luteolin | PRSS1 |  |  |  |  |  |  |
| Salviae Miltiorrhizae Radix et Rhizoma | MOL000006 | luteolin | NCOA2 |  |  |  |  |  |  |
| Salviae Miltiorrhizae Radix et Rhizoma | MOL000006 | luteolin | DPP4 |  |  |  |  |  |  |
| Salviae Miltiorrhizae Radix et Rhizoma | MOL000006 | luteolin | RELA |  |  |  |  |  |  |
| Salviae Miltiorrhizae Radix et Rhizoma | MOL000006 | luteolin | EGFR |  |  |  |  |  |  |
| Salviae Miltiorrhizae Radix et Rhizoma | MOL000006 | luteolin | AKT1 |  |  |  |  |  |  |
| Salviae Miltiorrhizae Radix et Rhizoma | MOL000006 | luteolin | VEGFA |  |  |  |  |  |  |
| Salviae Miltiorrhizae Radix et Rhizoma | MOL000006 | luteolin | CCND1 |  |  |  |  |  |  |
| Salviae Miltiorrhizae Radix et Rhizoma | MOL000006 | luteolin | BCL2L1 |  |  |  |  |  |  |
| Salviae Miltiorrhizae Radix et Rhizoma | MOL000006 | luteolin | CDKN1A |  |  |  |  |  |  |
| Salviae Miltiorrhizae Radix et Rhizoma | MOL000006 | luteolin | CASP9 |  |  |  |  |  |  |
| Salviae Miltiorrhizae Radix et Rhizoma | MOL000006 | luteolin | MMP2 |  |  |  |  |  |  |
| Salviae Miltiorrhizae Radix et Rhizoma | MOL000006 | luteolin | MMP9 |  |  |  |  |  |  |
| Salviae Miltiorrhizae Radix et Rhizoma | MOL000006 | luteolin | MAPK1 |  |  |  |  |  |  |
| Salviae Miltiorrhizae Radix et Rhizoma | MOL000006 | luteolin | IL10RB |  |  |  |  |  |  |
| Salviae Miltiorrhizae Radix et Rhizoma | MOL000006 | luteolin | RB1 |  |  |  |  |  |  |
| Salviae Miltiorrhizae Radix et Rhizoma | MOL000006 | luteolin | CDK4 |  |  |  |  |  |  |
| Salviae Miltiorrhizae Radix et Rhizoma | MOL000006 | luteolin | TNFSF15 |  |  |  |  |  |  |
| Salviae Miltiorrhizae Radix et Rhizoma | MOL000006 | luteolin | JUN |  |  |  |  |  |  |
| Salviae Miltiorrhizae Radix et Rhizoma | MOL000006 | luteolin | IL6 |  |  |  |  |  |  |
| Salviae Miltiorrhizae Radix et Rhizoma | MOL000006 | luteolin | CASP3 |  |  |  |  |  |  |
| Salviae Miltiorrhizae Radix et Rhizoma | MOL000006 | luteolin | TP63 |  |  |  |  |  |  |
| Salviae Miltiorrhizae Radix et Rhizoma | MOL000006 | luteolin | NFKBIA |  |  |  |  |  |  |
| Salviae Miltiorrhizae Radix et Rhizoma | MOL000006 | luteolin | TOP1 |  |  |  |  |  |  |
| Salviae Miltiorrhizae Radix et Rhizoma | MOL000006 | luteolin | MDM2 |  |  |  |  |  |  |
| Salviae Miltiorrhizae Radix et Rhizoma | MOL000006 | luteolin | APP |  |  |  |  |  |  |
| Salviae Miltiorrhizae Radix et Rhizoma | MOL000006 | luteolin | MMP1 |  |  |  |  |  |  |
| Salviae Miltiorrhizae Radix et Rhizoma | MOL000006 | luteolin | PCNA |  |  |  |  |  |  |
| Salviae Miltiorrhizae Radix et Rhizoma | MOL000006 | luteolin | ERBB2 |  |  |  |  |  |  |
| Salviae Miltiorrhizae Radix et Rhizoma | MOL000006 | luteolin | PPARG |  |  |  |  |  |  |
| Salviae Miltiorrhizae Radix et Rhizoma | MOL000006 | luteolin | HMOX1 |  |  |  |  |  |  |
| Salviae Miltiorrhizae Radix et Rhizoma | MOL000006 | luteolin | CASP7 |  |  |  |  |  |  |
| Salviae Miltiorrhizae Radix et Rhizoma | MOL000006 | luteolin | ICAM1 |  |  |  |  |  |  |
| Salviae Miltiorrhizae Radix et Rhizoma | MOL000006 | luteolin | MCL1 |  |  |  |  |  |  |
| Salviae Miltiorrhizae Radix et Rhizoma | MOL000006 | luteolin | BIRC5 |  |  |  |  |  |  |
| Salviae Miltiorrhizae Radix et Rhizoma | MOL000006 | luteolin | IL2 |  |  |  |  |  |  |
| Salviae Miltiorrhizae Radix et Rhizoma | MOL000006 | luteolin | CCNB1 |  |  |  |  |  |  |
| Salviae Miltiorrhizae Radix et Rhizoma | MOL000006 | luteolin | TYR |  |  |  |  |  |  |
| Salviae Miltiorrhizae Radix et Rhizoma | MOL000006 | luteolin | IFNGR1 |  |  |  |  |  |  |
| Salviae Miltiorrhizae Radix et Rhizoma | MOL000006 | luteolin | IL4 |  |  |  |  |  |  |
| Salviae Miltiorrhizae Radix et Rhizoma | MOL000006 | luteolin | TOP2A |  |  |  |  |  |  |
| Salviae Miltiorrhizae Radix et Rhizoma | MOL000006 | luteolin | GSTP1 |  |  |  |  |  |  |
| Salviae Miltiorrhizae Radix et Rhizoma | MOL000006 | luteolin | XIAP |  |  |  |  |  |  |
| Salviae Miltiorrhizae Radix et Rhizoma | MOL000006 | luteolin | SLC2A4 |  |  |  |  |  |  |
| Salviae Miltiorrhizae Radix et Rhizoma | MOL000006 | luteolin | INSRR |  |  |  |  |  |  |
| Salviae Miltiorrhizae Radix et Rhizoma | MOL000006 | luteolin | CD40LG |  |  |  |  |  |  |
| Salviae Miltiorrhizae Radix et Rhizoma | MOL000006 | luteolin | PTGES |  |  |  |  |  |  |
| Salviae Miltiorrhizae Radix et Rhizoma | MOL000006 | luteolin | NUF2 |  |  |  |  |  |  |
| Salviae Miltiorrhizae Radix et Rhizoma | MOL000006 | luteolin | ADCY2 |  |  |  |  |  |  |
| Salviae Miltiorrhizae Radix et Rhizoma | MOL000006 | luteolin | MET |  |  |  |  |  |  |
| Salviae Miltiorrhizae Radix et Rhizoma | MOL007036 | 5,6-dihydroxy-7-isopropyl-1,1-dimethyl-2,3-dihydrophenanthren-4-one | PTGS1 |  |  |  |  |  |  |
| Salviae Miltiorrhizae Radix et Rhizoma | MOL007036 | 5,6-dihydroxy-7-isopropyl-1,1-dimethyl-2,3-dihydrophenanthren-4-one | CHRM3 |  |  |  |  |  |  |
| Salviae Miltiorrhizae Radix et Rhizoma | MOL007036 | 5,6-dihydroxy-7-isopropyl-1,1-dimethyl-2,3-dihydrophenanthren-4-one | CHRM1 |  |  |  |  |  |  |
| Salviae Miltiorrhizae Radix et Rhizoma | MOL007036 | 5,6-dihydroxy-7-isopropyl-1,1-dimethyl-2,3-dihydrophenanthren-4-one | SCN5A |  |  |  |  |  |  |
| Salviae Miltiorrhizae Radix et Rhizoma | MOL007036 | 5,6-dihydroxy-7-isopropyl-1,1-dimethyl-2,3-dihydrophenanthren-4-one | PTGS2 |  |  |  |  |  |  |
| Salviae Miltiorrhizae Radix et Rhizoma | MOL007036 | 5,6-dihydroxy-7-isopropyl-1,1-dimethyl-2,3-dihydrophenanthren-4-one | CA2 |  |  |  |  |  |  |
| Salviae Miltiorrhizae Radix et Rhizoma | MOL007036 | 5,6-dihydroxy-7-isopropyl-1,1-dimethyl-2,3-dihydrophenanthren-4-one | RXRA |  |  |  |  |  |  |
| Salviae Miltiorrhizae Radix et Rhizoma | MOL007036 | 5,6-dihydroxy-7-isopropyl-1,1-dimethyl-2,3-dihydrophenanthren-4-one | ACHE |  |  |  |  |  |  |
| Salviae Miltiorrhizae Radix et Rhizoma | MOL007036 | 5,6-dihydroxy-7-isopropyl-1,1-dimethyl-2,3-dihydrophenanthren-4-one | ADRA1A |  |  |  |  |  |  |
| Salviae Miltiorrhizae Radix et Rhizoma | MOL007036 | 5,6-dihydroxy-7-isopropyl-1,1-dimethyl-2,3-dihydrophenanthren-4-one | ADRA1B |  |  |  |  |  |  |
| Salviae Miltiorrhizae Radix et Rhizoma | MOL007036 | 5,6-dihydroxy-7-isopropyl-1,1-dimethyl-2,3-dihydrophenanthren-4-one | ADRB2 |  |  |  |  |  |  |
| Salviae Miltiorrhizae Radix et Rhizoma | MOL007036 | 5,6-dihydroxy-7-isopropyl-1,1-dimethyl-2,3-dihydrophenanthren-4-one | OPRM1 |  |  |  |  |  |  |
| Salviae Miltiorrhizae Radix et Rhizoma | MOL007036 | 5,6-dihydroxy-7-isopropyl-1,1-dimethyl-2,3-dihydrophenanthren-4-one | IGHG1 |  |  |  |  |  |  |
| Salviae Miltiorrhizae Radix et Rhizoma | MOL007036 | 5,6-dihydroxy-7-isopropyl-1,1-dimethyl-2,3-dihydrophenanthren-4-one | NCOA2 |  |  |  |  |  |  |
| Salviae Miltiorrhizae Radix et Rhizoma | MOL007036 | 5,6-dihydroxy-7-isopropyl-1,1-dimethyl-2,3-dihydrophenanthren-4-one | NCOA1 |  |  |  |  |  |  |
| Salviae Miltiorrhizae Radix et Rhizoma | MOL007036 | 5,6-dihydroxy-7-isopropyl-1,1-dimethyl-2,3-dihydrophenanthren-4-one | CALM1 |  |  |  |  |  |  |
| Salviae Miltiorrhizae Radix et Rhizoma | MOL007041 | 2-isopropyl-8-methylphenanthrene-3,4-dione | PTGS1 |  |  |  |  |  |  |
| Salviae Miltiorrhizae Radix et Rhizoma | MOL007041 | 2-isopropyl-8-methylphenanthrene-3,4-dione | DRD1 |  |  |  |  |  |  |
| Salviae Miltiorrhizae Radix et Rhizoma | MOL007041 | 2-isopropyl-8-methylphenanthrene-3,4-dione | CHRM3 |  |  |  |  |  |  |
| Salviae Miltiorrhizae Radix et Rhizoma | MOL007041 | 2-isopropyl-8-methylphenanthrene-3,4-dione | CHRM1 |  |  |  |  |  |  |
| Salviae Miltiorrhizae Radix et Rhizoma | MOL007041 | 2-isopropyl-8-methylphenanthrene-3,4-dione | ESR1 |  |  |  |  |  |  |
| Salviae Miltiorrhizae Radix et Rhizoma | MOL007041 | 2-isopropyl-8-methylphenanthrene-3,4-dione | AR |  |  |  |  |  |  |
| Salviae Miltiorrhizae Radix et Rhizoma | MOL007041 | 2-isopropyl-8-methylphenanthrene-3,4-dione | SCN5A |  |  |  |  |  |  |
| Salviae Miltiorrhizae Radix et Rhizoma | MOL007041 | 2-isopropyl-8-methylphenanthrene-3,4-dione | PPARG |  |  |  |  |  |  |
| Salviae Miltiorrhizae Radix et Rhizoma | MOL007041 | 2-isopropyl-8-methylphenanthrene-3,4-dione | CHRM5 |  |  |  |  |  |  |
| Salviae Miltiorrhizae Radix et Rhizoma | MOL007041 | 2-isopropyl-8-methylphenanthrene-3,4-dione | PTGS2 |  |  |  |  |  |  |
| Salviae Miltiorrhizae Radix et Rhizoma | MOL007041 | 2-isopropyl-8-methylphenanthrene-3,4-dione | HTR3A |  |  |  |  |  |  |
| Salviae Miltiorrhizae Radix et Rhizoma | MOL007041 | 2-isopropyl-8-methylphenanthrene-3,4-dione | CHRM4 |  |  |  |  |  |  |
| Salviae Miltiorrhizae Radix et Rhizoma | MOL007041 | 2-isopropyl-8-methylphenanthrene-3,4-dione | RXRA |  |  |  |  |  |  |
| Salviae Miltiorrhizae Radix et Rhizoma | MOL007041 | 2-isopropyl-8-methylphenanthrene-3,4-dione | ADRA1A |  |  |  |  |  |  |
| Salviae Miltiorrhizae Radix et Rhizoma | MOL007041 | 2-isopropyl-8-methylphenanthrene-3,4-dione | CHRM2 |  |  |  |  |  |  |
| Salviae Miltiorrhizae Radix et Rhizoma | MOL007041 | 2-isopropyl-8-methylphenanthrene-3,4-dione | ADRA1B |  |  |  |  |  |  |
| Salviae Miltiorrhizae Radix et Rhizoma | MOL007041 | 2-isopropyl-8-methylphenanthrene-3,4-dione | SLC6A3 |  |  |  |  |  |  |
| Salviae Miltiorrhizae Radix et Rhizoma | MOL007041 | 2-isopropyl-8-methylphenanthrene-3,4-dione | ADRB2 |  |  |  |  |  |  |
| Salviae Miltiorrhizae Radix et Rhizoma | MOL007041 | 2-isopropyl-8-methylphenanthrene-3,4-dione | ADRA1D |  |  |  |  |  |  |
| Salviae Miltiorrhizae Radix et Rhizoma | MOL007041 | 2-isopropyl-8-methylphenanthrene-3,4-dione | SLC6A4 |  |  |  |  |  |  |
| Salviae Miltiorrhizae Radix et Rhizoma | MOL007041 | 2-isopropyl-8-methylphenanthrene-3,4-dione | OPRM1 |  |  |  |  |  |  |
| Salviae Miltiorrhizae Radix et Rhizoma | MOL007041 | 2-isopropyl-8-methylphenanthrene-3,4-dione | GABRA1 |  |  |  |  |  |  |
| Salviae Miltiorrhizae Radix et Rhizoma | MOL007041 | 2-isopropyl-8-methylphenanthrene-3,4-dione | CDK2 |  |  |  |  |  |  |
| Salviae Miltiorrhizae Radix et Rhizoma | MOL007041 | 2-isopropyl-8-methylphenanthrene-3,4-dione | IGHG1 |  |  |  |  |  |  |
| Salviae Miltiorrhizae Radix et Rhizoma | MOL007041 | 2-isopropyl-8-methylphenanthrene-3,4-dione | CCNA2 |  |  |  |  |  |  |
| Salviae Miltiorrhizae Radix et Rhizoma | MOL007041 | 2-isopropyl-8-methylphenanthrene-3,4-dione | NCOA2 |  |  |  |  |  |  |
| Salviae Miltiorrhizae Radix et Rhizoma | MOL007041 | 2-isopropyl-8-methylphenanthrene-3,4-dione | CALM1 |  |  |  |  |  |  |
| Salviae Miltiorrhizae Radix et Rhizoma | MOL007045 | 3α-hydroxytanshinoneⅡa | HTR |  |  |  |  |  |  |
| Salviae Miltiorrhizae Radix et Rhizoma | MOL007045 | 3α-hydroxytanshinoneⅡa | CHRM1 |  |  |  |  |  |  |
| Salviae Miltiorrhizae Radix et Rhizoma | MOL007045 | 3α-hydroxytanshinoneⅡa | SCN5A |  |  |  |  |  |  |
| Salviae Miltiorrhizae Radix et Rhizoma | MOL007045 | 3α-hydroxytanshinoneⅡa | CHRM5 |  |  |  |  |  |  |
| Salviae Miltiorrhizae Radix et Rhizoma | MOL007045 | 3α-hydroxytanshinoneⅡa | PTGS2 |  |  |  |  |  |  |
| Salviae Miltiorrhizae Radix et Rhizoma | MOL007045 | 3α-hydroxytanshinoneⅡa | OPRD1 |  |  |  |  |  |  |
| Salviae Miltiorrhizae Radix et Rhizoma | MOL007045 | 3α-hydroxytanshinoneⅡa | ACHE |  |  |  |  |  |  |
| Salviae Miltiorrhizae Radix et Rhizoma | MOL007045 | 3α-hydroxytanshinoneⅡa | ADRB2 |  |  |  |  |  |  |
| Salviae Miltiorrhizae Radix et Rhizoma | MOL007045 | 3α-hydroxytanshinoneⅡa | OPRM1 |  |  |  |  |  |  |
| Salviae Miltiorrhizae Radix et Rhizoma | MOL007045 | 3α-hydroxytanshinoneⅡa | DPP4 |  |  |  |  |  |  |
| Salviae Miltiorrhizae Radix et Rhizoma | MOL007045 | 3α-hydroxytanshinoneⅡa | PRSS1 |  |  |  |  |  |  |
| Salviae Miltiorrhizae Radix et Rhizoma | MOL007045 | 3α-hydroxytanshinoneⅡa | NCOA1 |  |  |  |  |  |  |
| Salviae Miltiorrhizae Radix et Rhizoma | MOL007048 | (E)-3-[2-(3,4-dihydroxyphenyl)-7-hydroxy-benzofuran-4-yl]acrylic acid | PTGS2 |  |  |  |  |  |  |
| Salviae Miltiorrhizae Radix et Rhizoma | MOL007048 | (E)-3-[2-(3,4-dihydroxyphenyl)-7-hydroxy-benzofuran-4-yl]acrylic acid | HSP90AA1 |  |  |  |  |  |  |
| Salviae Miltiorrhizae Radix et Rhizoma | MOL007049 | 4-methylenemiltirone | PTGS1 |  |  |  |  |  |  |
| Salviae Miltiorrhizae Radix et Rhizoma | MOL007049 | 4-methylenemiltirone | DRD1 |  |  |  |  |  |  |
| Salviae Miltiorrhizae Radix et Rhizoma | MOL007049 | 4-methylenemiltirone | CHRM3 |  |  |  |  |  |  |
| Salviae Miltiorrhizae Radix et Rhizoma | MOL007049 | 4-methylenemiltirone | CHRM1 |  |  |  |  |  |  |
| Salviae Miltiorrhizae Radix et Rhizoma | MOL007049 | 4-methylenemiltirone | ESR1 |  |  |  |  |  |  |
| Salviae Miltiorrhizae Radix et Rhizoma | MOL007049 | 4-methylenemiltirone | AR |  |  |  |  |  |  |
| Salviae Miltiorrhizae Radix et Rhizoma | MOL007049 | 4-methylenemiltirone | SCN5A |  |  |  |  |  |  |
| Salviae Miltiorrhizae Radix et Rhizoma | MOL007049 | 4-methylenemiltirone | PPARG |  |  |  |  |  |  |
| Salviae Miltiorrhizae Radix et Rhizoma | MOL007049 | 4-methylenemiltirone | CHRM5 |  |  |  |  |  |  |
| Salviae Miltiorrhizae Radix et Rhizoma | MOL007049 | 4-methylenemiltirone | PTGS2 |  |  |  |  |  |  |
| Salviae Miltiorrhizae Radix et Rhizoma | MOL007049 | 4-methylenemiltirone | ADRA2A |  |  |  |  |  |  |
| Salviae Miltiorrhizae Radix et Rhizoma | MOL007049 | 4-methylenemiltirone | CA2 |  |  |  |  |  |  |
| Salviae Miltiorrhizae Radix et Rhizoma | MOL007049 | 4-methylenemiltirone | ADRA2C |  |  |  |  |  |  |
| Salviae Miltiorrhizae Radix et Rhizoma | MOL007049 | 4-methylenemiltirone | CHRM4 |  |  |  |  |  |  |
| Salviae Miltiorrhizae Radix et Rhizoma | MOL007049 | 4-methylenemiltirone | RXRA |  |  |  |  |  |  |
| Salviae Miltiorrhizae Radix et Rhizoma | MOL007049 | 4-methylenemiltirone | OPRD1 |  |  |  |  |  |  |
| Salviae Miltiorrhizae Radix et Rhizoma | MOL007049 | 4-methylenemiltirone | ADRA1A |  |  |  |  |  |  |
| Salviae Miltiorrhizae Radix et Rhizoma | MOL007049 | 4-methylenemiltirone | CHRM2 |  |  |  |  |  |  |
| Salviae Miltiorrhizae Radix et Rhizoma | MOL007049 | 4-methylenemiltirone | ADRA1B |  |  |  |  |  |  |
| Salviae Miltiorrhizae Radix et Rhizoma | MOL007049 | 4-methylenemiltirone | SLC6A3 |  |  |  |  |  |  |
| Salviae Miltiorrhizae Radix et Rhizoma | MOL007049 | 4-methylenemiltirone | ADRB2 |  |  |  |  |  |  |
| Salviae Miltiorrhizae Radix et Rhizoma | MOL007049 | 4-methylenemiltirone | ADRA1D |  |  |  |  |  |  |
| Salviae Miltiorrhizae Radix et Rhizoma | MOL007049 | 4-methylenemiltirone | SLC6A4 |  |  |  |  |  |  |
| Salviae Miltiorrhizae Radix et Rhizoma | MOL007049 | 4-methylenemiltirone | DRD2 |  |  |  |  |  |  |
| Salviae Miltiorrhizae Radix et Rhizoma | MOL007049 | 4-methylenemiltirone | OPRM1 |  |  |  |  |  |  |
| Salviae Miltiorrhizae Radix et Rhizoma | MOL007049 | 4-methylenemiltirone | GABRA1 |  |  |  |  |  |  |
| Salviae Miltiorrhizae Radix et Rhizoma | MOL007049 | 4-methylenemiltirone | NCOA2 |  |  |  |  |  |  |
| Salviae Miltiorrhizae Radix et Rhizoma | MOL007049 | 4-methylenemiltirone | NCOA1 |  |  |  |  |  |  |
| Salviae Miltiorrhizae Radix et Rhizoma | MOL007050 | 2-(4-hydroxy-3-methoxyphenyl)-5-(3-hydroxypropyl)-7-methoxy-3-benzofurancarboxaldehyde | NOS2 |  |  |  |  |  |  |
| Salviae Miltiorrhizae Radix et Rhizoma | MOL007050 | 2-(4-hydroxy-3-methoxyphenyl)-5-(3-hydroxypropyl)-7-methoxy-3-benzofurancarboxaldehyde | HTR |  |  |  |  |  |  |
| Salviae Miltiorrhizae Radix et Rhizoma | MOL007050 | 2-(4-hydroxy-3-methoxyphenyl)-5-(3-hydroxypropyl)-7-methoxy-3-benzofurancarboxaldehyde | ESR1 |  |  |  |  |  |  |
| Salviae Miltiorrhizae Radix et Rhizoma | MOL007050 | 2-(4-hydroxy-3-methoxyphenyl)-5-(3-hydroxypropyl)-7-methoxy-3-benzofurancarboxaldehyde | AR |  |  |  |  |  |  |
| Salviae Miltiorrhizae Radix et Rhizoma | MOL007050 | 2-(4-hydroxy-3-methoxyphenyl)-5-(3-hydroxypropyl)-7-methoxy-3-benzofurancarboxaldehyde | PPARG |  |  |  |  |  |  |
| Salviae Miltiorrhizae Radix et Rhizoma | MOL007050 | 2-(4-hydroxy-3-methoxyphenyl)-5-(3-hydroxypropyl)-7-methoxy-3-benzofurancarboxaldehyde | ESR2 |  |  |  |  |  |  |
| Salviae Miltiorrhizae Radix et Rhizoma | MOL007050 | 2-(4-hydroxy-3-methoxyphenyl)-5-(3-hydroxypropyl)-7-methoxy-3-benzofurancarboxaldehyde | MAPK14 |  |  |  |  |  |  |
| Salviae Miltiorrhizae Radix et Rhizoma | MOL007050 | 2-(4-hydroxy-3-methoxyphenyl)-5-(3-hydroxypropyl)-7-methoxy-3-benzofurancarboxaldehyde | GSK3B |  |  |  |  |  |  |
| Salviae Miltiorrhizae Radix et Rhizoma | MOL007050 | 2-(4-hydroxy-3-methoxyphenyl)-5-(3-hydroxypropyl)-7-methoxy-3-benzofurancarboxaldehyde | HSP90AA1 |  |  |  |  |  |  |
| Salviae Miltiorrhizae Radix et Rhizoma | MOL007050 | 2-(4-hydroxy-3-methoxyphenyl)-5-(3-hydroxypropyl)-7-methoxy-3-benzofurancarboxaldehyde | CDK2 |  |  |  |  |  |  |
| Salviae Miltiorrhizae Radix et Rhizoma | MOL007050 | 2-(4-hydroxy-3-methoxyphenyl)-5-(3-hydroxypropyl)-7-methoxy-3-benzofurancarboxaldehyde | CCNA2 |  |  |  |  |  |  |
| Salviae Miltiorrhizae Radix et Rhizoma | MOL007058 | formyltanshinone | HTR |  |  |  |  |  |  |
| Salviae Miltiorrhizae Radix et Rhizoma | MOL007058 | formyltanshinone | AR |  |  |  |  |  |  |
| Salviae Miltiorrhizae Radix et Rhizoma | MOL007058 | formyltanshinone | PTGS2 |  |  |  |  |  |  |
| Salviae Miltiorrhizae Radix et Rhizoma | MOL007058 | formyltanshinone | RXRA |  |  |  |  |  |  |
| Salviae Miltiorrhizae Radix et Rhizoma | MOL007058 | formyltanshinone | DPP4 |  |  |  |  |  |  |
| Salviae Miltiorrhizae Radix et Rhizoma | MOL007058 | formyltanshinone | NCOA1 |  |  |  |  |  |  |
| Salviae Miltiorrhizae Radix et Rhizoma | MOL007059 | 3-beta-Hydroxymethyllenetanshiquinone | DRD1 |  |  |  |  |  |  |
| Salviae Miltiorrhizae Radix et Rhizoma | MOL007059 | 3-beta-Hydroxymethyllenetanshiquinone | HTR |  |  |  |  |  |  |
| Salviae Miltiorrhizae Radix et Rhizoma | MOL007059 | 3-beta-Hydroxymethyllenetanshiquinone | CHRM1 |  |  |  |  |  |  |
| Salviae Miltiorrhizae Radix et Rhizoma | MOL007059 | 3-beta-Hydroxymethyllenetanshiquinone | PTGS2 |  |  |  |  |  |  |
| Salviae Miltiorrhizae Radix et Rhizoma | MOL007059 | 3-beta-Hydroxymethyllenetanshiquinone | CA2 |  |  |  |  |  |  |
| Salviae Miltiorrhizae Radix et Rhizoma | MOL007059 | 3-beta-Hydroxymethyllenetanshiquinone | RXRA |  |  |  |  |  |  |
| Salviae Miltiorrhizae Radix et Rhizoma | MOL007059 | 3-beta-Hydroxymethyllenetanshiquinone | OPRD1 |  |  |  |  |  |  |
| Salviae Miltiorrhizae Radix et Rhizoma | MOL007059 | 3-beta-Hydroxymethyllenetanshiquinone | ACHE |  |  |  |  |  |  |
| Salviae Miltiorrhizae Radix et Rhizoma | MOL007059 | 3-beta-Hydroxymethyllenetanshiquinone | ADRA1A |  |  |  |  |  |  |
| Salviae Miltiorrhizae Radix et Rhizoma | MOL007059 | 3-beta-Hydroxymethyllenetanshiquinone | ADRB2 |  |  |  |  |  |  |
| Salviae Miltiorrhizae Radix et Rhizoma | MOL007059 | 3-beta-Hydroxymethyllenetanshiquinone | OPRM1 |  |  |  |  |  |  |
| Salviae Miltiorrhizae Radix et Rhizoma | MOL007059 | 3-beta-Hydroxymethyllenetanshiquinone | DPP4 |  |  |  |  |  |  |
| Salviae Miltiorrhizae Radix et Rhizoma | MOL007059 | 3-beta-Hydroxymethyllenetanshiquinone | HSP90AA1 |  |  |  |  |  |  |
| Salviae Miltiorrhizae Radix et Rhizoma | MOL007059 | 3-beta-Hydroxymethyllenetanshiquinone | IGHG1 |  |  |  |  |  |  |
| Salviae Miltiorrhizae Radix et Rhizoma | MOL007059 | 3-beta-Hydroxymethyllenetanshiquinone | PRSS1 |  |  |  |  |  |  |
| Salviae Miltiorrhizae Radix et Rhizoma | MOL007059 | 3-beta-Hydroxymethyllenetanshiquinone | NCOA1 |  |  |  |  |  |  |
| Salviae Miltiorrhizae Radix et Rhizoma | MOL007061 | Methylenetanshinquinone | DRD1 |  |  |  |  |  |  |
| Salviae Miltiorrhizae Radix et Rhizoma | MOL007061 | Methylenetanshinquinone | CHRM3 |  |  |  |  |  |  |
| Salviae Miltiorrhizae Radix et Rhizoma | MOL007061 | Methylenetanshinquinone | HTR |  |  |  |  |  |  |
| Salviae Miltiorrhizae Radix et Rhizoma | MOL007061 | Methylenetanshinquinone | CHRM1 |  |  |  |  |  |  |
| Salviae Miltiorrhizae Radix et Rhizoma | MOL007061 | Methylenetanshinquinone | SCN5A |  |  |  |  |  |  |
| Salviae Miltiorrhizae Radix et Rhizoma | MOL007061 | Methylenetanshinquinone | CHRM5 |  |  |  |  |  |  |
| Salviae Miltiorrhizae Radix et Rhizoma | MOL007061 | Methylenetanshinquinone | PTGS2 |  |  |  |  |  |  |
| Salviae Miltiorrhizae Radix et Rhizoma | MOL007061 | Methylenetanshinquinone | CA2 |  |  |  |  |  |  |
| Salviae Miltiorrhizae Radix et Rhizoma | MOL007061 | Methylenetanshinquinone | RXRA |  |  |  |  |  |  |
| Salviae Miltiorrhizae Radix et Rhizoma | MOL007061 | Methylenetanshinquinone | OPRD1 |  |  |  |  |  |  |
| Salviae Miltiorrhizae Radix et Rhizoma | MOL007061 | Methylenetanshinquinone | ACHE |  |  |  |  |  |  |
| Salviae Miltiorrhizae Radix et Rhizoma | MOL007061 | Methylenetanshinquinone | ADRA1A |  |  |  |  |  |  |
| Salviae Miltiorrhizae Radix et Rhizoma | MOL007061 | Methylenetanshinquinone | CHRM2 |  |  |  |  |  |  |
| Salviae Miltiorrhizae Radix et Rhizoma | MOL007061 | Methylenetanshinquinone | ADRB2 |  |  |  |  |  |  |
| Salviae Miltiorrhizae Radix et Rhizoma | MOL007061 | Methylenetanshinquinone | SLC6A4 |  |  |  |  |  |  |
| Salviae Miltiorrhizae Radix et Rhizoma | MOL007061 | Methylenetanshinquinone | OPRM1 |  |  |  |  |  |  |
| Salviae Miltiorrhizae Radix et Rhizoma | MOL007061 | Methylenetanshinquinone | GABRA1 |  |  |  |  |  |  |
| Salviae Miltiorrhizae Radix et Rhizoma | MOL007061 | Methylenetanshinquinone | DPP4 |  |  |  |  |  |  |
| Salviae Miltiorrhizae Radix et Rhizoma | MOL007061 | Methylenetanshinquinone | HSP90AA1 |  |  |  |  |  |  |
| Salviae Miltiorrhizae Radix et Rhizoma | MOL007061 | Methylenetanshinquinone | IGHG1 |  |  |  |  |  |  |
| Salviae Miltiorrhizae Radix et Rhizoma | MOL007061 | Methylenetanshinquinone | PRSS1 |  |  |  |  |  |  |
| Salviae Miltiorrhizae Radix et Rhizoma | MOL007061 | Methylenetanshinquinone | NCOA1 |  |  |  |  |  |  |
| Salviae Miltiorrhizae Radix et Rhizoma | MOL007063 | przewalskin a | NR3C2 |  |  |  |  |  |  |
| Salviae Miltiorrhizae Radix et Rhizoma | MOL007063 | przewalskin a | NR3C1 |  |  |  |  |  |  |
| Salviae Miltiorrhizae Radix et Rhizoma | MOL007064 | przewalskin b | PTGS2 |  |  |  |  |  |  |
| Salviae Miltiorrhizae Radix et Rhizoma | MOL007064 | przewalskin b | PGR |  |  |  |  |  |  |
| Salviae Miltiorrhizae Radix et Rhizoma | MOL007064 | przewalskin b | NR3C2 |  |  |  |  |  |  |
| Salviae Miltiorrhizae Radix et Rhizoma | MOL007064 | przewalskin b | NR3C1 |  |  |  |  |  |  |
| Salviae Miltiorrhizae Radix et Rhizoma | MOL007064 | przewalskin b | NCOA2 |  |  |  |  |  |  |
| Salviae Miltiorrhizae Radix et Rhizoma | MOL007064 | przewalskin b | NCOA1 |  |  |  |  |  |  |
| Salviae Miltiorrhizae Radix et Rhizoma | MOL007068 | Przewaquinone B | HTR |  |  |  |  |  |  |
| Salviae Miltiorrhizae Radix et Rhizoma | MOL007068 | Przewaquinone B | PTGS2 |  |  |  |  |  |  |
| Salviae Miltiorrhizae Radix et Rhizoma | MOL007068 | Przewaquinone B | RXRA |  |  |  |  |  |  |
| Salviae Miltiorrhizae Radix et Rhizoma | MOL007068 | Przewaquinone B | DPP4 |  |  |  |  |  |  |
| Salviae Miltiorrhizae Radix et Rhizoma | MOL007068 | Przewaquinone B | HSP90AA1 |  |  |  |  |  |  |
| Salviae Miltiorrhizae Radix et Rhizoma | MOL007068 | Przewaquinone B | IGHG1 |  |  |  |  |  |  |
| Salviae Miltiorrhizae Radix et Rhizoma | MOL007068 | Przewaquinone B | PRSS1 |  |  |  |  |  |  |
| Salviae Miltiorrhizae Radix et Rhizoma | MOL007068 | Przewaquinone B | NCOA1 |  |  |  |  |  |  |
| Salviae Miltiorrhizae Radix et Rhizoma | MOL007069 | przewaquinone c | PTGS1 |  |  |  |  |  |  |
| Salviae Miltiorrhizae Radix et Rhizoma | MOL007069 | przewaquinone c | DRD1 |  |  |  |  |  |  |
| Salviae Miltiorrhizae Radix et Rhizoma | MOL007069 | przewaquinone c | CHRM3 |  |  |  |  |  |  |
| Salviae Miltiorrhizae Radix et Rhizoma | MOL007069 | przewaquinone c | HTR |  |  |  |  |  |  |
| Salviae Miltiorrhizae Radix et Rhizoma | MOL007069 | przewaquinone c | CHRM1 |  |  |  |  |  |  |
| Salviae Miltiorrhizae Radix et Rhizoma | MOL007069 | przewaquinone c | SCN5A |  |  |  |  |  |  |
| Salviae Miltiorrhizae Radix et Rhizoma | MOL007069 | przewaquinone c | CHRM5 |  |  |  |  |  |  |
| Salviae Miltiorrhizae Radix et Rhizoma | MOL007069 | przewaquinone c | PTGS2 |  |  |  |  |  |  |
| Salviae Miltiorrhizae Radix et Rhizoma | MOL007069 | przewaquinone c | CA2 |  |  |  |  |  |  |
| Salviae Miltiorrhizae Radix et Rhizoma | MOL007069 | przewaquinone c | CHRM4 |  |  |  |  |  |  |
| Salviae Miltiorrhizae Radix et Rhizoma | MOL007069 | przewaquinone c | OPRD1 |  |  |  |  |  |  |
| Salviae Miltiorrhizae Radix et Rhizoma | MOL007069 | przewaquinone c | ACHE |  |  |  |  |  |  |
| Salviae Miltiorrhizae Radix et Rhizoma | MOL007069 | przewaquinone c | ADRA1A |  |  |  |  |  |  |
| Salviae Miltiorrhizae Radix et Rhizoma | MOL007069 | przewaquinone c | CHRM2 |  |  |  |  |  |  |
| Salviae Miltiorrhizae Radix et Rhizoma | MOL007069 | przewaquinone c | ADRB2 |  |  |  |  |  |  |
| Salviae Miltiorrhizae Radix et Rhizoma | MOL007069 | przewaquinone c | OPRM1 |  |  |  |  |  |  |
| Salviae Miltiorrhizae Radix et Rhizoma | MOL007069 | przewaquinone c | GABRA1 |  |  |  |  |  |  |
| Salviae Miltiorrhizae Radix et Rhizoma | MOL007069 | przewaquinone c | DPP4 |  |  |  |  |  |  |
| Salviae Miltiorrhizae Radix et Rhizoma | MOL007069 | przewaquinone c | HSP90AA1 |  |  |  |  |  |  |
| Salviae Miltiorrhizae Radix et Rhizoma | MOL007069 | przewaquinone c | NCOA1 |  |  |  |  |  |  |
| Salviae Miltiorrhizae Radix et Rhizoma | MOL007070 | (6S,7R)-6,7-dihydroxy-1,6-dimethyl-8,9-dihydro-7H-naphtho[8,7-g]benzofuran-10,11-dione | HTR |  |  |  |  |  |  |
| Salviae Miltiorrhizae Radix et Rhizoma | MOL007070 | (6S,7R)-6,7-dihydroxy-1,6-dimethyl-8,9-dihydro-7H-naphtho[8,7-g]benzofuran-10,11-dione | PTGS2 |  |  |  |  |  |  |
| Salviae Miltiorrhizae Radix et Rhizoma | MOL007070 | (6S,7R)-6,7-dihydroxy-1,6-dimethyl-8,9-dihydro-7H-naphtho[8,7-g]benzofuran-10,11-dione | CA2 |  |  |  |  |  |  |
| Salviae Miltiorrhizae Radix et Rhizoma | MOL007070 | (6S,7R)-6,7-dihydroxy-1,6-dimethyl-8,9-dihydro-7H-naphtho[8,7-g]benzofuran-10,11-dione | ACHE |  |  |  |  |  |  |
| Salviae Miltiorrhizae Radix et Rhizoma | MOL007070 | (6S,7R)-6,7-dihydroxy-1,6-dimethyl-8,9-dihydro-7H-naphtho[8,7-g]benzofuran-10,11-dione | DPP4 |  |  |  |  |  |  |
| Salviae Miltiorrhizae Radix et Rhizoma | MOL007070 | (6S,7R)-6,7-dihydroxy-1,6-dimethyl-8,9-dihydro-7H-naphtho[8,7-g]benzofuran-10,11-dione | HSP90AA1 |  |  |  |  |  |  |
| Salviae Miltiorrhizae Radix et Rhizoma | MOL007070 | (6S,7R)-6,7-dihydroxy-1,6-dimethyl-8,9-dihydro-7H-naphtho[8,7-g]benzofuran-10,11-dione | PRSS1 |  |  |  |  |  |  |
| Salviae Miltiorrhizae Radix et Rhizoma | MOL007070 | (6S,7R)-6,7-dihydroxy-1,6-dimethyl-8,9-dihydro-7H-naphtho[8,7-g]benzofuran-10,11-dione | NCOA1 |  |  |  |  |  |  |
| Salviae Miltiorrhizae Radix et Rhizoma | MOL007071 | przewaquinone f | HTR |  |  |  |  |  |  |
| Salviae Miltiorrhizae Radix et Rhizoma | MOL007071 | przewaquinone f | PTGS2 |  |  |  |  |  |  |
| Salviae Miltiorrhizae Radix et Rhizoma | MOL007071 | przewaquinone f | DPP4 |  |  |  |  |  |  |
| Salviae Miltiorrhizae Radix et Rhizoma | MOL007071 | przewaquinone f | PRSS1 |  |  |  |  |  |  |
| Salviae Miltiorrhizae Radix et Rhizoma | MOL007071 | przewaquinone f | NCOA1 |  |  |  |  |  |  |
| Salviae Miltiorrhizae Radix et Rhizoma | MOL007077 | sclareol | PTGS2 |  |  |  |  |  |  |
| Salviae Miltiorrhizae Radix et Rhizoma | MOL007079 | tanshinaldehyde | DRD1 |  |  |  |  |  |  |
| Salviae Miltiorrhizae Radix et Rhizoma | MOL007079 | tanshinaldehyde | HTR |  |  |  |  |  |  |
| Salviae Miltiorrhizae Radix et Rhizoma | MOL007079 | tanshinaldehyde | CHRM1 |  |  |  |  |  |  |
| Salviae Miltiorrhizae Radix et Rhizoma | MOL007079 | tanshinaldehyde | PTGS2 |  |  |  |  |  |  |
| Salviae Miltiorrhizae Radix et Rhizoma | MOL007079 | tanshinaldehyde | OPRD1 |  |  |  |  |  |  |
| Salviae Miltiorrhizae Radix et Rhizoma | MOL007079 | tanshinaldehyde | ACHE |  |  |  |  |  |  |
| Salviae Miltiorrhizae Radix et Rhizoma | MOL007079 | tanshinaldehyde | ADRB2 |  |  |  |  |  |  |
| Salviae Miltiorrhizae Radix et Rhizoma | MOL007079 | tanshinaldehyde | OPRM1 |  |  |  |  |  |  |
| Salviae Miltiorrhizae Radix et Rhizoma | MOL007079 | tanshinaldehyde | DPP4 |  |  |  |  |  |  |
| Salviae Miltiorrhizae Radix et Rhizoma | MOL007079 | tanshinaldehyde | PRSS1 |  |  |  |  |  |  |
| Salviae Miltiorrhizae Radix et Rhizoma | MOL007079 | tanshinaldehyde | NCOA1 |  |  |  |  |  |  |
| Salviae Miltiorrhizae Radix et Rhizoma | MOL007081 | Salvia miltiorrhizaol B | PTGS2 |  |  |  |  |  |  |
| Salviae Miltiorrhizae Radix et Rhizoma | MOL007081 | Salvia miltiorrhizaol B | CA2 |  |  |  |  |  |  |
| Salviae Miltiorrhizae Radix et Rhizoma | MOL007081 | Salvia miltiorrhizaol B | PGR |  |  |  |  |  |  |
| Salviae Miltiorrhizae Radix et Rhizoma | MOL007081 | Salvia miltiorrhizaol B | OPRM1 |  |  |  |  |  |  |
| Salviae Miltiorrhizae Radix et Rhizoma | MOL007081 | Salvia miltiorrhizaol B | NR3C1 |  |  |  |  |  |  |
| Salviae Miltiorrhizae Radix et Rhizoma | MOL007081 | Salvia miltiorrhizaol B | HSP90AA1 |  |  |  |  |  |  |
| Salviae Miltiorrhizae Radix et Rhizoma | MOL007081 | Salvia miltiorrhizaol B | NCOA1 |  |  |  |  |  |  |
| Salviae Miltiorrhizae Radix et Rhizoma | MOL007082 | Salvia miltiorrhizaol A | PTGS1 |  |  |  |  |  |  |
| Salviae Miltiorrhizae Radix et Rhizoma | MOL007082 | Salvia miltiorrhizaol A | KCNH2 |  |  |  |  |  |  |
| Salviae Miltiorrhizae Radix et Rhizoma | MOL007082 | Salvia miltiorrhizaol A | SCN5A |  |  |  |  |  |  |
| Salviae Miltiorrhizae Radix et Rhizoma | MOL007082 | Salvia miltiorrhizaol A | PTGS2 |  |  |  |  |  |  |
| Salviae Miltiorrhizae Radix et Rhizoma | MOL007082 | Salvia miltiorrhizaol A | RXRA |  |  |  |  |  |  |
| Salviae Miltiorrhizae Radix et Rhizoma | MOL007082 | Salvia miltiorrhizaol A | NCOA1 |  |  |  |  |  |  |
| Salviae Miltiorrhizae Radix et Rhizoma | MOL007085 | Salvilenone | PTGS1 |  |  |  |  |  |  |
| Salviae Miltiorrhizae Radix et Rhizoma | MOL007085 | Salvilenone | ESR1 |  |  |  |  |  |  |
| Salviae Miltiorrhizae Radix et Rhizoma | MOL007085 | Salvilenone | AR |  |  |  |  |  |  |
| Salviae Miltiorrhizae Radix et Rhizoma | MOL007085 | Salvilenone | CHRM5 |  |  |  |  |  |  |
| Salviae Miltiorrhizae Radix et Rhizoma | MOL007085 | Salvilenone | PTGS2 |  |  |  |  |  |  |
| Salviae Miltiorrhizae Radix et Rhizoma | MOL007085 | Salvilenone | HTR3A |  |  |  |  |  |  |
| Salviae Miltiorrhizae Radix et Rhizoma | MOL007085 | Salvilenone | ESR2 |  |  |  |  |  |  |
| Salviae Miltiorrhizae Radix et Rhizoma | MOL007088 | cryptotanshinone | PTGS1 |  |  |  |  |  |  |
| Salviae Miltiorrhizae Radix et Rhizoma | MOL007088 | cryptotanshinone | DRD1 |  |  |  |  |  |  |
| Salviae Miltiorrhizae Radix et Rhizoma | MOL007088 | cryptotanshinone | CHRM3 |  |  |  |  |  |  |
| Salviae Miltiorrhizae Radix et Rhizoma | MOL007088 | cryptotanshinone | CHRM1 |  |  |  |  |  |  |
| Salviae Miltiorrhizae Radix et Rhizoma | MOL007088 | cryptotanshinone | SCN5A |  |  |  |  |  |  |
| Salviae Miltiorrhizae Radix et Rhizoma | MOL007088 | cryptotanshinone | CHRM5 |  |  |  |  |  |  |
| Salviae Miltiorrhizae Radix et Rhizoma | MOL007088 | cryptotanshinone | PTGS2 |  |  |  |  |  |  |
| Salviae Miltiorrhizae Radix et Rhizoma | MOL007088 | cryptotanshinone | CA2 |  |  |  |  |  |  |
| Salviae Miltiorrhizae Radix et Rhizoma | MOL007088 | cryptotanshinone | CHRM4 |  |  |  |  |  |  |
| Salviae Miltiorrhizae Radix et Rhizoma | MOL007088 | cryptotanshinone | OPRD1 |  |  |  |  |  |  |
| Salviae Miltiorrhizae Radix et Rhizoma | MOL007088 | cryptotanshinone | ADRA1A |  |  |  |  |  |  |
| Salviae Miltiorrhizae Radix et Rhizoma | MOL007088 | cryptotanshinone | CHRM2 |  |  |  |  |  |  |
| Salviae Miltiorrhizae Radix et Rhizoma | MOL007088 | cryptotanshinone | ADRA1B |  |  |  |  |  |  |
| Salviae Miltiorrhizae Radix et Rhizoma | MOL007088 | cryptotanshinone | ADRB2 |  |  |  |  |  |  |
| Salviae Miltiorrhizae Radix et Rhizoma | MOL007088 | cryptotanshinone | ADRA1D |  |  |  |  |  |  |
| Salviae Miltiorrhizae Radix et Rhizoma | MOL007088 | cryptotanshinone | OPRM1 |  |  |  |  |  |  |
| Salviae Miltiorrhizae Radix et Rhizoma | MOL007088 | cryptotanshinone | NCOA2 |  |  |  |  |  |  |
| Salviae Miltiorrhizae Radix et Rhizoma | MOL007088 | cryptotanshinone | NCOA1 |  |  |  |  |  |  |
| Salviae Miltiorrhizae Radix et Rhizoma | MOL007088 | cryptotanshinone | PGR |  |  |  |  |  |  |
| Salviae Miltiorrhizae Radix et Rhizoma | MOL007088 | cryptotanshinone | GABRA1 |  |  |  |  |  |  |
| Salviae Miltiorrhizae Radix et Rhizoma | MOL007088 | cryptotanshinone | RELA |  |  |  |  |  |  |
| Salviae Miltiorrhizae Radix et Rhizoma | MOL007088 | cryptotanshinone | STAT3 |  |  |  |  |  |  |
| Salviae Miltiorrhizae Radix et Rhizoma | MOL007088 | cryptotanshinone | CCND1 |  |  |  |  |  |  |
| Salviae Miltiorrhizae Radix et Rhizoma | MOL007088 | cryptotanshinone | BCL2L1 |  |  |  |  |  |  |
| Salviae Miltiorrhizae Radix et Rhizoma | MOL007088 | cryptotanshinone | TNFSF15 |  |  |  |  |  |  |
| Salviae Miltiorrhizae Radix et Rhizoma | MOL007088 | cryptotanshinone | APP |  |  |  |  |  |  |
| Salviae Miltiorrhizae Radix et Rhizoma | MOL007088 | cryptotanshinone | EDN3 |  |  |  |  |  |  |
| Salviae Miltiorrhizae Radix et Rhizoma | MOL007088 | cryptotanshinone | BIRC5 |  |  |  |  |  |  |
| Salviae Miltiorrhizae Radix et Rhizoma | MOL007093 | dan-shexinkum d | NOS2 |  |  |  |  |  |  |
| Salviae Miltiorrhizae Radix et Rhizoma | MOL007093 | dan-shexinkum d | PTGS1 |  |  |  |  |  |  |
| Salviae Miltiorrhizae Radix et Rhizoma | MOL007093 | dan-shexinkum d | HTR |  |  |  |  |  |  |
| Salviae Miltiorrhizae Radix et Rhizoma | MOL007093 | dan-shexinkum d | KCNH2 |  |  |  |  |  |  |
| Salviae Miltiorrhizae Radix et Rhizoma | MOL007093 | dan-shexinkum d | CHRM1 |  |  |  |  |  |  |
| Salviae Miltiorrhizae Radix et Rhizoma | MOL007093 | dan-shexinkum d | ESR1 |  |  |  |  |  |  |
| Salviae Miltiorrhizae Radix et Rhizoma | MOL007093 | dan-shexinkum d | AR |  |  |  |  |  |  |
| Salviae Miltiorrhizae Radix et Rhizoma | MOL007093 | dan-shexinkum d | SCN5A |  |  |  |  |  |  |
| Salviae Miltiorrhizae Radix et Rhizoma | MOL007093 | dan-shexinkum d | PPARG |  |  |  |  |  |  |
| Salviae Miltiorrhizae Radix et Rhizoma | MOL007093 | dan-shexinkum d | PTGS2 |  |  |  |  |  |  |
| Salviae Miltiorrhizae Radix et Rhizoma | MOL007093 | dan-shexinkum d | CA2 |  |  |  |  |  |  |
| Salviae Miltiorrhizae Radix et Rhizoma | MOL007093 | dan-shexinkum d | RXRA |  |  |  |  |  |  |
| Salviae Miltiorrhizae Radix et Rhizoma | MOL007093 | dan-shexinkum d | ACHE |  |  |  |  |  |  |
| Salviae Miltiorrhizae Radix et Rhizoma | MOL007093 | dan-shexinkum d | ADRA1B |  |  |  |  |  |  |
| Salviae Miltiorrhizae Radix et Rhizoma | MOL007093 | dan-shexinkum d | ADRB2 |  |  |  |  |  |  |
| Salviae Miltiorrhizae Radix et Rhizoma | MOL007093 | dan-shexinkum d | ESR2 |  |  |  |  |  |  |
| Salviae Miltiorrhizae Radix et Rhizoma | MOL007093 | dan-shexinkum d | DPP4 |  |  |  |  |  |  |
| Salviae Miltiorrhizae Radix et Rhizoma | MOL007093 | dan-shexinkum d | GSK3B |  |  |  |  |  |  |
| Salviae Miltiorrhizae Radix et Rhizoma | MOL007093 | dan-shexinkum d | CDK2 |  |  |  |  |  |  |
| Salviae Miltiorrhizae Radix et Rhizoma | MOL007093 | dan-shexinkum d | CHEK1 |  |  |  |  |  |  |
| Salviae Miltiorrhizae Radix et Rhizoma | MOL007093 | dan-shexinkum d | IGHG1 |  |  |  |  |  |  |
| Salviae Miltiorrhizae Radix et Rhizoma | MOL007093 | dan-shexinkum d | PRSS1 |  |  |  |  |  |  |
| Salviae Miltiorrhizae Radix et Rhizoma | MOL007093 | dan-shexinkum d | CCNA2 |  |  |  |  |  |  |
| Salviae Miltiorrhizae Radix et Rhizoma | MOL007093 | dan-shexinkum d | NCOA2 |  |  |  |  |  |  |
| Salviae Miltiorrhizae Radix et Rhizoma | MOL007093 | dan-shexinkum d | NCOA1 |  |  |  |  |  |  |
| Salviae Miltiorrhizae Radix et Rhizoma | MOL007093 | dan-shexinkum d | CALM1 |  |  |  |  |  |  |
| Salviae Miltiorrhizae Radix et Rhizoma | MOL007094 | Salvia miltiorrhizaspiroketallactone | PTGS1 |  |  |  |  |  |  |
| Salviae Miltiorrhizae Radix et Rhizoma | MOL007094 | Salvia miltiorrhizaspiroketallactone | DRD1 |  |  |  |  |  |  |
| Salviae Miltiorrhizae Radix et Rhizoma | MOL007094 | Salvia miltiorrhizaspiroketallactone | CHRM3 |  |  |  |  |  |  |
| Salviae Miltiorrhizae Radix et Rhizoma | MOL007094 | Salvia miltiorrhizaspiroketallactone | HTR |  |  |  |  |  |  |
| Salviae Miltiorrhizae Radix et Rhizoma | MOL007094 | Salvia miltiorrhizaspiroketallactone | CHRM1 |  |  |  |  |  |  |
| Salviae Miltiorrhizae Radix et Rhizoma | MOL007094 | Salvia miltiorrhizaspiroketallactone | ESR1 |  |  |  |  |  |  |
| Salviae Miltiorrhizae Radix et Rhizoma | MOL007094 | Salvia miltiorrhizaspiroketallactone | SCN5A |  |  |  |  |  |  |
| Salviae Miltiorrhizae Radix et Rhizoma | MOL007094 | Salvia miltiorrhizaspiroketallactone | CHRM5 |  |  |  |  |  |  |
| Salviae Miltiorrhizae Radix et Rhizoma | MOL007094 | Salvia miltiorrhizaspiroketallactone | PTGS2 |  |  |  |  |  |  |
| Salviae Miltiorrhizae Radix et Rhizoma | MOL007094 | Salvia miltiorrhizaspiroketallactone | CA2 |  |  |  |  |  |  |
| Salviae Miltiorrhizae Radix et Rhizoma | MOL007094 | Salvia miltiorrhizaspiroketallactone | CHRM4 |  |  |  |  |  |  |
| Salviae Miltiorrhizae Radix et Rhizoma | MOL007094 | Salvia miltiorrhizaspiroketallactone | RXRA |  |  |  |  |  |  |
| Salviae Miltiorrhizae Radix et Rhizoma | MOL007094 | Salvia miltiorrhizaspiroketallactone | ACHE |  |  |  |  |  |  |
| Salviae Miltiorrhizae Radix et Rhizoma | MOL007094 | Salvia miltiorrhizaspiroketallactone | ADRA1A |  |  |  |  |  |  |
| Salviae Miltiorrhizae Radix et Rhizoma | MOL007094 | Salvia miltiorrhizaspiroketallactone | CHRM2 |  |  |  |  |  |  |
| Salviae Miltiorrhizae Radix et Rhizoma | MOL007094 | Salvia miltiorrhizaspiroketallactone | ADRA1B |  |  |  |  |  |  |
| Salviae Miltiorrhizae Radix et Rhizoma | MOL007094 | Salvia miltiorrhizaspiroketallactone | ADRB2 |  |  |  |  |  |  |
| Salviae Miltiorrhizae Radix et Rhizoma | MOL007094 | Salvia miltiorrhizaspiroketallactone | ADRA1D |  |  |  |  |  |  |
| Salviae Miltiorrhizae Radix et Rhizoma | MOL007094 | Salvia miltiorrhizaspiroketallactone | CHRNA2 |  |  |  |  |  |  |
| Salviae Miltiorrhizae Radix et Rhizoma | MOL007094 | Salvia miltiorrhizaspiroketallactone | SLC6A4 |  |  |  |  |  |  |
| Salviae Miltiorrhizae Radix et Rhizoma | MOL007094 | Salvia miltiorrhizaspiroketallactone | OPRM1 |  |  |  |  |  |  |
| Salviae Miltiorrhizae Radix et Rhizoma | MOL007094 | Salvia miltiorrhizaspiroketallactone | GABRA1 |  |  |  |  |  |  |
| Salviae Miltiorrhizae Radix et Rhizoma | MOL007094 | Salvia miltiorrhizaspiroketallactone | DPP4 |  |  |  |  |  |  |
| Salviae Miltiorrhizae Radix et Rhizoma | MOL007094 | Salvia miltiorrhizaspiroketallactone | HSP90AA1 |  |  |  |  |  |  |
| Salviae Miltiorrhizae Radix et Rhizoma | MOL007098 | deoxyneocryptotanshinone | PTGS1 |  |  |  |  |  |  |
| Salviae Miltiorrhizae Radix et Rhizoma | MOL007098 | deoxyneocryptotanshinone | DRD1 |  |  |  |  |  |  |
| Salviae Miltiorrhizae Radix et Rhizoma | MOL007098 | deoxyneocryptotanshinone | CHRM3 |  |  |  |  |  |  |
| Salviae Miltiorrhizae Radix et Rhizoma | MOL007098 | deoxyneocryptotanshinone | CHRM1 |  |  |  |  |  |  |
| Salviae Miltiorrhizae Radix et Rhizoma | MOL007098 | deoxyneocryptotanshinone | ESR1 |  |  |  |  |  |  |
| Salviae Miltiorrhizae Radix et Rhizoma | MOL007098 | deoxyneocryptotanshinone | AR |  |  |  |  |  |  |
| Salviae Miltiorrhizae Radix et Rhizoma | MOL007098 | deoxyneocryptotanshinone | SCN5A |  |  |  |  |  |  |
| Salviae Miltiorrhizae Radix et Rhizoma | MOL007098 | deoxyneocryptotanshinone | CHRM5 |  |  |  |  |  |  |
| Salviae Miltiorrhizae Radix et Rhizoma | MOL007098 | deoxyneocryptotanshinone | PTGS2 |  |  |  |  |  |  |
| Salviae Miltiorrhizae Radix et Rhizoma | MOL007098 | deoxyneocryptotanshinone | CA2 |  |  |  |  |  |  |
| Salviae Miltiorrhizae Radix et Rhizoma | MOL007098 | deoxyneocryptotanshinone | CHRM4 |  |  |  |  |  |  |
| Salviae Miltiorrhizae Radix et Rhizoma | MOL007098 | deoxyneocryptotanshinone | RXRA |  |  |  |  |  |  |
| Salviae Miltiorrhizae Radix et Rhizoma | MOL007098 | deoxyneocryptotanshinone | OPRD1 |  |  |  |  |  |  |
| Salviae Miltiorrhizae Radix et Rhizoma | MOL007098 | deoxyneocryptotanshinone | ADRA1A |  |  |  |  |  |  |
| Salviae Miltiorrhizae Radix et Rhizoma | MOL007098 | deoxyneocryptotanshinone | CHRM2 |  |  |  |  |  |  |
| Salviae Miltiorrhizae Radix et Rhizoma | MOL007098 | deoxyneocryptotanshinone | ADRA1B |  |  |  |  |  |  |
| Salviae Miltiorrhizae Radix et Rhizoma | MOL007098 | deoxyneocryptotanshinone | ADRB2 |  |  |  |  |  |  |
| Salviae Miltiorrhizae Radix et Rhizoma | MOL007098 | deoxyneocryptotanshinone | ADRA1D |  |  |  |  |  |  |
| Salviae Miltiorrhizae Radix et Rhizoma | MOL007098 | deoxyneocryptotanshinone | OPRM1 |  |  |  |  |  |  |
| Salviae Miltiorrhizae Radix et Rhizoma | MOL007098 | deoxyneocryptotanshinone | GSK3B |  |  |  |  |  |  |
| Salviae Miltiorrhizae Radix et Rhizoma | MOL007098 | deoxyneocryptotanshinone | CDK2 |  |  |  |  |  |  |
| Salviae Miltiorrhizae Radix et Rhizoma | MOL007098 | deoxyneocryptotanshinone | IGHG1 |  |  |  |  |  |  |
| Salviae Miltiorrhizae Radix et Rhizoma | MOL007098 | deoxyneocryptotanshinone | NCOA2 |  |  |  |  |  |  |
| Salviae Miltiorrhizae Radix et Rhizoma | MOL007098 | deoxyneocryptotanshinone | NCOA1 |  |  |  |  |  |  |
| Salviae Miltiorrhizae Radix et Rhizoma | MOL007100 | dihydrotanshinlactone | NOS2 |  |  |  |  |  |  |
| Salviae Miltiorrhizae Radix et Rhizoma | MOL007100 | dihydrotanshinlactone | PTGS1 |  |  |  |  |  |  |
| Salviae Miltiorrhizae Radix et Rhizoma | MOL007100 | dihydrotanshinlactone | DRD1 |  |  |  |  |  |  |
| Salviae Miltiorrhizae Radix et Rhizoma | MOL007100 | dihydrotanshinlactone | CHRM3 |  |  |  |  |  |  |
| Salviae Miltiorrhizae Radix et Rhizoma | MOL007100 | dihydrotanshinlactone | HTR |  |  |  |  |  |  |
| Salviae Miltiorrhizae Radix et Rhizoma | MOL007100 | dihydrotanshinlactone | CHRM1 |  |  |  |  |  |  |
| Salviae Miltiorrhizae Radix et Rhizoma | MOL007100 | dihydrotanshinlactone | ESR1 |  |  |  |  |  |  |
| Salviae Miltiorrhizae Radix et Rhizoma | MOL007100 | dihydrotanshinlactone | AR |  |  |  |  |  |  |
| Salviae Miltiorrhizae Radix et Rhizoma | MOL007100 | dihydrotanshinlactone | SCN5A |  |  |  |  |  |  |
| Salviae Miltiorrhizae Radix et Rhizoma | MOL007100 | dihydrotanshinlactone | PPARG |  |  |  |  |  |  |
| Salviae Miltiorrhizae Radix et Rhizoma | MOL007100 | dihydrotanshinlactone | CHRM5 |  |  |  |  |  |  |
| Salviae Miltiorrhizae Radix et Rhizoma | MOL007100 | dihydrotanshinlactone | PTGS2 |  |  |  |  |  |  |
| Salviae Miltiorrhizae Radix et Rhizoma | MOL007100 | dihydrotanshinlactone | HTR3A |  |  |  |  |  |  |
| Salviae Miltiorrhizae Radix et Rhizoma | MOL007100 | dihydrotanshinlactone | CA2 |  |  |  |  |  |  |
| Salviae Miltiorrhizae Radix et Rhizoma | MOL007100 | dihydrotanshinlactone | RXRA |  |  |  |  |  |  |
| Salviae Miltiorrhizae Radix et Rhizoma | MOL007100 | dihydrotanshinlactone | ACHE |  |  |  |  |  |  |
| Salviae Miltiorrhizae Radix et Rhizoma | MOL007100 | dihydrotanshinlactone | ADRA1A |  |  |  |  |  |  |
| Salviae Miltiorrhizae Radix et Rhizoma | MOL007100 | dihydrotanshinlactone | ADRA1B |  |  |  |  |  |  |
| Salviae Miltiorrhizae Radix et Rhizoma | MOL007100 | dihydrotanshinlactone | SLC6A3 |  |  |  |  |  |  |
| Salviae Miltiorrhizae Radix et Rhizoma | MOL007100 | dihydrotanshinlactone | ADRB2 |  |  |  |  |  |  |
| Salviae Miltiorrhizae Radix et Rhizoma | MOL007100 | dihydrotanshinlactone | ADRA1D |  |  |  |  |  |  |
| Salviae Miltiorrhizae Radix et Rhizoma | MOL007100 | dihydrotanshinlactone | SLC6A4 |  |  |  |  |  |  |
| Salviae Miltiorrhizae Radix et Rhizoma | MOL007100 | dihydrotanshinlactone | OPRM1 |  |  |  |  |  |  |
| Salviae Miltiorrhizae Radix et Rhizoma | MOL007100 | dihydrotanshinlactone | GABRA1 |  |  |  |  |  |  |
| Salviae Miltiorrhizae Radix et Rhizoma | MOL007100 | dihydrotanshinlactone | DPP4 |  |  |  |  |  |  |
| Salviae Miltiorrhizae Radix et Rhizoma | MOL007100 | dihydrotanshinlactone | GSK3B |  |  |  |  |  |  |
| Salviae Miltiorrhizae Radix et Rhizoma | MOL007100 | dihydrotanshinlactone | IGHG1 |  |  |  |  |  |  |
| Salviae Miltiorrhizae Radix et Rhizoma | MOL007100 | dihydrotanshinlactone | PRSS1 |  |  |  |  |  |  |
| Salviae Miltiorrhizae Radix et Rhizoma | MOL007101 | dihydrotanshinoneⅠ | PTGS1 |  |  |  |  |  |  |
| Salviae Miltiorrhizae Radix et Rhizoma | MOL007101 | dihydrotanshinoneⅠ | SCN5A |  |  |  |  |  |  |
| Salviae Miltiorrhizae Radix et Rhizoma | MOL007101 | dihydrotanshinoneⅠ | PTGS2 |  |  |  |  |  |  |
| Salviae Miltiorrhizae Radix et Rhizoma | MOL007101 | dihydrotanshinoneⅠ | HTR3A |  |  |  |  |  |  |
| Salviae Miltiorrhizae Radix et Rhizoma | MOL007101 | dihydrotanshinoneⅠ | RXRA |  |  |  |  |  |  |
| Salviae Miltiorrhizae Radix et Rhizoma | MOL007101 | dihydrotanshinoneⅠ | ADRA1A |  |  |  |  |  |  |
| Salviae Miltiorrhizae Radix et Rhizoma | MOL007101 | dihydrotanshinoneⅠ | ADRA1B |  |  |  |  |  |  |
| Salviae Miltiorrhizae Radix et Rhizoma | MOL007101 | dihydrotanshinoneⅠ | ADRB2 |  |  |  |  |  |  |
| Salviae Miltiorrhizae Radix et Rhizoma | MOL007101 | dihydrotanshinoneⅠ | GABRA1 |  |  |  |  |  |  |
| Salviae Miltiorrhizae Radix et Rhizoma | MOL007101 | dihydrotanshinoneⅠ | HSP90AA1 |  |  |  |  |  |  |
| Salviae Miltiorrhizae Radix et Rhizoma | MOL007101 | dihydrotanshinoneⅠ | IGHG1 |  |  |  |  |  |  |
| Salviae Miltiorrhizae Radix et Rhizoma | MOL007101 | dihydrotanshinoneⅠ | NCOA2 |  |  |  |  |  |  |
| Salviae Miltiorrhizae Radix et Rhizoma | MOL007101 | dihydrotanshinoneⅠ | NCOA1 |  |  |  |  |  |  |
| Salviae Miltiorrhizae Radix et Rhizoma | MOL007101 | dihydrotanshinoneⅠ | CALM1 |  |  |  |  |  |  |
| Salviae Miltiorrhizae Radix et Rhizoma | MOL007105 | epiSalvia miltiorrhizaspiroketallactone | PTGS1 |  |  |  |  |  |  |
| Salviae Miltiorrhizae Radix et Rhizoma | MOL007105 | epiSalvia miltiorrhizaspiroketallactone | DRD1 |  |  |  |  |  |  |
| Salviae Miltiorrhizae Radix et Rhizoma | MOL007105 | epiSalvia miltiorrhizaspiroketallactone | CHRM3 |  |  |  |  |  |  |
| Salviae Miltiorrhizae Radix et Rhizoma | MOL007105 | epiSalvia miltiorrhizaspiroketallactone | CHRM1 |  |  |  |  |  |  |
| Salviae Miltiorrhizae Radix et Rhizoma | MOL007105 | epiSalvia miltiorrhizaspiroketallactone | ESR1 |  |  |  |  |  |  |
| Salviae Miltiorrhizae Radix et Rhizoma | MOL007105 | epiSalvia miltiorrhizaspiroketallactone | SCN5A |  |  |  |  |  |  |
| Salviae Miltiorrhizae Radix et Rhizoma | MOL007105 | epiSalvia miltiorrhizaspiroketallactone | CHRM5 |  |  |  |  |  |  |
| Salviae Miltiorrhizae Radix et Rhizoma | MOL007105 | epiSalvia miltiorrhizaspiroketallactone | PTGS2 |  |  |  |  |  |  |
| Salviae Miltiorrhizae Radix et Rhizoma | MOL007105 | epiSalvia miltiorrhizaspiroketallactone | CHRM4 |  |  |  |  |  |  |
| Salviae Miltiorrhizae Radix et Rhizoma | MOL007105 | epiSalvia miltiorrhizaspiroketallactone | RXRA |  |  |  |  |  |  |
| Salviae Miltiorrhizae Radix et Rhizoma | MOL007105 | epiSalvia miltiorrhizaspiroketallactone | OPRD1 |  |  |  |  |  |  |
| Salviae Miltiorrhizae Radix et Rhizoma | MOL007105 | epiSalvia miltiorrhizaspiroketallactone | ADRA1A |  |  |  |  |  |  |
| Salviae Miltiorrhizae Radix et Rhizoma | MOL007105 | epiSalvia miltiorrhizaspiroketallactone | CHRM2 |  |  |  |  |  |  |
| Salviae Miltiorrhizae Radix et Rhizoma | MOL007105 | epiSalvia miltiorrhizaspiroketallactone | ADRA1B |  |  |  |  |  |  |
| Salviae Miltiorrhizae Radix et Rhizoma | MOL007105 | epiSalvia miltiorrhizaspiroketallactone | ADRB2 |  |  |  |  |  |  |
| Salviae Miltiorrhizae Radix et Rhizoma | MOL007105 | epiSalvia miltiorrhizaspiroketallactone | ADRA1D |  |  |  |  |  |  |
| Salviae Miltiorrhizae Radix et Rhizoma | MOL007105 | epiSalvia miltiorrhizaspiroketallactone | SLC6A4 |  |  |  |  |  |  |
| Salviae Miltiorrhizae Radix et Rhizoma | MOL007105 | epiSalvia miltiorrhizaspiroketallactone | OPRM1 |  |  |  |  |  |  |
| Salviae Miltiorrhizae Radix et Rhizoma | MOL007105 | epiSalvia miltiorrhizaspiroketallactone | GABRA1 |  |  |  |  |  |  |
| Salviae Miltiorrhizae Radix et Rhizoma | MOL007105 | epiSalvia miltiorrhizaspiroketallactone | HSP90AA1 |  |  |  |  |  |  |
| Salviae Miltiorrhizae Radix et Rhizoma | MOL007105 | epiSalvia miltiorrhizaspiroketallactone | CDK2 |  |  |  |  |  |  |
| Salviae Miltiorrhizae Radix et Rhizoma | MOL007107 | C09092 | CHRM3 |  |  |  |  |  |  |
| Salviae Miltiorrhizae Radix et Rhizoma | MOL007107 | C09092 | HTR |  |  |  |  |  |  |
| Salviae Miltiorrhizae Radix et Rhizoma | MOL007107 | C09092 | CHRM1 |  |  |  |  |  |  |
| Salviae Miltiorrhizae Radix et Rhizoma | MOL007107 | C09092 | SCN5A |  |  |  |  |  |  |
| Salviae Miltiorrhizae Radix et Rhizoma | MOL007107 | C09092 | CA2 |  |  |  |  |  |  |
| Salviae Miltiorrhizae Radix et Rhizoma | MOL007107 | C09092 | ACHE |  |  |  |  |  |  |
| Salviae Miltiorrhizae Radix et Rhizoma | MOL007107 | C09092 | ADRA1A |  |  |  |  |  |  |
| Salviae Miltiorrhizae Radix et Rhizoma | MOL007107 | C09092 | CHRM2 |  |  |  |  |  |  |
| Salviae Miltiorrhizae Radix et Rhizoma | MOL007107 | C09092 | ADRA1B |  |  |  |  |  |  |
| Salviae Miltiorrhizae Radix et Rhizoma | MOL007107 | C09092 | ADRB2 |  |  |  |  |  |  |
| Salviae Miltiorrhizae Radix et Rhizoma | MOL007107 | C09092 | ADRA1D |  |  |  |  |  |  |
| Salviae Miltiorrhizae Radix et Rhizoma | MOL007107 | C09092 | OPRM1 |  |  |  |  |  |  |
| Salviae Miltiorrhizae Radix et Rhizoma | MOL007108 | isocryptotanshi-none | NOS2 |  |  |  |  |  |  |
| Salviae Miltiorrhizae Radix et Rhizoma | MOL007108 | isocryptotanshi-none | PTGS1 |  |  |  |  |  |  |
| Salviae Miltiorrhizae Radix et Rhizoma | MOL007108 | isocryptotanshi-none | DRD1 |  |  |  |  |  |  |
| Salviae Miltiorrhizae Radix et Rhizoma | MOL007108 | isocryptotanshi-none | CHRM3 |  |  |  |  |  |  |
| Salviae Miltiorrhizae Radix et Rhizoma | MOL007108 | isocryptotanshi-none | CHRM1 |  |  |  |  |  |  |
| Salviae Miltiorrhizae Radix et Rhizoma | MOL007108 | isocryptotanshi-none | ESR1 |  |  |  |  |  |  |
| Salviae Miltiorrhizae Radix et Rhizoma | MOL007108 | isocryptotanshi-none | AR |  |  |  |  |  |  |
| Salviae Miltiorrhizae Radix et Rhizoma | MOL007108 | isocryptotanshi-none | SCN5A |  |  |  |  |  |  |
| Salviae Miltiorrhizae Radix et Rhizoma | MOL007108 | isocryptotanshi-none | CHRM5 |  |  |  |  |  |  |
| Salviae Miltiorrhizae Radix et Rhizoma | MOL007108 | isocryptotanshi-none | PTGS2 |  |  |  |  |  |  |
| Salviae Miltiorrhizae Radix et Rhizoma | MOL007108 | isocryptotanshi-none | CA2 |  |  |  |  |  |  |
| Salviae Miltiorrhizae Radix et Rhizoma | MOL007108 | isocryptotanshi-none | CHRM4 |  |  |  |  |  |  |
| Salviae Miltiorrhizae Radix et Rhizoma | MOL007108 | isocryptotanshi-none | RXRA |  |  |  |  |  |  |
| Salviae Miltiorrhizae Radix et Rhizoma | MOL007108 | isocryptotanshi-none | OPRD1 |  |  |  |  |  |  |
| Salviae Miltiorrhizae Radix et Rhizoma | MOL007108 | isocryptotanshi-none | ACHE |  |  |  |  |  |  |
| Salviae Miltiorrhizae Radix et Rhizoma | MOL007108 | isocryptotanshi-none | ADRA1A |  |  |  |  |  |  |
| Salviae Miltiorrhizae Radix et Rhizoma | MOL007108 | isocryptotanshi-none | CHRM2 |  |  |  |  |  |  |
| Salviae Miltiorrhizae Radix et Rhizoma | MOL007108 | isocryptotanshi-none | ADRA1B |  |  |  |  |  |  |
| Salviae Miltiorrhizae Radix et Rhizoma | MOL007108 | isocryptotanshi-none | ADRB2 |  |  |  |  |  |  |
| Salviae Miltiorrhizae Radix et Rhizoma | MOL007108 | isocryptotanshi-none | ADRA1D |  |  |  |  |  |  |
| Salviae Miltiorrhizae Radix et Rhizoma | MOL007108 | isocryptotanshi-none | DRD2 |  |  |  |  |  |  |
| Salviae Miltiorrhizae Radix et Rhizoma | MOL007108 | isocryptotanshi-none | OPRM1 |  |  |  |  |  |  |
| Salviae Miltiorrhizae Radix et Rhizoma | MOL007108 | isocryptotanshi-none | GABRA1 |  |  |  |  |  |  |
| Salviae Miltiorrhizae Radix et Rhizoma | MOL007108 | isocryptotanshi-none | CDK2 |  |  |  |  |  |  |
| Salviae Miltiorrhizae Radix et Rhizoma | MOL007108 | isocryptotanshi-none | PRSS1 |  |  |  |  |  |  |
| Salviae Miltiorrhizae Radix et Rhizoma | MOL007108 | isocryptotanshi-none | NCOA2 |  |  |  |  |  |  |
| Salviae Miltiorrhizae Radix et Rhizoma | MOL007108 | isocryptotanshi-none | NCOA1 |  |  |  |  |  |  |
| Salviae Miltiorrhizae Radix et Rhizoma | MOL007111 | Isotanshinone II | NOS2 |  |  |  |  |  |  |
| Salviae Miltiorrhizae Radix et Rhizoma | MOL007111 | Isotanshinone II | DRD1 |  |  |  |  |  |  |
| Salviae Miltiorrhizae Radix et Rhizoma | MOL007111 | Isotanshinone II | CHRM3 |  |  |  |  |  |  |
| Salviae Miltiorrhizae Radix et Rhizoma | MOL007111 | Isotanshinone II | HTR |  |  |  |  |  |  |
| Salviae Miltiorrhizae Radix et Rhizoma | MOL007111 | Isotanshinone II | CHRM1 |  |  |  |  |  |  |
| Salviae Miltiorrhizae Radix et Rhizoma | MOL007111 | Isotanshinone II | ESR1 |  |  |  |  |  |  |
| Salviae Miltiorrhizae Radix et Rhizoma | MOL007111 | Isotanshinone II | AR |  |  |  |  |  |  |
| Salviae Miltiorrhizae Radix et Rhizoma | MOL007111 | Isotanshinone II | SCN5A |  |  |  |  |  |  |
| Salviae Miltiorrhizae Radix et Rhizoma | MOL007111 | Isotanshinone II | CHRM5 |  |  |  |  |  |  |
| Salviae Miltiorrhizae Radix et Rhizoma | MOL007111 | Isotanshinone II | PTGS2 |  |  |  |  |  |  |
| Salviae Miltiorrhizae Radix et Rhizoma | MOL007111 | Isotanshinone II | RXRA |  |  |  |  |  |  |
| Salviae Miltiorrhizae Radix et Rhizoma | MOL007111 | Isotanshinone II | OPRD1 |  |  |  |  |  |  |
| Salviae Miltiorrhizae Radix et Rhizoma | MOL007111 | Isotanshinone II | ACHE |  |  |  |  |  |  |
| Salviae Miltiorrhizae Radix et Rhizoma | MOL007111 | Isotanshinone II | ADRA1A |  |  |  |  |  |  |
| Salviae Miltiorrhizae Radix et Rhizoma | MOL007111 | Isotanshinone II | CHRM2 |  |  |  |  |  |  |
| Salviae Miltiorrhizae Radix et Rhizoma | MOL007111 | Isotanshinone II | ADRB2 |  |  |  |  |  |  |
| Salviae Miltiorrhizae Radix et Rhizoma | MOL007111 | Isotanshinone II | OPRM1 |  |  |  |  |  |  |
| Salviae Miltiorrhizae Radix et Rhizoma | MOL007111 | Isotanshinone II | ESR2 |  |  |  |  |  |  |
| Salviae Miltiorrhizae Radix et Rhizoma | MOL007111 | Isotanshinone II | GABRA1 |  |  |  |  |  |  |
| Salviae Miltiorrhizae Radix et Rhizoma | MOL007111 | Isotanshinone II | DPP4 |  |  |  |  |  |  |
| Salviae Miltiorrhizae Radix et Rhizoma | MOL007111 | Isotanshinone II | GSK3B |  |  |  |  |  |  |
| Salviae Miltiorrhizae Radix et Rhizoma | MOL007111 | Isotanshinone II | CDK2 |  |  |  |  |  |  |
| Salviae Miltiorrhizae Radix et Rhizoma | MOL007111 | Isotanshinone II | CHEK1 |  |  |  |  |  |  |
| Salviae Miltiorrhizae Radix et Rhizoma | MOL007111 | Isotanshinone II | CCNA2 |  |  |  |  |  |  |
| Salviae Miltiorrhizae Radix et Rhizoma | MOL007115 | manool | NCOA2 |  |  |  |  |  |  |
| Salviae Miltiorrhizae Radix et Rhizoma | MOL007119 | miltionone Ⅰ | PTGS1 |  |  |  |  |  |  |
| Salviae Miltiorrhizae Radix et Rhizoma | MOL007119 | miltionone Ⅰ | CHRM3 |  |  |  |  |  |  |
| Salviae Miltiorrhizae Radix et Rhizoma | MOL007119 | miltionone Ⅰ | CHRM1 |  |  |  |  |  |  |
| Salviae Miltiorrhizae Radix et Rhizoma | MOL007119 | miltionone Ⅰ | ESR1 |  |  |  |  |  |  |
| Salviae Miltiorrhizae Radix et Rhizoma | MOL007119 | miltionone Ⅰ | AR |  |  |  |  |  |  |
| Salviae Miltiorrhizae Radix et Rhizoma | MOL007119 | miltionone Ⅰ | SCN5A |  |  |  |  |  |  |
| Salviae Miltiorrhizae Radix et Rhizoma | MOL007119 | miltionone Ⅰ | PTGS2 |  |  |  |  |  |  |
| Salviae Miltiorrhizae Radix et Rhizoma | MOL007119 | miltionone Ⅰ | CA2 |  |  |  |  |  |  |
| Salviae Miltiorrhizae Radix et Rhizoma | MOL007119 | miltionone Ⅰ | RXRA |  |  |  |  |  |  |
| Salviae Miltiorrhizae Radix et Rhizoma | MOL007119 | miltionone Ⅰ | OPRD1 |  |  |  |  |  |  |
| Salviae Miltiorrhizae Radix et Rhizoma | MOL007119 | miltionone Ⅰ | ADRA1A |  |  |  |  |  |  |
| Salviae Miltiorrhizae Radix et Rhizoma | MOL007119 | miltionone Ⅰ | CHRM2 |  |  |  |  |  |  |
| Salviae Miltiorrhizae Radix et Rhizoma | MOL007119 | miltionone Ⅰ | ADRA1B |  |  |  |  |  |  |
| Salviae Miltiorrhizae Radix et Rhizoma | MOL007119 | miltionone Ⅰ | ADRB2 |  |  |  |  |  |  |
| Salviae Miltiorrhizae Radix et Rhizoma | MOL007119 | miltionone Ⅰ | OPRM1 |  |  |  |  |  |  |
| Salviae Miltiorrhizae Radix et Rhizoma | MOL007119 | miltionone Ⅰ | NR3C1 |  |  |  |  |  |  |
| Salviae Miltiorrhizae Radix et Rhizoma | MOL007119 | miltionone Ⅰ | GSK3B |  |  |  |  |  |  |
| Salviae Miltiorrhizae Radix et Rhizoma | MOL007119 | miltionone Ⅰ | CDK2 |  |  |  |  |  |  |
| Salviae Miltiorrhizae Radix et Rhizoma | MOL007119 | miltionone Ⅰ | IGHG1 |  |  |  |  |  |  |
| Salviae Miltiorrhizae Radix et Rhizoma | MOL007119 | miltionone Ⅰ | CCNA2 |  |  |  |  |  |  |
| Salviae Miltiorrhizae Radix et Rhizoma | MOL007119 | miltionone Ⅰ | NCOA2 |  |  |  |  |  |  |
| Salviae Miltiorrhizae Radix et Rhizoma | MOL007119 | miltionone Ⅰ | NCOA1 |  |  |  |  |  |  |
| Salviae Miltiorrhizae Radix et Rhizoma | MOL007120 | miltionone Ⅱ | HTR |  |  |  |  |  |  |
| Salviae Miltiorrhizae Radix et Rhizoma | MOL007120 | miltionone Ⅱ | PTGS2 |  |  |  |  |  |  |
| Salviae Miltiorrhizae Radix et Rhizoma | MOL007120 | miltionone Ⅱ | CA2 |  |  |  |  |  |  |
| Salviae Miltiorrhizae Radix et Rhizoma | MOL007120 | miltionone Ⅱ | ACHE |  |  |  |  |  |  |
| Salviae Miltiorrhizae Radix et Rhizoma | MOL007120 | miltionone Ⅱ | PGR |  |  |  |  |  |  |
| Salviae Miltiorrhizae Radix et Rhizoma | MOL007120 | miltionone Ⅱ | NR3C1 |  |  |  |  |  |  |
| Salviae Miltiorrhizae Radix et Rhizoma | MOL007120 | miltionone Ⅱ | NCOA2 |  |  |  |  |  |  |
| Salviae Miltiorrhizae Radix et Rhizoma | MOL007120 | miltionone Ⅱ | NCOA1 |  |  |  |  |  |  |
| Salviae Miltiorrhizae Radix et Rhizoma | MOL007121 | miltipolone | ESR1 |  |  |  |  |  |  |
| Salviae Miltiorrhizae Radix et Rhizoma | MOL007121 | miltipolone | ACHE |  |  |  |  |  |  |
| Salviae Miltiorrhizae Radix et Rhizoma | MOL007122 | Miltirone | PTGS1 |  |  |  |  |  |  |
| Salviae Miltiorrhizae Radix et Rhizoma | MOL007122 | Miltirone | DRD1 |  |  |  |  |  |  |
| Salviae Miltiorrhizae Radix et Rhizoma | MOL007122 | Miltirone | CHRM3 |  |  |  |  |  |  |
| Salviae Miltiorrhizae Radix et Rhizoma | MOL007122 | Miltirone | CHRM1 |  |  |  |  |  |  |
| Salviae Miltiorrhizae Radix et Rhizoma | MOL007122 | Miltirone | ESR1 |  |  |  |  |  |  |
| Salviae Miltiorrhizae Radix et Rhizoma | MOL007122 | Miltirone | AR |  |  |  |  |  |  |
| Salviae Miltiorrhizae Radix et Rhizoma | MOL007122 | Miltirone | DRD5 |  |  |  |  |  |  |
| Salviae Miltiorrhizae Radix et Rhizoma | MOL007122 | Miltirone | SCN5A |  |  |  |  |  |  |
| Salviae Miltiorrhizae Radix et Rhizoma | MOL007122 | Miltirone | CHRM5 |  |  |  |  |  |  |
| Salviae Miltiorrhizae Radix et Rhizoma | MOL007122 | Miltirone | PTGS2 |  |  |  |  |  |  |
| Salviae Miltiorrhizae Radix et Rhizoma | MOL007122 | Miltirone | CA2 |  |  |  |  |  |  |
| Salviae Miltiorrhizae Radix et Rhizoma | MOL007122 | Miltirone | ADRA2C |  |  |  |  |  |  |
| Salviae Miltiorrhizae Radix et Rhizoma | MOL007122 | Miltirone | CHRM4 |  |  |  |  |  |  |
| Salviae Miltiorrhizae Radix et Rhizoma | MOL007122 | Miltirone | RXRA |  |  |  |  |  |  |
| Salviae Miltiorrhizae Radix et Rhizoma | MOL007122 | Miltirone | OPRD1 |  |  |  |  |  |  |
| Salviae Miltiorrhizae Radix et Rhizoma | MOL007122 | Miltirone | ADRA1A |  |  |  |  |  |  |
| Salviae Miltiorrhizae Radix et Rhizoma | MOL007122 | Miltirone | CHRM2 |  |  |  |  |  |  |
| Salviae Miltiorrhizae Radix et Rhizoma | MOL007122 | Miltirone | ADRA1B |  |  |  |  |  |  |
| Salviae Miltiorrhizae Radix et Rhizoma | MOL007122 | Miltirone | SLC6A3 |  |  |  |  |  |  |
| Salviae Miltiorrhizae Radix et Rhizoma | MOL007122 | Miltirone | ADRB2 |  |  |  |  |  |  |
| Salviae Miltiorrhizae Radix et Rhizoma | MOL007122 | Miltirone | ADRA1D |  |  |  |  |  |  |
| Salviae Miltiorrhizae Radix et Rhizoma | MOL007122 | Miltirone | OPRM1 |  |  |  |  |  |  |
| Salviae Miltiorrhizae Radix et Rhizoma | MOL007122 | Miltirone | NCOA2 |  |  |  |  |  |  |
| Salviae Miltiorrhizae Radix et Rhizoma | MOL007124 | neocryptotanshinone ii | PTGS1 |  |  |  |  |  |  |
| Salviae Miltiorrhizae Radix et Rhizoma | MOL007124 | neocryptotanshinone ii | DRD1 |  |  |  |  |  |  |
| Salviae Miltiorrhizae Radix et Rhizoma | MOL007124 | neocryptotanshinone ii | CHRM3 |  |  |  |  |  |  |
| Salviae Miltiorrhizae Radix et Rhizoma | MOL007124 | neocryptotanshinone ii | CHRM1 |  |  |  |  |  |  |
| Salviae Miltiorrhizae Radix et Rhizoma | MOL007124 | neocryptotanshinone ii | ESR1 |  |  |  |  |  |  |
| Salviae Miltiorrhizae Radix et Rhizoma | MOL007124 | neocryptotanshinone ii | AR |  |  |  |  |  |  |
| Salviae Miltiorrhizae Radix et Rhizoma | MOL007124 | neocryptotanshinone ii | SCN5A |  |  |  |  |  |  |
| Salviae Miltiorrhizae Radix et Rhizoma | MOL007124 | neocryptotanshinone ii | PTGS2 |  |  |  |  |  |  |
| Salviae Miltiorrhizae Radix et Rhizoma | MOL007124 | neocryptotanshinone ii | CA2 |  |  |  |  |  |  |
| Salviae Miltiorrhizae Radix et Rhizoma | MOL007124 | neocryptotanshinone ii | CHRM4 |  |  |  |  |  |  |
| Salviae Miltiorrhizae Radix et Rhizoma | MOL007124 | neocryptotanshinone ii | RXRA |  |  |  |  |  |  |
| Salviae Miltiorrhizae Radix et Rhizoma | MOL007124 | neocryptotanshinone ii | OPRD1 |  |  |  |  |  |  |
| Salviae Miltiorrhizae Radix et Rhizoma | MOL007124 | neocryptotanshinone ii | ADRA1A |  |  |  |  |  |  |
| Salviae Miltiorrhizae Radix et Rhizoma | MOL007124 | neocryptotanshinone ii | CHRM2 |  |  |  |  |  |  |
| Salviae Miltiorrhizae Radix et Rhizoma | MOL007124 | neocryptotanshinone ii | ADRA1B |  |  |  |  |  |  |
| Salviae Miltiorrhizae Radix et Rhizoma | MOL007124 | neocryptotanshinone ii | SLC6A3 |  |  |  |  |  |  |
| Salviae Miltiorrhizae Radix et Rhizoma | MOL007124 | neocryptotanshinone ii | ADRB2 |  |  |  |  |  |  |
| Salviae Miltiorrhizae Radix et Rhizoma | MOL007124 | neocryptotanshinone ii | ADRA1D |  |  |  |  |  |  |
| Salviae Miltiorrhizae Radix et Rhizoma | MOL007124 | neocryptotanshinone ii | SLC6A4 |  |  |  |  |  |  |
| Salviae Miltiorrhizae Radix et Rhizoma | MOL007124 | neocryptotanshinone ii | OPRM1 |  |  |  |  |  |  |
| Salviae Miltiorrhizae Radix et Rhizoma | MOL007124 | neocryptotanshinone ii | GABRA1 |  |  |  |  |  |  |
| Salviae Miltiorrhizae Radix et Rhizoma | MOL007124 | neocryptotanshinone ii | GSK3B |  |  |  |  |  |  |
| Salviae Miltiorrhizae Radix et Rhizoma | MOL007124 | neocryptotanshinone ii | HSP90AA1 |  |  |  |  |  |  |
| Salviae Miltiorrhizae Radix et Rhizoma | MOL007124 | neocryptotanshinone ii | CDK2 |  |  |  |  |  |  |
| Salviae Miltiorrhizae Radix et Rhizoma | MOL007124 | neocryptotanshinone ii | CCNA2 |  |  |  |  |  |  |
| Salviae Miltiorrhizae Radix et Rhizoma | MOL007125 | neocryptotanshinone | PTGS1 |  |  |  |  |  |  |
| Salviae Miltiorrhizae Radix et Rhizoma | MOL007125 | neocryptotanshinone | CHRM3 |  |  |  |  |  |  |
| Salviae Miltiorrhizae Radix et Rhizoma | MOL007125 | neocryptotanshinone | CHRM1 |  |  |  |  |  |  |
| Salviae Miltiorrhizae Radix et Rhizoma | MOL007125 | neocryptotanshinone | SCN5A |  |  |  |  |  |  |
| Salviae Miltiorrhizae Radix et Rhizoma | MOL007125 | neocryptotanshinone | PPARG |  |  |  |  |  |  |
| Salviae Miltiorrhizae Radix et Rhizoma | MOL007125 | neocryptotanshinone | PTGS2 |  |  |  |  |  |  |
| Salviae Miltiorrhizae Radix et Rhizoma | MOL007125 | neocryptotanshinone | CA2 |  |  |  |  |  |  |
| Salviae Miltiorrhizae Radix et Rhizoma | MOL007125 | neocryptotanshinone | ADRA1B |  |  |  |  |  |  |
| Salviae Miltiorrhizae Radix et Rhizoma | MOL007125 | neocryptotanshinone | ADRB2 |  |  |  |  |  |  |
| Salviae Miltiorrhizae Radix et Rhizoma | MOL007125 | neocryptotanshinone | ADRA1D |  |  |  |  |  |  |
| Salviae Miltiorrhizae Radix et Rhizoma | MOL007125 | neocryptotanshinone | OPRM1 |  |  |  |  |  |  |
| Salviae Miltiorrhizae Radix et Rhizoma | MOL007125 | neocryptotanshinone | IGHG1 |  |  |  |  |  |  |
| Salviae Miltiorrhizae Radix et Rhizoma | MOL007125 | neocryptotanshinone | NCOA2 |  |  |  |  |  |  |
| Salviae Miltiorrhizae Radix et Rhizoma | MOL007125 | neocryptotanshinone | NCOA1 |  |  |  |  |  |  |
| Salviae Miltiorrhizae Radix et Rhizoma | MOL007127 | 1-methyl-8,9-dihydro-7H-naphtho[5,6-g]benzofuran-6,10,11-trione | PTGS1 |  |  |  |  |  |  |
| Salviae Miltiorrhizae Radix et Rhizoma | MOL007127 | 1-methyl-8,9-dihydro-7H-naphtho[5,6-g]benzofuran-6,10,11-trione | DRD1 |  |  |  |  |  |  |
| Salviae Miltiorrhizae Radix et Rhizoma | MOL007127 | 1-methyl-8,9-dihydro-7H-naphtho[5,6-g]benzofuran-6,10,11-trione | CHRM3 |  |  |  |  |  |  |
| Salviae Miltiorrhizae Radix et Rhizoma | MOL007127 | 1-methyl-8,9-dihydro-7H-naphtho[5,6-g]benzofuran-6,10,11-trione | HTR |  |  |  |  |  |  |
| Salviae Miltiorrhizae Radix et Rhizoma | MOL007127 | 1-methyl-8,9-dihydro-7H-naphtho[5,6-g]benzofuran-6,10,11-trione | SCN5A |  |  |  |  |  |  |
| Salviae Miltiorrhizae Radix et Rhizoma | MOL007127 | 1-methyl-8,9-dihydro-7H-naphtho[5,6-g]benzofuran-6,10,11-trione | CHRM5 |  |  |  |  |  |  |
| Salviae Miltiorrhizae Radix et Rhizoma | MOL007127 | 1-methyl-8,9-dihydro-7H-naphtho[5,6-g]benzofuran-6,10,11-trione | PTGS2 |  |  |  |  |  |  |
| Salviae Miltiorrhizae Radix et Rhizoma | MOL007127 | 1-methyl-8,9-dihydro-7H-naphtho[5,6-g]benzofuran-6,10,11-trione | CA2 |  |  |  |  |  |  |
| Salviae Miltiorrhizae Radix et Rhizoma | MOL007127 | 1-methyl-8,9-dihydro-7H-naphtho[5,6-g]benzofuran-6,10,11-trione | RXRA |  |  |  |  |  |  |
| Salviae Miltiorrhizae Radix et Rhizoma | MOL007127 | 1-methyl-8,9-dihydro-7H-naphtho[5,6-g]benzofuran-6,10,11-trione | ACHE |  |  |  |  |  |  |
| Salviae Miltiorrhizae Radix et Rhizoma | MOL007127 | 1-methyl-8,9-dihydro-7H-naphtho[5,6-g]benzofuran-6,10,11-trione | ADRA1A |  |  |  |  |  |  |
| Salviae Miltiorrhizae Radix et Rhizoma | MOL007127 | 1-methyl-8,9-dihydro-7H-naphtho[5,6-g]benzofuran-6,10,11-trione | ADRB2 |  |  |  |  |  |  |
| Salviae Miltiorrhizae Radix et Rhizoma | MOL007127 | 1-methyl-8,9-dihydro-7H-naphtho[5,6-g]benzofuran-6,10,11-trione | OPRM1 |  |  |  |  |  |  |
| Salviae Miltiorrhizae Radix et Rhizoma | MOL007127 | 1-methyl-8,9-dihydro-7H-naphtho[5,6-g]benzofuran-6,10,11-trione | GABRA1 |  |  |  |  |  |  |
| Salviae Miltiorrhizae Radix et Rhizoma | MOL007127 | 1-methyl-8,9-dihydro-7H-naphtho[5,6-g]benzofuran-6,10,11-trione | DPP4 |  |  |  |  |  |  |
| Salviae Miltiorrhizae Radix et Rhizoma | MOL007127 | 1-methyl-8,9-dihydro-7H-naphtho[5,6-g]benzofuran-6,10,11-trione | HSP90AA1 |  |  |  |  |  |  |
| Salviae Miltiorrhizae Radix et Rhizoma | MOL007127 | 1-methyl-8,9-dihydro-7H-naphtho[5,6-g]benzofuran-6,10,11-trione | IGHG1 |  |  |  |  |  |  |
| Salviae Miltiorrhizae Radix et Rhizoma | MOL007127 | 1-methyl-8,9-dihydro-7H-naphtho[5,6-g]benzofuran-6,10,11-trione | NCOA1 |  |  |  |  |  |  |
| Salviae Miltiorrhizae Radix et Rhizoma | MOL007130 | prolithospermic acid | NOS2 |  |  |  |  |  |  |
| Salviae Miltiorrhizae Radix et Rhizoma | MOL007130 | prolithospermic acid | PTGS1 |  |  |  |  |  |  |
| Salviae Miltiorrhizae Radix et Rhizoma | MOL007130 | prolithospermic acid | HTR |  |  |  |  |  |  |
| Salviae Miltiorrhizae Radix et Rhizoma | MOL007130 | prolithospermic acid | ESR1 |  |  |  |  |  |  |
| Salviae Miltiorrhizae Radix et Rhizoma | MOL007130 | prolithospermic acid | AR |  |  |  |  |  |  |
| Salviae Miltiorrhizae Radix et Rhizoma | MOL007130 | prolithospermic acid | PTGS2 |  |  |  |  |  |  |
| Salviae Miltiorrhizae Radix et Rhizoma | MOL007130 | prolithospermic acid | HSP90AA1 |  |  |  |  |  |  |
| Salviae Miltiorrhizae Radix et Rhizoma | MOL007130 | prolithospermic acid | PRSS1 |  |  |  |  |  |  |
| Salviae Miltiorrhizae Radix et Rhizoma | MOL007130 | prolithospermic acid | CALM1 |  |  |  |  |  |  |
| Salviae Miltiorrhizae Radix et Rhizoma | MOL007132 | (2R)-3-(3,4-dihydroxyphenyl)-2-[(Z)-3-(3,4-dihydroxyphenyl)acryloyl]oxy-propionic acid | HTR |  |  |  |  |  |  |
| Salviae Miltiorrhizae Radix et Rhizoma | MOL007132 | (2R)-3-(3,4-dihydroxyphenyl)-2-[(Z)-3-(3,4-dihydroxyphenyl)acryloyl]oxy-propionic acid | ESR1 |  |  |  |  |  |  |
| Salviae Miltiorrhizae Radix et Rhizoma | MOL007132 | (2R)-3-(3,4-dihydroxyphenyl)-2-[(Z)-3-(3,4-dihydroxyphenyl)acryloyl]oxy-propionic acid | AR |  |  |  |  |  |  |
| Salviae Miltiorrhizae Radix et Rhizoma | MOL007132 | (2R)-3-(3,4-dihydroxyphenyl)-2-[(Z)-3-(3,4-dihydroxyphenyl)acryloyl]oxy-propionic acid | PPARG |  |  |  |  |  |  |
| Salviae Miltiorrhizae Radix et Rhizoma | MOL007132 | (2R)-3-(3,4-dihydroxyphenyl)-2-[(Z)-3-(3,4-dihydroxyphenyl)acryloyl]oxy-propionic acid | PTGS2 |  |  |  |  |  |  |
| Salviae Miltiorrhizae Radix et Rhizoma | MOL007132 | (2R)-3-(3,4-dihydroxyphenyl)-2-[(Z)-3-(3,4-dihydroxyphenyl)acryloyl]oxy-propionic acid | DPP4 |  |  |  |  |  |  |
| Salviae Miltiorrhizae Radix et Rhizoma | MOL007132 | (2R)-3-(3,4-dihydroxyphenyl)-2-[(Z)-3-(3,4-dihydroxyphenyl)acryloyl]oxy-propionic acid | PRSS1 |  |  |  |  |  |  |
| Salviae Miltiorrhizae Radix et Rhizoma | MOL007132 | (2R)-3-(3,4-dihydroxyphenyl)-2-[(Z)-3-(3,4-dihydroxyphenyl)acryloyl]oxy-propionic acid | CCNA2 |  |  |  |  |  |  |
| Salviae Miltiorrhizae Radix et Rhizoma | MOL007141 | salvianolic acid g | PTGS2 |  |  |  |  |  |  |
| Salviae Miltiorrhizae Radix et Rhizoma | MOL007142 | salvianolic acid j | F7 |  |  |  |  |  |  |
| Salviae Miltiorrhizae Radix et Rhizoma | MOL007142 | salvianolic acid j | PRSS1 |  |  |  |  |  |  |
| Salviae Miltiorrhizae Radix et Rhizoma | MOL007143 | salvilenone Ⅰ | PTGS2 |  |  |  |  |  |  |
| Salviae Miltiorrhizae Radix et Rhizoma | MOL007143 | salvilenone Ⅰ | RXRA |  |  |  |  |  |  |
| Salviae Miltiorrhizae Radix et Rhizoma | MOL007143 | salvilenone Ⅰ | ACHE |  |  |  |  |  |  |
| Salviae Miltiorrhizae Radix et Rhizoma | MOL007143 | salvilenone Ⅰ | PGR |  |  |  |  |  |  |
| Salviae Miltiorrhizae Radix et Rhizoma | MOL007143 | salvilenone Ⅰ | NR3C1 |  |  |  |  |  |  |
| Salviae Miltiorrhizae Radix et Rhizoma | MOL007143 | salvilenone Ⅰ | NCOA2 |  |  |  |  |  |  |
| Salviae Miltiorrhizae Radix et Rhizoma | MOL007143 | salvilenone Ⅰ | NCOA1 |  |  |  |  |  |  |
| Salviae Miltiorrhizae Radix et Rhizoma | MOL007145 | salviolone | PTGS1 |  |  |  |  |  |  |
| Salviae Miltiorrhizae Radix et Rhizoma | MOL007145 | salviolone | DRD1 |  |  |  |  |  |  |
| Salviae Miltiorrhizae Radix et Rhizoma | MOL007145 | salviolone | CHRM3 |  |  |  |  |  |  |
| Salviae Miltiorrhizae Radix et Rhizoma | MOL007145 | salviolone | HTR |  |  |  |  |  |  |
| Salviae Miltiorrhizae Radix et Rhizoma | MOL007145 | salviolone | CHRM1 |  |  |  |  |  |  |
| Salviae Miltiorrhizae Radix et Rhizoma | MOL007145 | salviolone | DRD5 |  |  |  |  |  |  |
| Salviae Miltiorrhizae Radix et Rhizoma | MOL007145 | salviolone | SCN5A |  |  |  |  |  |  |
| Salviae Miltiorrhizae Radix et Rhizoma | MOL007145 | salviolone | CHRM5 |  |  |  |  |  |  |
| Salviae Miltiorrhizae Radix et Rhizoma | MOL007145 | salviolone | PTGS2 |  |  |  |  |  |  |
| Salviae Miltiorrhizae Radix et Rhizoma | MOL007145 | salviolone | ADRA2A |  |  |  |  |  |  |
| Salviae Miltiorrhizae Radix et Rhizoma | MOL007145 | salviolone | HTR3A |  |  |  |  |  |  |
| Salviae Miltiorrhizae Radix et Rhizoma | MOL007145 | salviolone | CHRM4 |  |  |  |  |  |  |
| Salviae Miltiorrhizae Radix et Rhizoma | MOL007145 | salviolone | OPRD1 |  |  |  |  |  |  |
| Salviae Miltiorrhizae Radix et Rhizoma | MOL007145 | salviolone | ACHE |  |  |  |  |  |  |
| Salviae Miltiorrhizae Radix et Rhizoma | MOL007145 | salviolone | SLC6A2 |  |  |  |  |  |  |
| Salviae Miltiorrhizae Radix et Rhizoma | MOL007145 | salviolone | ADRA1A |  |  |  |  |  |  |
| Salviae Miltiorrhizae Radix et Rhizoma | MOL007145 | salviolone | CHRM2 |  |  |  |  |  |  |
| Salviae Miltiorrhizae Radix et Rhizoma | MOL007145 | salviolone | ADRA2B |  |  |  |  |  |  |
| Salviae Miltiorrhizae Radix et Rhizoma | MOL007145 | salviolone | ADRA1B |  |  |  |  |  |  |
| Salviae Miltiorrhizae Radix et Rhizoma | MOL007145 | salviolone | SLC6A3 |  |  |  |  |  |  |
| Salviae Miltiorrhizae Radix et Rhizoma | MOL007145 | salviolone | ADRB2 |  |  |  |  |  |  |
| Salviae Miltiorrhizae Radix et Rhizoma | MOL007145 | salviolone | CHRNA2 |  |  |  |  |  |  |
| Salviae Miltiorrhizae Radix et Rhizoma | MOL007145 | salviolone | SLC6A4 |  |  |  |  |  |  |
| Salviae Miltiorrhizae Radix et Rhizoma | MOL007145 | salviolone | DRD2 |  |  |  |  |  |  |
| Salviae Miltiorrhizae Radix et Rhizoma | MOL007145 | salviolone | OPRM1 |  |  |  |  |  |  |
| Salviae Miltiorrhizae Radix et Rhizoma | MOL007145 | salviolone | GABRA1 |  |  |  |  |  |  |
| Salviae Miltiorrhizae Radix et Rhizoma | MOL007145 | salviolone | GABRG3 |  |  |  |  |  |  |
| Salviae Miltiorrhizae Radix et Rhizoma | MOL007145 | salviolone | GABRE |  |  |  |  |  |  |
| Salviae Miltiorrhizae Radix et Rhizoma | MOL007150 | (6S)-6-hydroxy-1-methyl-6-methylol-8,9-dihydro-7H-naphtho[8,7-g]benzofuran-10,11-quinone | HTR |  |  |  |  |  |  |
| Salviae Miltiorrhizae Radix et Rhizoma | MOL007150 | (6S)-6-hydroxy-1-methyl-6-methylol-8,9-dihydro-7H-naphtho[8,7-g]benzofuran-10,11-quinone | PTGS2 |  |  |  |  |  |  |
| Salviae Miltiorrhizae Radix et Rhizoma | MOL007150 | (6S)-6-hydroxy-1-methyl-6-methylol-8,9-dihydro-7H-naphtho[8,7-g]benzofuran-10,11-quinone | CA2 |  |  |  |  |  |  |
| Salviae Miltiorrhizae Radix et Rhizoma | MOL007150 | (6S)-6-hydroxy-1-methyl-6-methylol-8,9-dihydro-7H-naphtho[8,7-g]benzofuran-10,11-quinone | ACHE |  |  |  |  |  |  |
| Salviae Miltiorrhizae Radix et Rhizoma | MOL007150 | (6S)-6-hydroxy-1-methyl-6-methylol-8,9-dihydro-7H-naphtho[8,7-g]benzofuran-10,11-quinone | DPP4 |  |  |  |  |  |  |
| Salviae Miltiorrhizae Radix et Rhizoma | MOL007150 | (6S)-6-hydroxy-1-methyl-6-methylol-8,9-dihydro-7H-naphtho[8,7-g]benzofuran-10,11-quinone | HSP90AA1 |  |  |  |  |  |  |
| Salviae Miltiorrhizae Radix et Rhizoma | MOL007150 | (6S)-6-hydroxy-1-methyl-6-methylol-8,9-dihydro-7H-naphtho[8,7-g]benzofuran-10,11-quinone | PRSS1 |  |  |  |  |  |  |
| Salviae Miltiorrhizae Radix et Rhizoma | MOL007150 | (6S)-6-hydroxy-1-methyl-6-methylol-8,9-dihydro-7H-naphtho[8,7-g]benzofuran-10,11-quinone | NCOA1 |  |  |  |  |  |  |
| Salviae Miltiorrhizae Radix et Rhizoma | MOL007151 | Tanshindiol B | HTR |  |  |  |  |  |  |
| Salviae Miltiorrhizae Radix et Rhizoma | MOL007151 | Tanshindiol B | PTGS2 |  |  |  |  |  |  |
| Salviae Miltiorrhizae Radix et Rhizoma | MOL007151 | Tanshindiol B | CA2 |  |  |  |  |  |  |
| Salviae Miltiorrhizae Radix et Rhizoma | MOL007151 | Tanshindiol B | ACHE |  |  |  |  |  |  |
| Salviae Miltiorrhizae Radix et Rhizoma | MOL007151 | Tanshindiol B | DPP4 |  |  |  |  |  |  |
| Salviae Miltiorrhizae Radix et Rhizoma | MOL007151 | Tanshindiol B | HSP90AA1 |  |  |  |  |  |  |
| Salviae Miltiorrhizae Radix et Rhizoma | MOL007151 | Tanshindiol B | NCOA1 |  |  |  |  |  |  |
| Salviae Miltiorrhizae Radix et Rhizoma | MOL007152 | Przewaquinone E | HTR |  |  |  |  |  |  |
| Salviae Miltiorrhizae Radix et Rhizoma | MOL007152 | Przewaquinone E | PTGS2 |  |  |  |  |  |  |
| Salviae Miltiorrhizae Radix et Rhizoma | MOL007152 | Przewaquinone E | CA2 |  |  |  |  |  |  |
| Salviae Miltiorrhizae Radix et Rhizoma | MOL007152 | Przewaquinone E | ACHE |  |  |  |  |  |  |
| Salviae Miltiorrhizae Radix et Rhizoma | MOL007152 | Przewaquinone E | DPP4 |  |  |  |  |  |  |
| Salviae Miltiorrhizae Radix et Rhizoma | MOL007152 | Przewaquinone E | HSP90AA1 |  |  |  |  |  |  |
| Salviae Miltiorrhizae Radix et Rhizoma | MOL007152 | Przewaquinone E | NCOA1 |  |  |  |  |  |  |
| Salviae Miltiorrhizae Radix et Rhizoma | MOL007154 | tanshinone iia | DRD1 |  |  |  |  |  |  |
| Salviae Miltiorrhizae Radix et Rhizoma | MOL007154 | tanshinone iia | CHRM3 |  |  |  |  |  |  |
| Salviae Miltiorrhizae Radix et Rhizoma | MOL007154 | tanshinone iia | HTR |  |  |  |  |  |  |
| Salviae Miltiorrhizae Radix et Rhizoma | MOL007154 | tanshinone iia | CHRM1 |  |  |  |  |  |  |
| Salviae Miltiorrhizae Radix et Rhizoma | MOL007154 | tanshinone iia | SCN5A |  |  |  |  |  |  |
| Salviae Miltiorrhizae Radix et Rhizoma | MOL007154 | tanshinone iia | CHRM5 |  |  |  |  |  |  |
| Salviae Miltiorrhizae Radix et Rhizoma | MOL007154 | tanshinone iia | PTGS2 |  |  |  |  |  |  |
| Salviae Miltiorrhizae Radix et Rhizoma | MOL007154 | tanshinone iia | CHRM4 |  |  |  |  |  |  |
| Salviae Miltiorrhizae Radix et Rhizoma | MOL007154 | tanshinone iia | OPRD1 |  |  |  |  |  |  |
| Salviae Miltiorrhizae Radix et Rhizoma | MOL007154 | tanshinone iia | ACHE |  |  |  |  |  |  |
| Salviae Miltiorrhizae Radix et Rhizoma | MOL007154 | tanshinone iia | ADRA1A |  |  |  |  |  |  |
| Salviae Miltiorrhizae Radix et Rhizoma | MOL007154 | tanshinone iia | CHRM2 |  |  |  |  |  |  |
| Salviae Miltiorrhizae Radix et Rhizoma | MOL007154 | tanshinone iia | ADRB2 |  |  |  |  |  |  |
| Salviae Miltiorrhizae Radix et Rhizoma | MOL007154 | tanshinone iia | OPRM1 |  |  |  |  |  |  |
| Salviae Miltiorrhizae Radix et Rhizoma | MOL007154 | tanshinone iia | DPP4 |  |  |  |  |  |  |
| Salviae Miltiorrhizae Radix et Rhizoma | MOL007154 | tanshinone iia | NCOA1 |  |  |  |  |  |  |
| Salviae Miltiorrhizae Radix et Rhizoma | MOL007154 | tanshinone iia | RXRA |  |  |  |  |  |  |
| Salviae Miltiorrhizae Radix et Rhizoma | MOL007154 | tanshinone iia | RELA |  |  |  |  |  |  |
| Salviae Miltiorrhizae Radix et Rhizoma | MOL007154 | tanshinone iia | BCL2 |  |  |  |  |  |  |
| Salviae Miltiorrhizae Radix et Rhizoma | MOL007154 | tanshinone iia | FOS |  |  |  |  |  |  |
| Salviae Miltiorrhizae Radix et Rhizoma | MOL007154 | tanshinone iia | CDKN1A |  |  |  |  |  |  |
| Salviae Miltiorrhizae Radix et Rhizoma | MOL007154 | tanshinone iia | MMP9 |  |  |  |  |  |  |
| Salviae Miltiorrhizae Radix et Rhizoma | MOL007154 | tanshinone iia | JUN |  |  |  |  |  |  |
| Salviae Miltiorrhizae Radix et Rhizoma | MOL007154 | tanshinone iia | AHSA1 |  |  |  |  |  |  |
| Salviae Miltiorrhizae Radix et Rhizoma | MOL007154 | tanshinone iia | CASP3 |  |  |  |  |  |  |
| Salviae Miltiorrhizae Radix et Rhizoma | MOL007154 | tanshinone iia | TP63 |  |  |  |  |  |  |
| Salviae Miltiorrhizae Radix et Rhizoma | MOL007154 | tanshinone iia | NFKBIA |  |  |  |  |  |  |
| Salviae Miltiorrhizae Radix et Rhizoma | MOL007154 | tanshinone iia | FASN |  |  |  |  |  |  |
| Salviae Miltiorrhizae Radix et Rhizoma | MOL007154 | tanshinone iia | EDNRA |  |  |  |  |  |  |
| Salviae Miltiorrhizae Radix et Rhizoma | MOL007154 | tanshinone iia | EDN3 |  |  |  |  |  |  |
| Salviae Miltiorrhizae Radix et Rhizoma | MOL007154 | tanshinone iia | CYP3A4 |  |  |  |  |  |  |
| Salviae Miltiorrhizae Radix et Rhizoma | MOL007154 | tanshinone iia | CYP1A2 |  |  |  |  |  |  |
| Salviae Miltiorrhizae Radix et Rhizoma | MOL007154 | tanshinone iia | MYC |  |  |  |  |  |  |
| Salviae Miltiorrhizae Radix et Rhizoma | MOL007154 | tanshinone iia | CYP1A1 |  |  |  |  |  |  |
| Salviae Miltiorrhizae Radix et Rhizoma | MOL007154 | tanshinone iia | NR1I2 |  |  |  |  |  |  |
| Salviae Miltiorrhizae Radix et Rhizoma | MOL007154 | tanshinone iia | NPM1 |  |  |  |  |  |  |
| Salviae Miltiorrhizae Radix et Rhizoma | MOL007154 | tanshinone iia | ECE1 |  |  |  |  |  |  |
| Salviae Miltiorrhizae Radix et Rhizoma | MOL007154 | tanshinone iia | PARP4 |  |  |  |  |  |  |
| Salviae Miltiorrhizae Radix et Rhizoma | MOL007154 | tanshinone iia | CALCR |  |  |  |  |  |  |
| Salviae Miltiorrhizae Radix et Rhizoma | MOL007154 | tanshinone iia | ITGB3 |  |  |  |  |  |  |
| Salviae Miltiorrhizae Radix et Rhizoma | MOL007155 | (6S)-6-(hydroxymethyl)-1,6-dimethyl-8,9-dihydro-7H-naphtho[8,7-g]benzofuran-10,11-dione | HTR |  |  |  |  |  |  |
| Salviae Miltiorrhizae Radix et Rhizoma | MOL007155 | (6S)-6-(hydroxymethyl)-1,6-dimethyl-8,9-dihydro-7H-naphtho[8,7-g]benzofuran-10,11-dione | CHRM1 |  |  |  |  |  |  |
| Salviae Miltiorrhizae Radix et Rhizoma | MOL007155 | (6S)-6-(hydroxymethyl)-1,6-dimethyl-8,9-dihydro-7H-naphtho[8,7-g]benzofuran-10,11-dione | SCN5A |  |  |  |  |  |  |
| Salviae Miltiorrhizae Radix et Rhizoma | MOL007155 | (6S)-6-(hydroxymethyl)-1,6-dimethyl-8,9-dihydro-7H-naphtho[8,7-g]benzofuran-10,11-dione | PTGS2 |  |  |  |  |  |  |
| Salviae Miltiorrhizae Radix et Rhizoma | MOL007155 | (6S)-6-(hydroxymethyl)-1,6-dimethyl-8,9-dihydro-7H-naphtho[8,7-g]benzofuran-10,11-dione | OPRD1 |  |  |  |  |  |  |
| Salviae Miltiorrhizae Radix et Rhizoma | MOL007155 | (6S)-6-(hydroxymethyl)-1,6-dimethyl-8,9-dihydro-7H-naphtho[8,7-g]benzofuran-10,11-dione | ACHE |  |  |  |  |  |  |
| Salviae Miltiorrhizae Radix et Rhizoma | MOL007155 | (6S)-6-(hydroxymethyl)-1,6-dimethyl-8,9-dihydro-7H-naphtho[8,7-g]benzofuran-10,11-dione | ADRA1A |  |  |  |  |  |  |
| Salviae Miltiorrhizae Radix et Rhizoma | MOL007155 | (6S)-6-(hydroxymethyl)-1,6-dimethyl-8,9-dihydro-7H-naphtho[8,7-g]benzofuran-10,11-dione | ADRB2 |  |  |  |  |  |  |
| Salviae Miltiorrhizae Radix et Rhizoma | MOL007155 | (6S)-6-(hydroxymethyl)-1,6-dimethyl-8,9-dihydro-7H-naphtho[8,7-g]benzofuran-10,11-dione | OPRM1 |  |  |  |  |  |  |
| Salviae Miltiorrhizae Radix et Rhizoma | MOL007155 | (6S)-6-(hydroxymethyl)-1,6-dimethyl-8,9-dihydro-7H-naphtho[8,7-g]benzofuran-10,11-dione | DPP4 |  |  |  |  |  |  |
| Salviae Miltiorrhizae Radix et Rhizoma | MOL007155 | (6S)-6-(hydroxymethyl)-1,6-dimethyl-8,9-dihydro-7H-naphtho[8,7-g]benzofuran-10,11-dione | PRSS1 |  |  |  |  |  |  |
| Salviae Miltiorrhizae Radix et Rhizoma | MOL007155 | (6S)-6-(hydroxymethyl)-1,6-dimethyl-8,9-dihydro-7H-naphtho[8,7-g]benzofuran-10,11-dione | NCOA1 |  |  |  |  |  |  |
| Salviae Miltiorrhizae Radix et Rhizoma | MOL007156 | tanshinone Ⅵ | PTGS1 |  |  |  |  |  |  |
| Salviae Miltiorrhizae Radix et Rhizoma | MOL007156 | tanshinone Ⅵ | ESR1 |  |  |  |  |  |  |
| Salviae Miltiorrhizae Radix et Rhizoma | MOL007156 | tanshinone Ⅵ | AR |  |  |  |  |  |  |
| Salviae Miltiorrhizae Radix et Rhizoma | MOL007156 | tanshinone Ⅵ | SCN5A |  |  |  |  |  |  |
| Salviae Miltiorrhizae Radix et Rhizoma | MOL007156 | tanshinone Ⅵ | PPARG |  |  |  |  |  |  |
| Salviae Miltiorrhizae Radix et Rhizoma | MOL007156 | tanshinone Ⅵ | PTGS2 |  |  |  |  |  |  |
| Salviae Miltiorrhizae Radix et Rhizoma | MOL007156 | tanshinone Ⅵ | HSP90AA1 |  |  |  |  |  |  |
| Salviae Miltiorrhizae Radix et Rhizoma | MOL007156 | tanshinone Ⅵ | IGHG1 |  |  |  |  |  |  |
| Salviae Miltiorrhizae Radix et Rhizoma | MOL007156 | tanshinone Ⅵ | NCOA2 |  |  |  |  |  |  |
| Salviae Miltiorrhizae Radix et Rhizoma | MOL007156 | tanshinone Ⅵ | NCOA1 |  |  |  |  |  |  |
| Salviae Miltiorrhizae Radix et Rhizoma | MOL007156 | tanshinone Ⅵ | CALM1 |  |  |  |  |  |  |
| Santali Albi Lignum | MOL001755 | 24-Ethylcholest-4-en-3-one | PGR |  |  |  |  |  |  |
| Santali Albi Lignum | MOL001755 | 24-Ethylcholest-4-en-3-one | NR3C2 |  |  |  |  |  |  |
| Santali Albi Lignum | MOL001771 | poriferast-5-en-3beta-ol | PGR |  |  |  |  |  |  |
| Santali Albi Lignum | MOL001771 | poriferast-5-en-3beta-ol | NCOA2 |  |  |  |  |  |  |
| Santali Albi Lignum | MOL001973 | Sitosteryl acetate | PGR |  |  |  |  |  |  |
| Santali Albi Lignum | MOL000358 | beta-sitosterol | PGR |  |  |  |  |  |  |
| Santali Albi Lignum | MOL000358 | beta-sitosterol | NCOA2 |  |  |  |  |  |  |
| Santali Albi Lignum | MOL000358 | beta-sitosterol | PTGS1 |  |  |  |  |  |  |
| Santali Albi Lignum | MOL000358 | beta-sitosterol | PTGS2 |  |  |  |  |  |  |
| Santali Albi Lignum | MOL000358 | beta-sitosterol | HSP90AA1 |  |  |  |  |  |  |
| Santali Albi Lignum | MOL000358 | beta-sitosterol | KCNH2 |  |  |  |  |  |  |
| Santali Albi Lignum | MOL000358 | beta-sitosterol | DRD1 |  |  |  |  |  |  |
| Santali Albi Lignum | MOL000358 | beta-sitosterol | CHRM3 |  |  |  |  |  |  |
| Santali Albi Lignum | MOL000358 | beta-sitosterol | CHRM1 |  |  |  |  |  |  |
| Santali Albi Lignum | MOL000358 | beta-sitosterol | SCN5A |  |  |  |  |  |  |
| Santali Albi Lignum | MOL000358 | beta-sitosterol | CHRM4 |  |  |  |  |  |  |
| Santali Albi Lignum | MOL000358 | beta-sitosterol | ADRA1A |  |  |  |  |  |  |
| Santali Albi Lignum | MOL000358 | beta-sitosterol | CHRM2 |  |  |  |  |  |  |
| Santali Albi Lignum | MOL000358 | beta-sitosterol | ADRA1B |  |  |  |  |  |  |
| Santali Albi Lignum | MOL000358 | beta-sitosterol | ADRB2 |  |  |  |  |  |  |
| Santali Albi Lignum | MOL000358 | beta-sitosterol | CHRNA2 |  |  |  |  |  |  |
| Santali Albi Lignum | MOL000358 | beta-sitosterol | SLC6A4 |  |  |  |  |  |  |
| Santali Albi Lignum | MOL000358 | beta-sitosterol | OPRM1 |  |  |  |  |  |  |
| Santali Albi Lignum | MOL000358 | beta-sitosterol | GABRA1 |  |  |  |  |  |  |
| Santali Albi Lignum | MOL000358 | beta-sitosterol | BCL2 |  |  |  |  |  |  |
| Santali Albi Lignum | MOL000358 | beta-sitosterol | BAX |  |  |  |  |  |  |
| Santali Albi Lignum | MOL000358 | beta-sitosterol | CASP9 |  |  |  |  |  |  |
| Santali Albi Lignum | MOL000358 | beta-sitosterol | JUN |  |  |  |  |  |  |
| Santali Albi Lignum | MOL000358 | beta-sitosterol | CASP3 |  |  |  |  |  |  |
| Santali Albi Lignum | MOL000358 | beta-sitosterol | CASP8 |  |  |  |  |  |  |
| Santali Albi Lignum | MOL000358 | beta-sitosterol | PRKCA |  |  |  |  |  |  |
| Santali Albi Lignum | MOL000358 | beta-sitosterol | PON1 |  |  |  |  |  |  |
| Santali Albi Lignum | MOL000358 | beta-sitosterol | MAP2 |  |  |  |  |  |  |
| Santali Albi Lignum | MOL000449 | Stigmasterol | PGR |  |  |  |  |  |  |
| Santali Albi Lignum | MOL000449 | Stigmasterol | NR3C2 |  |  |  |  |  |  |
| Santali Albi Lignum | MOL000449 | Stigmasterol | NCOA2 |  |  |  |  |  |  |
| Santali Albi Lignum | MOL000449 | Stigmasterol | ADH1C |  |  |  |  |  |  |
| Santali Albi Lignum | MOL000449 | Stigmasterol | IGHG1 |  |  |  |  |  |  |
| Santali Albi Lignum | MOL000449 | Stigmasterol | RXRA |  |  |  |  |  |  |
| Santali Albi Lignum | MOL000449 | Stigmasterol | NCOA1 |  |  |  |  |  |  |
| Santali Albi Lignum | MOL000449 | Stigmasterol | PTGS1 |  |  |  |  |  |  |
| Santali Albi Lignum | MOL000449 | Stigmasterol | PTGS2 |  |  |  |  |  |  |
| Santali Albi Lignum | MOL000449 | Stigmasterol | ADRA2A |  |  |  |  |  |  |
| Santali Albi Lignum | MOL000449 | Stigmasterol | SLC6A2 |  |  |  |  |  |  |
| Santali Albi Lignum | MOL000449 | Stigmasterol | SLC6A3 |  |  |  |  |  |  |
| Santali Albi Lignum | MOL000449 | Stigmasterol | ADRB2 |  |  |  |  |  |  |
| Santali Albi Lignum | MOL000449 | Stigmasterol | AKR1B1 |  |  |  |  |  |  |
| Santali Albi Lignum | MOL000449 | Stigmasterol | PLAU |  |  |  |  |  |  |
| Santali Albi Lignum | MOL000449 | Stigmasterol | LTA4H |  |  |  |  |  |  |
| Santali Albi Lignum | MOL000449 | Stigmasterol | MAOB |  |  |  |  |  |  |
| Santali Albi Lignum | MOL000449 | Stigmasterol | MAOA |  |  |  |  |  |  |
| Santali Albi Lignum | MOL000449 | Stigmasterol | CTRB1 |  |  |  |  |  |  |
| Santali Albi Lignum | MOL000449 | Stigmasterol | CHRM3 |  |  |  |  |  |  |
| Santali Albi Lignum | MOL000449 | Stigmasterol | CHRM1 |  |  |  |  |  |  |
| Santali Albi Lignum | MOL000449 | Stigmasterol | ADRB1 |  |  |  |  |  |  |
| Santali Albi Lignum | MOL000449 | Stigmasterol | SCN5A |  |  |  |  |  |  |
| Santali Albi Lignum | MOL000449 | Stigmasterol | ADRA1A |  |  |  |  |  |  |
| Santali Albi Lignum | MOL000449 | Stigmasterol | CHRM2 |  |  |  |  |  |  |
| Santali Albi Lignum | MOL000449 | Stigmasterol | ADRA1B |  |  |  |  |  |  |
| Santali Albi Lignum | MOL000449 | Stigmasterol | GABRA1 |  |  |  |  |  |  |
| Santali Albi Lignum | MOL007514 | methyl icosa-11,14-dienoate | NCOA2 |  |  |  |  |  |  |
| Santali Albi Lignum | MOL007535 | (5S,8S,9S,10R,13R,14S,17R)-17-[(1R,4R)-4-ethyl-1,5-dimethylhexyl]-10,13-dimethyl-2,4,5,7,8,9,11,12,14,15,16,17-dodecahydro-1H-cyclopenta[a]phenanthrene-3,6-dione | PGR |  |  |  |  |  |  |
| Santali Albi Lignum | MOL007536 | Stigmasta-5,22-dien-3-beta-yl acetate | PGR |  |  |  |  |  |  |
| Amomi Fructus | MOL000354 | isorhamnetin | NOS2 |  |  |  |  |  |  |
| Amomi Fructus | MOL000354 | isorhamnetin | PTGS1 |  |  |  |  |  |  |
| Amomi Fructus | MOL000354 | isorhamnetin | ESR1 |  |  |  |  |  |  |
| Amomi Fructus | MOL000354 | isorhamnetin | AR |  |  |  |  |  |  |
| Amomi Fructus | MOL000354 | isorhamnetin | PPARG |  |  |  |  |  |  |
| Amomi Fructus | MOL000354 | isorhamnetin | PTGS2 |  |  |  |  |  |  |
| Amomi Fructus | MOL000354 | isorhamnetin | ESR2 |  |  |  |  |  |  |
| Amomi Fructus | MOL000354 | isorhamnetin | DPP4 |  |  |  |  |  |  |
| Amomi Fructus | MOL000354 | isorhamnetin | MAPK14 |  |  |  |  |  |  |
| Amomi Fructus | MOL000354 | isorhamnetin | GSK3B |  |  |  |  |  |  |
| Amomi Fructus | MOL000354 | isorhamnetin | HSP90AA1 |  |  |  |  |  |  |
| Amomi Fructus | MOL000354 | isorhamnetin | CDK2 |  |  |  |  |  |  |
| Amomi Fructus | MOL000354 | isorhamnetin | PRSS1 |  |  |  |  |  |  |
| Amomi Fructus | MOL000354 | isorhamnetin | CCNA2 |  |  |  |  |  |  |
| Amomi Fructus | MOL000354 | isorhamnetin | NCOA2 |  |  |  |  |  |  |
| Amomi Fructus | MOL000354 | isorhamnetin | CALM1 |  |  |  |  |  |  |
| Amomi Fructus | MOL000354 | isorhamnetin | PYGM |  |  |  |  |  |  |
| Amomi Fructus | MOL000354 | isorhamnetin | PPARD |  |  |  |  |  |  |
| Amomi Fructus | MOL000354 | isorhamnetin | CHEK1 |  |  |  |  |  |  |
| Amomi Fructus | MOL000354 | isorhamnetin | AKR1B1 |  |  |  |  |  |  |
| Amomi Fructus | MOL000354 | isorhamnetin | NCOA1 |  |  |  |  |  |  |
| Amomi Fructus | MOL000354 | isorhamnetin | F7 |  |  |  |  |  |  |
| Amomi Fructus | MOL000354 | isorhamnetin | HTR |  |  |  |  |  |  |
| Amomi Fructus | MOL000354 | isorhamnetin | ACHE |  |  |  |  |  |  |
| Amomi Fructus | MOL000354 | isorhamnetin | GABRA1 |  |  |  |  |  |  |
| Amomi Fructus | MOL000354 | isorhamnetin | MAOB |  |  |  |  |  |  |
| Amomi Fructus | MOL000354 | isorhamnetin | GRIA2 |  |  |  |  |  |  |
| Amomi Fructus | MOL000354 | isorhamnetin | RELA |  |  |  |  |  |  |
| Amomi Fructus | MOL000354 | isorhamnetin | NCF1 |  |  |  |  |  |  |
| Amomi Fructus | MOL000354 | isorhamnetin | OLR1 |  |  |  |  |  |  |
| Amomi Fructus | MOL000006 | luteolin | PTGS1 |  |  |  |  |  |  |
| Amomi Fructus | MOL000006 | luteolin | AR |  |  |  |  |  |  |
| Amomi Fructus | MOL000006 | luteolin | PTGS2 |  |  |  |  |  |  |
| Amomi Fructus | MOL000006 | luteolin | HSP90AA1 |  |  |  |  |  |  |
| Amomi Fructus | MOL000006 | luteolin | PRSS1 |  |  |  |  |  |  |
| Amomi Fructus | MOL000006 | luteolin | NCOA2 |  |  |  |  |  |  |
| Amomi Fructus | MOL000006 | luteolin | DPP4 |  |  |  |  |  |  |
| Amomi Fructus | MOL000006 | luteolin | RELA |  |  |  |  |  |  |
| Amomi Fructus | MOL000006 | luteolin | EGFR |  |  |  |  |  |  |
| Amomi Fructus | MOL000006 | luteolin | AKT1 |  |  |  |  |  |  |
| Amomi Fructus | MOL000006 | luteolin | VEGFA |  |  |  |  |  |  |
| Amomi Fructus | MOL000006 | luteolin | CCND1 |  |  |  |  |  |  |
| Amomi Fructus | MOL000006 | luteolin | BCL2L1 |  |  |  |  |  |  |
| Amomi Fructus | MOL000006 | luteolin | CDKN1A |  |  |  |  |  |  |
| Amomi Fructus | MOL000006 | luteolin | CASP9 |  |  |  |  |  |  |
| Amomi Fructus | MOL000006 | luteolin | MMP2 |  |  |  |  |  |  |
| Amomi Fructus | MOL000006 | luteolin | MMP9 |  |  |  |  |  |  |
| Amomi Fructus | MOL000006 | luteolin | MAPK1 |  |  |  |  |  |  |
| Amomi Fructus | MOL000006 | luteolin | IL10RB |  |  |  |  |  |  |
| Amomi Fructus | MOL000006 | luteolin | RB1 |  |  |  |  |  |  |
| Amomi Fructus | MOL000006 | luteolin | CDK4 |  |  |  |  |  |  |
| Amomi Fructus | MOL000006 | luteolin | TNFSF15 |  |  |  |  |  |  |
| Amomi Fructus | MOL000006 | luteolin | JUN |  |  |  |  |  |  |
| Amomi Fructus | MOL000006 | luteolin | IL6 |  |  |  |  |  |  |
| Amomi Fructus | MOL000006 | luteolin | CASP3 |  |  |  |  |  |  |
| Amomi Fructus | MOL000006 | luteolin | TP63 |  |  |  |  |  |  |
| Amomi Fructus | MOL000006 | luteolin | NFKBIA |  |  |  |  |  |  |
| Amomi Fructus | MOL000006 | luteolin | TOP1 |  |  |  |  |  |  |
| Amomi Fructus | MOL000006 | luteolin | MDM2 |  |  |  |  |  |  |
| Amomi Fructus | MOL000006 | luteolin | APP |  |  |  |  |  |  |
| Amomi Fructus | MOL000006 | luteolin | MMP1 |  |  |  |  |  |  |
| Amomi Fructus | MOL000006 | luteolin | PCNA |  |  |  |  |  |  |
| Amomi Fructus | MOL000006 | luteolin | ERBB2 |  |  |  |  |  |  |
| Amomi Fructus | MOL000006 | luteolin | PPARG |  |  |  |  |  |  |
| Amomi Fructus | MOL000006 | luteolin | HMOX1 |  |  |  |  |  |  |
| Amomi Fructus | MOL000006 | luteolin | CASP7 |  |  |  |  |  |  |
| Amomi Fructus | MOL000006 | luteolin | ICAM1 |  |  |  |  |  |  |
| Amomi Fructus | MOL000006 | luteolin | MCL1 |  |  |  |  |  |  |
| Amomi Fructus | MOL000006 | luteolin | BIRC5 |  |  |  |  |  |  |
| Amomi Fructus | MOL000006 | luteolin | IL2 |  |  |  |  |  |  |
| Amomi Fructus | MOL000006 | luteolin | CCNB1 |  |  |  |  |  |  |
| Amomi Fructus | MOL000006 | luteolin | TYR |  |  |  |  |  |  |
| Amomi Fructus | MOL000006 | luteolin | IFNGR1 |  |  |  |  |  |  |
| Amomi Fructus | MOL000006 | luteolin | IL4 |  |  |  |  |  |  |
| Amomi Fructus | MOL000006 | luteolin | TOP2A |  |  |  |  |  |  |
| Amomi Fructus | MOL000006 | luteolin | GSTP1 |  |  |  |  |  |  |
| Amomi Fructus | MOL000006 | luteolin | XIAP |  |  |  |  |  |  |
| Amomi Fructus | MOL000006 | luteolin | SLC2A4 |  |  |  |  |  |  |
| Amomi Fructus | MOL000006 | luteolin | INSRR |  |  |  |  |  |  |
| Amomi Fructus | MOL000006 | luteolin | CD40LG |  |  |  |  |  |  |
| Amomi Fructus | MOL000006 | luteolin | PTGES |  |  |  |  |  |  |
| Amomi Fructus | MOL000006 | luteolin | NUF2 |  |  |  |  |  |  |
| Amomi Fructus | MOL000006 | luteolin | ADCY2 |  |  |  |  |  |  |
| Amomi Fructus | MOL000006 | luteolin | MET |  |  |  |  |  |  |
| Amomi Fructus | MOL002322 | isovitexin | PTGS2 |  |  |  |  |  |  |
| Amomi Fructus | MOL002322 | isovitexin | AR |  |  |  |  |  |  |
| Amomi Fructus | MOL002322 | isovitexin | RELA |  |  |  |  |  |  |
| Amomi Fructus | MOL002322 | isovitexin | IKBKB |  |  |  |  |  |  |
| Amomi Fructus | MOL002322 | isovitexin | TNFSF15 |  |  |  |  |  |  |

| Table S1. ICM related genes |
| --- |
| suoX |
| ADRB1 |
| CYP2D6 |
| ADRB2 |
| ABCB1 |
| PTGS1 |
| PTGS2 |
| AKR1C1 |
| SLC22A6 |
| CYP2C19 |
| CYP2C9 |
| PRKAA1 |
| PRKAA2 |
| PRKAB1 |
| PRKAB2 |
| PRKAG1 |
| PRKAG2 |
| PRKAG3 |
| EDNRA |
| TP53 |
| HSPA5 |
| RPS6KA3 |
| NFKBIA |
| TNFAIP6 |
| CASP1 |
| CASP3 |
| IKBKB |
| MAPK1 |
| MAPK15 |
| MAPK3 |
| MAPK4 |
| MAPK6 |
| MAPK7 |
| CCND1 |
| MYC |
| PCNA |
| UGT1A6 |
| NAT2 |
| SLC22A8 |
| CA1 |
| CYP3A4 |
| CACNA1C |
| CYP1A1 |
| CYP2B6 |
| CACNA1B |
| CACNB1 |
| CACNA2D3 |
| CYP3A5 |
| CYP2C8 |
| SMPD1 |
| CACNA1I |
| ADRA1A |
| NDUFC2 |
| VEGFA |
| NPPB |
| GJA1 |
| KCNH2 |
| VCAM1 |
| XDH |
| CYP1A2 |
| CYP2E1 |
| ADRA1D |
| ADRA1B |
| ADRA2C |
| ADRA2B |
| ADRA2A |
| SELE |
| HIF1A |
| KCNJ4 |
| UGT1A1 |
| ALB |
| UGT2B4 |
| UGT2B7 |
| KCNJ2 |
| CACNG |
| CYP3A7 |
| ORM1 |
| ORM2 |
| ABCB5 |
| UGT1A9 |
| GUCY1A2 |
| PDE5A |
| PDE1B |
| PDE1A |
| CHRM1 |
| CHRM2 |
| CHRM3 |
| CHRM4 |
| CHRM5 |
| CACNA2D1 |
| CACNB2 |
| CACNA1D |
| CALM1 |
| SLCO1B1 |
| ABCB11 |
| CACNA1S |
| KCND3 |
| CACNA1G |
| CACNA1H |
| CYP2A6 |
| ABCC3 |
| ABCC2 |
| NR1I2 |
| KCNJ11 |
| CACNA1A |
| SLC6A4 |
| SLC22A1 |
| ABCC4 |
| SLC22A5 |
| SLCO1A2 |
| ABCC10 |
| SLC22A4 |
| CYP2C18 |
| ABCC1 |
| SLC47A1 |
| SLC47A2 |
| TTN |
| MYH7 |
| MYBPC3 |
| LMNA |
| FLNC |
| TNNT2 |
| MYH6 |
| TNNI3 |
| DSP |
| ACE |
| ACTC1 |
| RYR2 |
| DMD |
| TPM1 |
| DES |
| SCN5A |
| F2 |
| TNF |
| IL6 |
| NOS3 |
| DSG2 |
| TGFB3 |
| PKP2 |
| GLA |
| PSEN1 |
| AGTR1 |
| CRYAB |
| NPPA |
| LAMP2 |
| JUP |
| DSC2 |
| EMD |
| MYL2 |
| TTR |
| CSRP3 |
| PTPN11 |
| AGT |
| ELN |
| VCL |
| GATA4 |
| ACADVL |
| BAG3 |
| FLNC-AS1 |
| TGFB1 |
| KCNQ1 |
| MT-TL1 |
| ABCC9 |
| TGFB2 |
| PLN |
| FOS |
| MT-ND4 |
| PSEN2 |
| HADHA |
| MYL3 |
| PRDM16 |
| SYNE2 |
| ACTB |
| CRP |
| CPT2 |
| MT-CYB |
| FKRP |
| PPARG |
| LAMA4 |
| TCAP |
| KRAS |
| SYNE1 |
| MT-CO1 |
| NKX2-5 |
| FBN1 |
| MT-ATP6 |
| GJA5 |
| ACTG1 |
| MTHFR |
| IL1B |
| COL3A1 |
| BRAF |
| SOD2 |
| EDN1 |
| GNB3 |
| MT-TK |
| MYOZ2 |
| F5 |
| MMP9 |
| FXN |
| NFE2L2 |
| HRAS |
| CAV3 |
| ANKRD1 |
| GSN |
| MMP1 |
| APOE |
| POLG |
| VWF |
| CST3 |
| CALR |
| JAK2 |
| MYLK2 |
| MT-ND1 |
| LOX |
| SERPINE1 |
| ZMPSTE24 |
| MT-CO3 |
| GATA5 |
| TBX5 |
| SHOC2 |
| TPM3 |
| PIK3CA |
| PRKCH |
| HAND2 |
| COX14 |
| CTF1 |
| NDUFA1 |
| APOH |
| MRAS |
| HAMP |
| CAPN3 |
| SERPINC1 |
| REN |
| ADA2 |
| NRAS |
| SDHA |
| NOTCH1 |
| TBX20 |
| SERPINA3 |
| ACADS |
| MB |
| TLR4 |
| TREX1 |
| APOB |
| MYBPC1 |
| FHL2 |
| RAF1 |
| APP |
| BLOC1S6 |
| SELP |
| IGF1 |
| IL10 |
| NOTCH3 |
| MIR92B |
| MT-ND6 |
| CCL2 |
| ACTA2 |
| GATA6 |
| GNAS |
| SLC25A4 |
| NF1 |
| ESR1 |
| GATAD1 |
| PDGFRA |
| ACTA1 |
| MIR208A |
| CASP9 |
| CYCS |
| ICAM1 |
| CXCL8 |
| THBD |
| CD40LG |
| ADM |
| COX8A |
| PLA2G7 |
| SOD1 |
| CREB1 |
| KNG1 |
| CD36 |
| UQCRFS1 |
| NOS2 |
| MT-ND3 |
| ADIPOQ |
| OPA1 |
| MT-ND2 |
| AGL |
| IL4 |
| PYGM |
| MAPK14 |
| MT-ND5 |
| SLC2A10 |
| CASP2 |
| EDNRB |
| CAT |
| PKD2 |
| ADORA2A |
| TWNK |
| MYH11 |
| INS |
| FAS |
| BDNF |
| MMP2 |
| MMP3 |
| ANXA5 |
| FABP3 |
| IL17A |
| MYBPC2 |
| HSPA4 |
| HMOX1 |
| APOA1 |
| GSR |
| PPARA |
| TYR |
| GBE1 |
| RTN4 |
| SOX9 |
| PON1 |
| AKT1 |
| SLC8A1 |
| LAMA2 |
| F3 |
| CLCN1 |
| NCF1 |
| MAPT |
| BMP2 |
| IDH2 |
| COX4I1 |
| CASQ2 |
| HFE |
| RRAS |
| ITGB3 |
| FHL1 |
| DARS2 |
| BMP6 |
| NAGLU |
| TIMP1 |
| FGA |
| SPP1 |
| IL2 |
| CSF3 |
| HMGCL |
| MLYCD |
| CTNNB1 |
| IDUA |
| GUSB |
| SGCB |
| TNNI3K |
| HGF |
| SMAD3 |
| ENG |
| TGFBR1 |
| MIR146A |
| SMAD4 |
| TGFBR2 |
| KCNJ5 |
| CXCL12 |
| TH |
| MIAT |
| MMACHC |
| CYP11B2 |
| G6PD |
| SURF1 |
| TERT |
| TRPM4 |
| SRC |
| MT-CO2 |
| STAT3 |
| SLC25A3 |
| MALAT1 |
| TIMP3 |
| PPARGC1A |
| COL2A1 |
| LTBP4 |
| NAGA |
| MIR126 |
| B2M |
| ITGA2B |
| SIRT1 |
| MEN1 |
| HBB |
| GAPDH |
| ARSB |
| CP |
| ITGA2 |
| CXCR4 |
| SGSH |
| CAV1 |
| ECHS1 |
| ILK |
| IFNG |
| BCL2L1 |
| EPOR |
| CSF2 |
| PIK3C2A |
| HLA-DRB1 |
| F9 |
| OBSCN |
| HSPA8 |
| NR3C2 |
| PRKG1 |
| IDH1 |
| COL1A1 |
| HMGCR |
| FN1 |
| LEP |
| BRCA2 |
| ACAN |
| INSR |
| MPO |
| FGF2 |
| HSPA1A |
| CNR1 |
| SUN2 |
| COL4A1 |
| ITGA7 |
| MIR155 |
| IL13 |
| CD34 |
| AVP |
| PECAM1 |
| MAP2K1 |
| MYOCD |
| MMUT |
| HSPD1 |
| TNFRSF1A |
| CACNB4 |
| TRPV4 |
| NUP107 |
| ERCC6 |
| SELL |
| ARG1 |
| APOC3 |
| SULT1A3 |
| MIR21 |
| HJV |
| PRL |
| NPPC |
| NRAP |
| LBR |
| AQP4 |
| HBA1 |
| TIMP2 |
| IDS |
| FLT1 |
| IGF2 |
| GNPTAB |
| VKORC1 |
| TLR3 |
| JUN |
| PGF |
| ERCC4 |
| RYR1 |
| DENND11 |
| TFAM |
| CRYAA |
| H19 |
| APLN |
| AMPD1 |
| GPT |
| MIR199A1 |
| CDH5 |
| PON2 |
| PCCB |
| HGSNAT |
| SUN1 |
| NOS1 |
| EGFR |
| DRD2 |
| F13A1 |
| HADHB |
| IL18 |
| SON |
| CD68 |
| CCL3 |
| PLAT |
| PRDM10 |
| SOCS1 |
| TMEM126A |
| MFN2 |
| ZIC3 |
| IAPP |
| PDE3A |
| COG2 |
| CD2AP |
| ADAMTS13 |
| FGFR1 |
| IFNA1 |
| ANK2 |
| EPO |
| GBA |
| LMNB1 |
| APLNR |
| LPL |
| CDKN2B-AS1 |
| FHOD3 |
| AGTR2 |
| ENPP1 |
| MIR29A |
| F10 |
| PGM1 |
| TLR8 |
| LMNB2 |
| F2R |
| MIR145 |
| CD4 |
| MYOM3 |
| CPOX |
| ENTPD1 |
| HP |
| ARSA |
| MIR214 |
| ADRB3 |
| GSTM1 |
| HCN4 |
| HMOX2 |
| MIR499A |
| CX3CR1 |
| CTSD |
| SERPINF2 |
| PTPRC |
| THPO |
| LEMD2 |
| H2AC18 |
| ATP2A2 |
| P2RY12 |
| ECE1 |
| CD86 |
| OPA3 |
| SERPIND1 |
| TNFRSF11B |
| PRKD1 |
| WFS1 |
| FIG4 |
| RIT1 |
| LAMP1 |
| MAPK8 |
| HLA-B |
| ALAS1 |
| SMARCAL1 |
| CALCA |
| LCN2 |
| MIR210 |
| XPNPEP3 |
| EGF |
| JAG1 |
| ADORA1 |
| WDTC1 |
| NDUFS4 |
| MTOR |
| DLL1 |
| ERCC1 |
| ATP5F1E |
| ITGB1 |
| SI |
| CDH2 |
| LAMA1 |
| DNAH8 |
| PCCA |
| RCE1 |
| PFKM |
| RHOA |
| MIR196A2 |
| MMADHC |
| PDE4D |
| XIRP2 |
| HOPX |
| SNCA |
| LPA |
| CYBB |
| SYNE3 |
| COX5A |
| MLH1 |
| ANK3 |
| RETN |
| KCNK2 |
| CD8A |
| FIP1L1 |
| BRCC3 |
| CXCL1 |
| SCN1A |
| SCN3A |
| GCM2 |
| DSTYK |
| TLR2 |
| SDHB |
| MIR221 |
| KCNJ8 |
| VDAC1 |
| CCR5 |
| NPY |
| NSD1 |
| TMEM67 |
| MYMY4 |
| SYNGAP1 |
| FCGR3B |
| BLOC1S1 |
| FMR1 |
| HEY2 |
| ACTN4 |
| ALDH2 |
| ANK1 |
| SST |
| FTO |
| CLUAP1 |
| MIR17 |
| APOA2 |
| MAN2B1 |
| CDKN2A |
| CHIT1 |
| GGT1 |
| CS |
| M6PR |
| SAMHD1 |
| PF4 |
| SRRT |
| CISD2 |
| IL1RN |
| DRD1 |
| MYH9 |
| IL3 |
| GLB1 |
| CXADR |
| ITGB2 |
| LZTR1 |
| NPTX2 |
| GATA3 |
| CMYA5 |
| CTNNA1 |
| ODC1 |
| CD163 |
| PSAP |
| PIGA |
| PRKN |
| ACE2 |
| VIM |
| DUSP11 |
| IL5 |
| SQSTM1 |
| PKD1 |
| PRPS1 |
| IGF2R |
| HSD17B10 |
| HMGB1 |
| ATP2B3 |
| XIAP |
| KDR |
| CD19 |
| S100A12 |
| DLD |
| PINK1 |
| TMEM87B |
| CPT1A |
| COMT |
| CCL5 |
| HTR2A |
| MAP2 |
| CLU |
| PRKCE |
| KIT |
| CAMK2D |
| MT-ND4L |
| PDGFRB |
| NLRP3 |
| DNM1L |
| GALNS |
| DDX41 |
| VDR |
| COL4A2 |
| TXN |
| PIGT |
| CCR6 |
| MMD |
| CD40 |
| PTMS |
| ITGA4 |
| ACADL |
| ATP12A |
| ATP4A |
| PPBP |
| HTR3A |
| KARS1 |
| BSG |
| CRAT |
| PEX19 |
| LTA |
| ARSH |
| EYA1 |
| MIR140 |
| MYLK |
| LGALS3 |
| A4GALT |
| PRNP |
| GDF15 |
| S100B |
| TMLHE |
| C12orf65 |
| GHRL |
| NDUFA13 |
| KCNE2 |
| HLA-DQB1 |
| AIF1 |
| LRP5 |
| IFIH1 |
| ACTN1 |
| ATOH7 |
| CFLAR |
| WRN |
| OLR1 |
| ADORA3 |
| BGLAP |
| ANTXR1 |
| PPOX |
| CKM |
| CRH |
| NDUFA6 |
| STAT5B |
| MBP |
| SLC6A3 |
| GNS |
| CTLA4 |
| HSPB6 |
| BAX |
| TTPA |
| BMP7 |
| PTEN |
| TOMM20 |
| SMARCA4 |
| ADCY10 |
| TANGO2 |
| RPS6KA2 |
| PC |
| ADAM17 |
| PTH |
| MGP |
| ACADM |
| ITGAV |
| MGAM |
| APCS |
| ABCA1 |
| FURIN |
| SLC25A37 |
| NOX5 |
| OTC |
| HUWE1 |
| BANF1 |
| ERCC8 |
| LEMD3 |
| ICOSLG |
| SYNE4 |
| CBSL |
| MIR9-1 |
| GPX1 |
| SIRT3 |
| FUCA1 |
| ERC2 |
| BDKRB2 |
| GRIA3 |
| KCNA5 |
| CHGA |
| RPS6KA1 |
| RAN |
| DUOX1 |
| RNF144B |
| LEXM |
| ETFA |
| VIP |
| NEU1 |
| DBH |
| PSMB8 |
| ITGB6 |
| PTX3 |
| CCR2 |
| FBLN1 |
| TRIM63 |
| MYD88 |
| COQ8B |
| ATP2A3 |
| FKBP1A |
| IFNB1 |
| MIR222 |
| UGCG |
| CTSB |
| PES1 |
| SLC48A1 |
| FTH1 |
| CBL |
| MEF2C |
| ITGA1 |
| NPHS2 |
| SORT1 |
| ITGAM |
| MIR30A |
| FABP4 |
| CASP8 |
| LIPC |
| MIR22 |
| IMMT |
| POMC |
| AGER |
| IQCB1 |
| MIR223 |
| GATA1 |
| U2AF1 |
| IFNA2 |
| TGFA |
| TNFSF11 |
| TPI1 |
| BCL2 |
| RAC1 |
| EZH2 |
| ITGB5 |
| MEFV |
| SLC17A5 |
| KCNIP2 |
| PRKACA |
| CD63 |
| AQP1 |
| IGFBP3 |
| TNFRSF1B |
| MEF2A |
| WT1 |
| MIR142 |
| HECTD4 |
| TCF7L2 |
| HSPB7 |
| SPTB |
| NFATC1 |
| SMAD9 |
| ACADSB |
| ASL |
| SUMF1 |
| DR1 |
| AVIL |
| FAM107B |
| ITGA5 |
| MME |
| DCT |
| NRIP2 |
| MIR590 |
| AOC3 |
| TLR9 |
| CELF2 |
| UMOD |
| NR3C1 |
| ITGB1BP2 |
| MTPN |
| FCGR2A |
| PRTN3 |
| TF |
| RNASE3 |
| RPS27A |
| SLC2A4 |
| NUP153 |
| ATM |
| RUNX2 |
| RBP4 |
| ACHE |
| MICA |
| HDAC9 |
| FBLN5 |
| BTD |
| HIBCH |
| SLC4A5 |
| MED13L |
| FHL5 |
| MYOZ3 |
| AMPD3 |
| TBXA2R |
| FANCC |
| SMARCA2 |
| SPG7 |
| SMARCA1 |
| SRF |
| GRIN2B |
| ITPR1 |
| TLR7 |
| BRCA1 |
| EPHX2 |
| ITGA9 |
| PARP1 |
| CACNB3 |
| TNFRSF12A |
| IVD |
| TARDBP |
| ADA |
| AMBP |
| ADAM8 |
| OGA |
| H3-2 |
| VCP |
| S100A1 |
| CMA1 |
| HSD11B1 |
| DPP4 |
| MTR |
| CCN2 |
| ROCK1 |
| NPHS1 |
| COL7A1 |
| BECN1 |
| STAT5A |
| PTPN1 |
| SYNPO |
| PVALB |
| GZMB |
| GRIA1 |
| SLC9A1 |
| ACP5 |
| SLC45A2 |
| CAMK2G |
| HELLS |
| DKK1 |
| COL1A2 |
| CTSL |
| NCAM1 |
| PLA2G6 |
| NGF |
| PHACTR1 |
| DRD5 |
| ANKRD2 |
| OPRM1 |
| IL12B |
| XPA |
| NAGS |
| PRG2 |
| ERAS |
| GGT2 |
| GGTLC3 |
| MIR423 |
| CTNND1 |
| XBP1 |
| LEPR |
| MIR320A |
| GRIK1 |
| MMP7 |
| CPS1 |
| NEFH |
| ATP1A1 |
| BAD |
| CYP17A1 |
| CKB |
| ERBB2 |
| CASP7 |
| KCNN4 |
| IL11 |
| MMP8 |
| BLZF1 |
| OPTN |
| MIR30E |
| CR1 |
| ATP5F1A |
| CASR |
| ABCC6 |
| IL1A |
| CCK |
| APAF1 |
| MBL2 |
| ITGA6 |
| ABO |
| ISL1 |
| OSM |
| TLR5 |
| ANXA6 |
| TLN1 |
| HSPB8 |
| FRAS1 |
| ITGAL |
| XRCC4 |
| MIR361 |
| POSTN |
| CHI3L1 |
| PGAM2 |
| MIR483 |
| ITGB4 |
| NFATC4 |
| GH1 |
| FLNA |
| LMBRD1 |
| XRCC5 |
| ITGA8 |
| SLC2A6 |
| ASS1 |
| GLDC |
| BCKDK |
| MMAB |
| TP53BP1 |
| MCEE |
| BCKDHB |
| GCSH |
| HLCS |
| MYOC |
| MMAA |
| SLC25A46 |
| ZNF274 |
| GGACT |
| PPP1R3D |
| MEG3 |
| HTR1A |
| SERPINA1 |
| CCL11 |
| CD55 |
| ABL1 |
| TRPV2 |
| PPIG |
| ATP1A2 |
| MIR124-1 |
| PMM1 |
| NPR3 |
| MIR27A |
| PCSK9 |
| CCL26 |
| ACP1 |
| ANKRD23 |
| CHEK2 |
| AGA |
| FDPS |
| DRD3 |
| COL9A1 |
| MUS81 |
| NFE2 |
| FREM2 |
| HLTF |
| GAS5 |
| TLR6 |
| MIR132 |
| MUC1 |
| NAMPT |
| UCP2 |
| ENO3 |
| LRP1 |
| AIFM1 |
| GRM5 |
| CYP11B1 |
| AIP |
| GNE |
| ITGB8 |
| KLF15 |
| FASLG |
| IGFBP2 |
| IBSP |
| H2AX |
| GCG |
| TRAPPC9 |
| FTMT |
| CBX5 |
| GFER |
| SOST |
| CD69 |
| PDGFB |
| CYP2J2 |
| SLC1A1 |
| CHKA |
| LAMA3 |
| MSH6 |
| PRKDC |
| MSH2 |
| PMS2 |
| MAG |
| PFKP |
| AFG3L2 |
| ARHGEF1 |
| PFKL |
| UCP1 |
| EFEMP2 |
| SUV39H1 |
| FHL3 |
| AFF2 |
| SLC2A13 |
| USP6 |
| SLC2A11 |
| C19orf12 |
| SCAI |
| CFAP47 |
| IGF2-AS |
| MIR296 |
| C3 |
| SLC6A2 |
| IL2RA |
| ATG7 |
| C4A |
| STAT1 |
| APOA4 |
| TNFSF10 |
| TLR1 |
| NOX4 |
| CTSK |
| SSPN |
| IRS1 |
| TSPAN33 |
| CDH23 |
| PTHLH |
| FOXO1 |
| IL7R |
| ATG14 |
| MIR451A |
| BIN1 |
| SHBG |
| CCL17 |
| HSPB1 |
| TLR10 |
| DFFA |
| PEX2 |
| KL |
| WARS2 |
| HSD11B2 |
| SLC11A2 |
| TRPC3 |
| RHOD |
| GJA4 |
| CNBP |
| MC4R |
| CYSLTR1 |
| LAMB2 |
| FIS1 |
| PROC |
| TJP1 |
| MSTN |
| AKR1B1 |
| LEF1 |
| YY1 |
| ZFPM2 |
| FGF4 |
| VEGFC |
| CRLS1 |
| CASP6 |
| HTR1B |
| SIRT4 |
| ABCC8 |
| GABBR1 |
| HLA-A |
| FOXP3 |
| YWHAQ |
| CRHR1 |
| GAST |
| GJB2 |
| PARK7 |
| MMP14 |
| SIRT2 |
| TBX2 |
| GABBR2 |
| TAAR1 |
| ABCB7 |
| DBT |
| VEGFB |
| CASP10 |
| TRPM7 |
| DNM1 |
| BUB1B |
| HTR2C |
| SLC12A3 |
| PAX2 |
| MAF |
| RFC1 |
| COL5A1 |
| SIRT5 |
| SIRT6 |
| CLCNKB |
| SAG |
| PNMT |
| RBBP4 |
| POLL |
| HS3ST1 |
| GDAP1 |
| HPGDS |
| TBX1 |
| ATP1A4 |
| MRE11 |
| NCS1 |
| ICMT |
| SIRT7 |
| TWIST2 |
| SP7 |
| UBA7 |
| ADD2 |
| COTL1 |
| RECK |
| DGCR2 |
| NAT10 |
| GOLGB1 |
| GPS1 |
| PGGT1B |
| LIN9 |
| NSD2 |
| ZNF2 |
| PAGR1 |
| TSPAN16 |
| HOTAIR |
| MRGPRG-AS1 |
| MIR33A |
| LOC106050102 |
| LOC106050103 |
| EP300 |
| CASP4 |
| PDE4DIP |
| IGF1R |
| TEK |
| ATF2 |
| PDP1 |
| USP8 |
| CDKN3 |
| GAP43 |
| GPC3 |
| BGN |
| ADCYAP1 |
| DNM2 |
| SLC4A1 |
| MTTP |
| MIF |
| ANGPT2 |
| ACSS1 |
| APEX1 |
| ACSS2 |
| PPIC |
| PIK3CG |
| LIMK1 |
| MYL4 |
| TYMP |
| HPS1 |
| FYN |
| CKMT2 |
| GRK2 |
| ESR2 |
| MET |
| JPH3 |
| SGK1 |
| KLK1 |
| MAP2K7 |
| BDKRB1 |
| ITGA11 |
| TOMM40 |
| DMRTA1 |
| PAPPA |
| KITLG |
| CSF1 |
| TXN2 |
| TFRC |
| TNC |
| CBS |
| HAVCR1 |
| PLAU |
| TAC1 |
| OPRK1 |
| TCF21 |
| RUNX1 |
| P2RX7 |
| SOD3 |
| EDN2 |
| ERN1 |
| MYOD1 |
| DFFB |
| MAPK10 |
| CA2 |
| C5 |
| DLG4 |
| THBS1 |
| NPR1 |
| RARRES2 |
| ATXN1 |
| HAND1 |
| DECR1 |
| NOTCH2 |
| HSPA1B |
| SLC24A3 |
| SLC6A6 |
| CACNA1F |
| SELPLG |
| PRKCA |
| BLVRB |
| MIR130A |
| PITX2 |
| GCH1 |
| OGG1 |
| CDK7 |
| SLC2A1 |
| LY96 |
| KRT74 |
| HEY1 |
| ANG |
| NISCH |
| SDC1 |
| TRPV1 |
| TFB1M |
| MIR182 |
| MMP10 |
| OPRD1 |
| BMPR1A |
| CYBRD1 |
| PHYH |
| HSP90AA1 |
| KALRN |
| TBX3 |
| NFKB1 |
| CYC1 |
| CPT1B |
| MIR154 |
| IGFBP1 |
| SERPINA7 |
| P2RX4 |
| QDPR |
| SPRED1 |
| HSPB2 |
| VTN |
| PPP1CA |
| MAOA |
| TP63 |
| HTT |
| ITLN1 |
| NRG1 |
| HLA-DQA1 |
| MDM2 |
| TFG |
| NPR2 |
| PHB |
| STK11 |
| ADAMTSL1 |
| TYK2 |
| EIF2AK3 |
| MIR195 |
| NINJ2 |
| GCDH |
| GAMT |
| TIMM8A |
| PLSCR3 |
| BIRC5 |
| CXCL10 |
| AR |
| RELA |
| HLA-DRB4 |
| ATXN2 |
| EDN3 |
| FDFT1 |
| SYT9 |
| IL9 |
| PDE6B |
| CCR3 |
| PLAUR |
| MAP2K4 |
| UTS2 |
| CAMTA1 |
| BMPR2 |
| CEACAM5 |
| SLC8A3 |
| PYGB |
| MAPK9 |
| MCM10 |
| SUOX |
| GCK |
| CGAS |
| NTS |
| MIR143 |
| TWIST1 |
| MMP13 |
| MIR15A |
| MAP2K3 |
| EPRS1 |
| CASZ1 |
| HMCN1 |
| ITGA3 |
| GSTT1 |
| MRPL12 |
| PSMC5 |
| BMP4 |
| CALCR |
| EGR1 |
| PANK2 |
| LOC110806262 |
| CD44 |
| VHL |
| MIR122 |
| GHR |
| SEMA4A |
| KIF26B |
| G6PC |
| SREBF1 |
| NR1H2 |
| DDAH1 |
| S100A8 |
| APIP |
| WARS1 |
| LCK |
| SOCS3 |
| PDGFA |
| GRK5 |
| CIITA |
| GAL |
| GPX3 |
| FABP12 |
| JUND |
| ATP13A2 |
| IL1R1 |
| CD58 |
| TPO |
| LTF |
| YAP1 |
| CXCL16 |
| LMX1B |
| AHSP |
| MAP1LC3A |
| IKBKG |
| HSPA9 |
| PIM1 |
| AHSG |
| WDR83 |
| DUOX2 |
| ALOX12 |
| FGF23 |
| IL2RB |
| CLCN6 |
| GRIK2 |
| TRPC6 |
| NEDD4L |
| GHRH |
| TFPI |
| IL15 |
| KRT8 |
| HIGD1A |
| HLA-DRA |
| YWHAE |
| KCNN3 |
| PROS1 |
| ELAVL1 |
| ADAM12 |
| SLPI |
| EEF1A2 |
| PROM1 |
| BMX |
| GJC1 |
| ADCY1 |
| MIR24-1 |
| NF2 |
| GRIA2 |
| FADD |
| ITGB7 |
| ITGA10 |
| CBR1 |
| DGKB |
| TMCO6 |
| CFH |
| NT5E |
| SP1 |
| LIF |
| DLK1 |
| RGS19 |
| P4HB |
| PDIA2 |
| ETV7 |
| EPHB2 |
| DCN |
| TCF7 |
| PLIN1 |
| PCNT |
| MMD2 |
| IFNAR1 |
| ITIH4 |
| ATF6 |
| CXCR3 |
| PEPD |
| COL14A1 |
| MIR34A |
| SYT11 |
| WNT16 |
| CLK2 |
| MEX3A |
| KHDC4 |
| ENSG00000257062 |
| lnc-MEX3A-1 |
| lnc-RAB25-1 |
| RF00017-363 |
| lnc-UBQLN4-1 |
| piR-38580-023 |
| GLUD1 |
| TAT |
| DIABLO |
| SLC8A2 |
| DYNLL1 |
| CES1 |
| GARS1 |
| MX1 |
| NPL |
| TKT |
| RBM8A |
| GATA2 |
| MIR199B |
| MIR20A |
| ALDH1L1 |
| GNAI1 |
| CYP19A1 |
| IL21 |
| CDH13 |
| SCARB2 |
| TEAD1 |
| FGF14 |
| HSPE1 |
| CLDN5 |
| CDKN1A |
| IL4R |
| ALOX15 |
| IL6R |
| ITPR2 |
| MIR125A |
| MASP2 |
| CIB1 |
| ST2 |
| WWTR1 |
| CSF3R |
| EPHA3 |
| IL27 |
| MITF |
| ALDH1L1-AS2 |
| ITM2B |
| CAPN2 |
| IL6ST |
| ABCG2 |
| DVL1 |
| MAP3K12 |
| ADH1B |
| ABCG8 |
| COMP |
| HPR |
| CFTR |
| ADAMTS4 |
| NPC2 |
| FGF21 |
| OSMR |
| GAB1 |
| HBG2 |
| MCL1 |
| TJP2 |
| NDUFS5 |
| RNF126 |
| BCHE |
| SCN10A |
| S1PR1 |
| PAFAH1B1 |
| MIR185 |
| DNMT3A |
| PRKAR1A |
| MVK |
| FST |
| MIR335 |
| IL23R |
| TRAF6 |
| TERC |
| RPS6KB1 |
| EPHA4 |
| AFP |
| MIR17HG |
| CXCL9 |
| NLRP1 |
| MFN1 |
| GJB1 |
| SLC5A2 |
| PDE4A |
| MAP3K5 |
| PRKCD |
| EMX2OS |
| COL6A2 |
| TNFAIP3 |
| SLC5A1 |
| NAALADL2 |
| RB1 |
| TACR1 |
| EGFL7 |
| MIR495 |
| IL1RL1 |
| CREBBP |
| NTRK1 |
| UBR3 |
| TRPC1 |
| HSF1 |
| SERPINA4 |
| PTK2 |
| ATP1A3 |
| LINC00336 |
| TNFRSF11A |
| TSPAN2 |
| VCAN |
| ZNF335 |
| MTERF1 |
| MIR382 |
| RENBP |
| IRAK4 |
| CUL3 |
| GLO1 |
| PCSK6 |
| RBM3 |
| MIR486-1 |
| SLC19A1 |
| S100A9 |
| ENPEP |
| MUC16 |
| ABCB8 |
| TRH |
| AGPAT1 |
| MIR27B |
| EIF4A2 |
| NAB2 |
| RNASE2 |
| MIR134 |
| MIR30D |
| BCL2L11 |
| UCN |
| CAV2 |
| FCN1 |
| PDE11A |
| PRKCB |
| INHBA |
| CCNH |
| CDK1 |
| MAOB |
| MEIS1 |
| ADAMTS8 |
| PLD2 |
| UTS2R |
| PTPA |
| RGS2 |
| IL1RAPL2 |
| STK38 |
| IRAK1 |
| REST |
| RHAG |
| FADS2 |
| ATP2B1 |
| LAMB1 |
| ADPRH |
| PDLIM1 |
| CCL23 |
| HSPG2 |
| CORIN |
| HDAC1 |
| CTSG |
| STC1 |
| COX5B |
| PITRM1 |
| MIR93 |
| TCF4 |
| PPARD |
| ALPL |
| ICAM4 |
| ABCF2 |
| ESRRB |
| SOX2 |
| RO60 |
| MIR25 |
| PTPN22 |
| BRAP |
| HBEGF |
| GNAQ |
| CDK6 |
| WEE1 |
| STT3B |
| FUT8 |
| ATP6V1G3 |
| SBF2 |
| HPS4 |
| RPH3A |
| PHETA1 |
| DCTN1 |
| TGFBI |
| AGRN |
| DDIT3 |
| RCAN1 |
| IL33 |
| XRCC1 |
| TNFSF12 |
| HDAC4 |
| MYL1 |
| KRT18 |
| KCP |
| GGCX |
| MUTYH |
| NMT1 |
| CPQ |
| PCDHGA3 |
| JUNB |
| FNDC5 |
| DUSP19 |
| ENO1 |
| GSK3B |
| TTF2 |
| AP3B1 |
| SNX19 |
| MIR16-1 |
| STAT4 |
| THBS4 |
| HRH2 |
| GRM2 |
| VIPR1 |
| APOD |
| FOXO3 |
| SPTAN1 |
| SMAD1 |
| FBLN2 |
| PON3 |
| MIRLET7I |
| KLF4 |
| ALDH9A1 |
| INTS5 |
| MIR487B |
| KEAP1 |
| SMAD2 |
| ARNTL |
| LPIN1 |
| STUB1 |
| ROR1 |
| HNF1A |
| TIMP4 |
| HLA-G |
| ASIC2 |
| AKT2 |
| NPC1 |
| HAS2 |
| AP4M1 |
| AKAP7 |
| NPM1 |
| DKC1 |
| RTEL1 |
| CD79A |
| SUMO1 |
| TGM1 |
| CUBN |
| ADAM10 |
| ACVR1 |
| PRKCZ |
| SHC1 |
| TPP1 |
| GNRH1 |
| RHD |
| PTCD1 |
| MIR30B |
| MIR29C |
| MIR376C |
| IRF1 |
| NGFR |
| LEPQTL1 |
| FABP1 |
| ESRRG |
| EIF1AY |
| SLC11A1 |
| PROK2 |
| AARS1 |
| SIAH2 |
| MKI67 |
| DNASE1 |
| ALDOA |
| IRX4 |
| TRAF3 |
| PRDX2 |
| GRK4 |
| ATP6V1E1 |
| MKKS |
| TFB2M |
| PTGDS |
| RGS4 |
| CCR1 |
| PRPF8 |
| PPP1CC |
| SLC7A1 |
| KLHL3 |
| YWHAZ |
| NR1H3 |
| VASP |
| HCK |
| ADD3 |
| DUSP13 |
| AGTRAP |
| CD52 |
| MIR139 |
| MIR133A1 |
| FLG |
| APRT |
| CARS1 |
| PTCH1 |
| FES |
| BLM |
| ARID1A |
| DGKG |
| TSHB |
| RNF8 |
| RAD18 |
| SURF4 |
| CDH1 |
| TNFRSF9 |
| ACOT4 |
| CD46 |
| NONO |
| S100A4 |
| SUGT1 |
| MIRLET7B |
| ATG3 |
| TERF2IP |
| MYDGF |
| USF1 |
| MIR200B |
| SOAT1 |
| C3AR1 |
| CD47 |
| BAK1 |
| CD28 |
| NLRX1 |
| TSHR |
| RGCC |
| REL |
| RELB |
| AGXT2 |
| CCN1 |
| HTRA2 |
| CHUK |
| IRF5 |
| GRK3 |
| UBE2I |
| LTB |
| WNT5A |
| CDK9 |
| FGF5 |
| PALLD |
| KCNJ16 |
| VTI1A |
| SLC1A4 |
| EIF2AK1 |
| LYST |
| NT5C2 |
| SCARB1 |
| CD226 |
| SPARC |
| TSC2 |
| FEN1 |
| CD151 |
| TGIF1 |
| IGF2BP2 |
| PSMA7 |
| CD82 |
| SPTBN2 |
| GOSR1 |
| CHM |
| DNPEP |
| PEG3 |
| CKMT1A |
| MIR1306 |
| CYP1B1 |
| STX1A |
| PARP2 |
| GNMT |
| MAFB |
| MCM6 |
| ATP2B4 |
| LONP1 |
| PTPRD |
| DAB1 |
| MIR26A1 |
| DDIT4 |
| IL18BP |
| PSMB9 |
| PDE3B |
| AASS |
| COL6A1 |
| MTRR |
| ADH1C |
| FADS1 |
| PDGFC |
| NOL3 |
| ADAMTS7 |
| ZNF208 |
| E2F1 |
| PER2 |
| ANGPTL4 |
| MDK |
| FSTL1 |
| MIR24-2 |
| BEST1 |
| SP3 |
| CR2 |
| MYOM2 |
| FREM3 |
| NR4A1 |
| VAMP8 |
| AK1 |
| CFHR5 |
| SPARCL1 |
| MIR124-3 |
| SHH |
| SLC29A1 |
| CA4 |
| TCN2 |
| STRN |
| AQP10 |
| AMD1 |
| LRP2 |
| BNIP3 |
| STK4 |
| MIR200C |
| ESRRA |
| MS4A1 |
| SFRP1 |
| WDR12 |
| PPP3R1 |
| MSBP1 |
| FLT4 |
| TERF1 |
| PCSK2 |
| CCL4 |
| PSMB5 |
| SLC4A7 |
| PHKA1 |
| GSS |
| AQP3 |
| HSPA1L |
| GLS |
| TNFRSF25 |
| LCT |
| E2F2 |
| LACTB |
| CHEK1 |
| TNFRSF10B |
| JAK1 |
| CTBP1 |
| ALDH5A1 |
| F2RL3 |
| BIRC2 |
| TFF1 |
| LTBP2 |
| CDH15 |
| SBF1 |
| GLCE |
| AP3D1 |
| GADD45G |
| UHRF1 |
| SIX5 |
| TRDMT1 |
| SCG5 |
| ZNF79 |
| MYO7B |
| VEZF1 |
| MIR1-1 |
| VMP1 |
| CDK2 |
| CCND2 |
| PBX1 |
| RXRA |
| PRLR |
| GNRHR |
| DICER1 |
| RHO |
| CYP27B1 |
| KRT14 |
| LIPA |
| BARD1 |
| BIRC3 |
| ABCD1 |
| SDC2 |
| SLC27A4 |
| CARTPT |
| GIPR |
| RHOT1 |
| IRF9 |
| SRR |
| POLR1C |
| PYY |
| CCT3 |
| BBC3 |
| EPX |
| RAD52 |
| CUL4A |
| C4BPA |
| PLEK |
| ING5 |
| CASC3 |
| ADGRL1 |
| PHYHIP |
| ELOC |
| ELOB |
| FRMD4B |
| DUSP1 |
| PHKA2 |
| PSMD9 |
| MT2A |
| LGALS1 |
| CARD16 |
| KLF6 |
| GSTP1 |
| TRPM2 |
| TFAP2A |
| IGFBP5 |
| ALOX15B |
| PXDN |
| EVA1A |
| RBFOX1 |
| ABCC5 |
| MLST8 |
| NEK3 |
| EIF2AK4 |
| CLCN7 |
| ETS1 |
| THBS2 |
| ANXA2 |
| TLN2 |
| MOCS1 |
| SDC4 |
| CEBPA |
| MDH1 |
| HNRNPK |
| PCBP1 |
| HNRNPA1P10 |
| CGA |
| NOD1 |
| LUM |
| PPM1D |
| HSP90AB1 |
| GLRX |
| HDAC2 |
| DNAJA3 |
| NOD2 |
| PLD1 |
| CDH11 |
| KAT2B |
| KCNK1 |
| ZNF143 |
| FLRT2 |
| TMEM14C |
| CPNE8 |
| MIR497 |
| STAT6 |
| LRP6 |
| SLC6A8 |
| CRYM |
| SIRPA |
| TNFRSF18 |
| AGO2 |
| TRA2B |
| FHOD1 |
| RLN1 |
| PPARGC1B |
| CABIN1 |
| TREM1 |
| TXNIP |
| FGF13 |
| SUMO2 |
| EEF2 |
| S1PR3 |
| AOC1 |
| MIR15B |
| PTN |
| PRDX5 |
| RLN2 |
| APOO |
| ALPP |
| IGFBP7 |
| AKAP5 |
| PROKR1 |
| CXCL14 |
| MYOG |
| COL6A3 |
| FRZB |
| SENP2 |
| PGK1 |
| EGLN3 |
| CLEC3B |
| MIR508 |
| PPP2CA |
| A2M |
| COL18A1 |
| MCCC2 |
| MIP |
| SUCNR1 |
| AHR |
| ZEB1 |
| CCR7 |
| CNR2 |
| SLCO2B1 |
| ABCD4 |
| FNDC3B |
| AVPR2 |
| HCN2 |
| FOSL1 |
| FCER1G |
| COLEC11 |
| TNXB |
| ACOT7 |
| HOXA5 |
| NID1 |
| MYF5 |
| USP20 |
| ARVCF |
| BYSL |
| COL15A1 |
| CLEC4A |
| SPON1 |
| SIVA1 |
| IFN1@ |
| FZD4 |
| PRDX1 |
| ANPEP |
| RPL11 |
| PRMT1 |
| ASAH1 |
| C1QA |
| CDON |
| PARVA |
| ACAA2 |
| EEF1A1 |
| NRF1 |
| LAMC1 |
| COPS5 |
| ADAMTS3 |
| CAPZA2 |
| PPM1K |
| ABCB10 |
| ADAM11 |
| PRELP |
| MATN2 |
| SDF2 |
| RPS11 |
| TINAGL1 |
| WFDC2 |
| ADAMTS9 |
| DPT |
| RPL36AL |
| NID2 |
| WDR1 |
| ADAMTS20 |
| APOL4 |
| CHADL |
| MLF2 |
| RPL36A |
| NT5DC2 |
| DCTN6 |
| COL20A1 |
| TATDN1 |
| NAXE |
| SNU13 |
| NARS1 |
| UCN2 |
| COL6A5 |
| THEMIS2 |
| THUMPD2 |
| EMILIN3 |
| BEX4 |
| AGAP11 |
| SSPOP |
| MIR410 |
| PPCS |
| NEXN |
| ACTN2 |
| LIMS2 |
| XIRP1 |
| TNNC1 |
| SGCD |
| MYO6 |
| EYA4 |
| FKTN |
| MYPN |
| LDB3 |
| RBM20 |
| SHMT2 |
| ALPK3 |
| GP1BA |
| JPH2 |
| CMR3A |
| CMD1H |
| CMD1K |
| CMR1A |
| CMR2A |
| CMD1Q |
| CMD1B |
| CDK5RAP3 |
| YRDC |
| MELAS |
| LIMM |
| KLHL40 |
| KBTBD13 |
| TPM2 |
| CFL2 |
| NEB |
| SGCG |
| TNNT1 |
| SAMD7 |
| UGT2A1 |
| OBP2A |
| RTP3 |
| RTP4 |
| TAS2R16 |
| CHRNA7 |
| SCAMP3 |
| UFD1 |
| TAS2R14 |
| AGPS |
| DNAAF2 |
| OR1E2 |
| OR3A2 |
| TAS1R2 |
| TAS1R3 |
| TAS2R43 |
| OR10J1 |
| OR1D4 |
| SLC10A3 |
| PLCB2 |
| DRGX |
| PRG4 |
| CLCF1 |
| TRPA1 |
| PRS6KA5 |
| C5AR1 |
| CHF |
| LIFR |
| KLC3 |
| LHON |
| DIANPH |
| EVR3 |
| CMTX3 |
| CMTX2 |
| C4orf54 |
| ARMS2 |
| MAP1B |
| ETHE1 |
| LCLAT1 |
| PKN2 |
| MLC1 |
| AIRE |
| PNPLA6 |
| BCL10 |
| ECE2 |
| TAZ |
| SLC27A1 |

| **Table S3.** Sequence of compounds linkage gene targets |  |  |  |
| --- | --- | --- | --- |
| Node1 | Node2 | Net | Mol_Name |
| MOL001601 | PTGS1 | target | 1,2,5,6-tetrahydrotanshinone |
| MOL001601 | CHRM3 | target | 1,2,5,6-tetrahydrotanshinone |
| MOL001601 | CHRM1 | target | 1,2,5,6-tetrahydrotanshinone |
| MOL001601 | SCN5A | target | 1,2,5,6-tetrahydrotanshinone |
| MOL001601 | CHRM5 | target | 1,2,5,6-tetrahydrotanshinone |
| MOL001601 | PTGS2 | target | 1,2,5,6-tetrahydrotanshinone |
| MOL001601 | HTR3A | target | 1,2,5,6-tetrahydrotanshinone |
| MOL001601 | CA2 | target | 1,2,5,6-tetrahydrotanshinone |
| MOL001601 | CHRM4 | target | 1,2,5,6-tetrahydrotanshinone |
| MOL001601 | RXRA | target | 1,2,5,6-tetrahydrotanshinone |
| MOL001601 | OPRD1 | target | 1,2,5,6-tetrahydrotanshinone |
| MOL001601 | ADRA1A | target | 1,2,5,6-tetrahydrotanshinone |
| MOL001601 | CHRM2 | target | 1,2,5,6-tetrahydrotanshinone |
| MOL001601 | ADRA1B | target | 1,2,5,6-tetrahydrotanshinone |
| MOL001601 | SLC6A3 | target | 1,2,5,6-tetrahydrotanshinone |
| MOL001601 | ADRB2 | target | 1,2,5,6-tetrahydrotanshinone |
| MOL001601 | ADRA1D | target | 1,2,5,6-tetrahydrotanshinone |
| MOL001601 | OPRM1 | target | 1,2,5,6-tetrahydrotanshinone |
| MOL001601 | HSP90AB1 | target | 1,2,5,6-tetrahydrotanshinone |
| MOL001601 | DRD1 | target | 1,2,5,6-tetrahydrotanshinone |
| MOL001601 | SLC6A4 | target | 1,2,5,6-tetrahydrotanshinone |
| MOL001659 | NR3C2 | target | Poriferasterol |
| MOL001942 | PTGS2 | target | isoimperatorin |
| MOL002222 | CHRM3 | target | sugiol |
| MOL002222 | CHRM1 | target | sugiol |
| MOL002222 | SCN5A | target | sugiol |
| MOL002222 | CHRM5 | target | sugiol |
| MOL002222 | PTGS2 | target | sugiol |
| MOL002222 | CA2 | target | sugiol |
| MOL002222 | CHRM4 | target | sugiol |
| MOL002222 | OPRD1 | target | sugiol |
| MOL002222 | ACHE | target | sugiol |
| MOL002222 | ADRA1A | target | sugiol |
| MOL002222 | CHRM2 | target | sugiol |
| MOL002222 | ADRA1B | target | sugiol |
| MOL002222 | ADRB2 | target | sugiol |
| MOL002222 | ADRA1D | target | sugiol |
| MOL002222 | DRD2 | target | sugiol |
| MOL002222 | OPRM1 | target | sugiol |
| MOL002651 | DRD1 | target | Dehydrotanshinone II A |
| MOL002651 | CHRM3 | target | Dehydrotanshinone II A |
| MOL002651 | CHRM1 | target | Dehydrotanshinone II A |
| MOL002651 | ESR1 | target | Dehydrotanshinone II A |
| MOL002651 | AR | target | Dehydrotanshinone II A |
| MOL002651 | SCN5A | target | Dehydrotanshinone II A |
| MOL002651 | PPARG | target | Dehydrotanshinone II A |
| MOL002651 | CHRM5 | target | Dehydrotanshinone II A |
| MOL002651 | PTGS2 | target | Dehydrotanshinone II A |
| MOL002651 | CHRM4 | target | Dehydrotanshinone II A |
| MOL002651 | OPRD1 | target | Dehydrotanshinone II A |
| MOL002651 | ACHE | target | Dehydrotanshinone II A |
| MOL002651 | ADRA1A | target | Dehydrotanshinone II A |
| MOL002651 | ADRB2 | target | Dehydrotanshinone II A |
| MOL002651 | OPRM1 | target | Dehydrotanshinone II A |
| MOL002651 | DPP4 | target | Dehydrotanshinone II A |
| MOL000569 | PTGS2 | target | digallate |
| MOL000569 | HSP90AB1 | target | digallate |
| MOL000569 | AKR1B1 | target | digallate |
| MOL000006 | PTGS1 | target | luteolin |
| MOL000006 | AR | target | luteolin |
| MOL000006 | PTGS2 | target | luteolin |
| MOL000006 | HSP90AB1 | target | luteolin |
| MOL000006 | DPP4 | target | luteolin |
| MOL000006 | RELA | target | luteolin |
| MOL000006 | EGFR | target | luteolin |
| MOL000006 | AKT1 | target | luteolin |
| MOL000006 | VEGFA | target | luteolin |
| MOL000006 | CCND1 | target | luteolin |
| MOL000006 | BCL2L1 | target | luteolin |
| MOL000006 | CDKN1A | target | luteolin |
| MOL000006 | CASP9 | target | luteolin |
| MOL000006 | MMP2 | target | luteolin |
| MOL000006 | MMP9 | target | luteolin |
| MOL000006 | MAPK1 | target | luteolin |
| MOL000006 | RB1 | target | luteolin |
| MOL000006 | TNFAIP6 | target | luteolin |
| MOL000006 | JUN | target | luteolin |
| MOL000006 | IL6R | target | luteolin |
| MOL000006 | CASP3 | target | luteolin |
| MOL000006 | TP53 | target | luteolin |
| MOL000006 | NFKBIA | target | luteolin |
| MOL000006 | MDM2 | target | luteolin |
| MOL000006 | APP | target | luteolin |
| MOL000006 | MMP1 | target | luteolin |
| MOL000006 | PCNA | target | luteolin |
| MOL000006 | ERBB2 | target | luteolin |
| MOL000006 | PPARG | target | luteolin |
| MOL000006 | HMOX1 | target | luteolin |
| MOL000006 | CASP7 | target | luteolin |
| MOL000006 | ICAM1 | target | luteolin |
| MOL000006 | MCL1 | target | luteolin |
| MOL000006 | BIRC5 | target | luteolin |
| MOL000006 | IL2RA | target | luteolin |
| MOL000006 | TYR | target | luteolin |
| MOL000006 | IFNG | target | luteolin |
| MOL000006 | IL4 | target | luteolin |
| MOL000006 | GSTP1 | target | luteolin |
| MOL000006 | XIAP | target | luteolin |
| MOL000006 | SLC2A4 | target | luteolin |
| MOL000006 | CD40LG | target | luteolin |
| MOL000006 | MET | target | luteolin |
| MOL007036 | PTGS1 | target | 5,6-dihydroxy-7-isopropyl-1,1-dimethyl-2,3-dihydrophenanthren-4-one |
| MOL007036 | CHRM3 | target | 5,6-dihydroxy-7-isopropyl-1,1-dimethyl-2,3-dihydrophenanthren-4-one |
| MOL007036 | CHRM1 | target | 5,6-dihydroxy-7-isopropyl-1,1-dimethyl-2,3-dihydrophenanthren-4-one |
| MOL007036 | SCN5A | target | 5,6-dihydroxy-7-isopropyl-1,1-dimethyl-2,3-dihydrophenanthren-4-one |
| MOL007036 | PTGS2 | target | 5,6-dihydroxy-7-isopropyl-1,1-dimethyl-2,3-dihydrophenanthren-4-one |
| MOL007036 | CA2 | target | 5,6-dihydroxy-7-isopropyl-1,1-dimethyl-2,3-dihydrophenanthren-4-one |
| MOL007036 | RXRA | target | 5,6-dihydroxy-7-isopropyl-1,1-dimethyl-2,3-dihydrophenanthren-4-one |
| MOL007036 | ACHE | target | 5,6-dihydroxy-7-isopropyl-1,1-dimethyl-2,3-dihydrophenanthren-4-one |
| MOL007036 | ADRA1A | target | 5,6-dihydroxy-7-isopropyl-1,1-dimethyl-2,3-dihydrophenanthren-4-one |
| MOL007036 | ADRA1B | target | 5,6-dihydroxy-7-isopropyl-1,1-dimethyl-2,3-dihydrophenanthren-4-one |
| MOL007036 | ADRB2 | target | 5,6-dihydroxy-7-isopropyl-1,1-dimethyl-2,3-dihydrophenanthren-4-one |
| MOL007036 | OPRM1 | target | 5,6-dihydroxy-7-isopropyl-1,1-dimethyl-2,3-dihydrophenanthren-4-one |
| MOL007041 | PTGS1 | target | 2-isopropyl-8-methylphenanthrene-3,4-dione |
| MOL007041 | DRD1 | target | 2-isopropyl-8-methylphenanthrene-3,4-dione |
| MOL007041 | CHRM3 | target | 2-isopropyl-8-methylphenanthrene-3,4-dione |
| MOL007041 | CHRM1 | target | 2-isopropyl-8-methylphenanthrene-3,4-dione |
| MOL007041 | ESR1 | target | 2-isopropyl-8-methylphenanthrene-3,4-dione |
| MOL007041 | AR | target | 2-isopropyl-8-methylphenanthrene-3,4-dione |
| MOL007041 | SCN5A | target | 2-isopropyl-8-methylphenanthrene-3,4-dione |
| MOL007041 | PPARG | target | 2-isopropyl-8-methylphenanthrene-3,4-dione |
| MOL007041 | CHRM5 | target | 2-isopropyl-8-methylphenanthrene-3,4-dione |
| MOL007041 | PTGS2 | target | 2-isopropyl-8-methylphenanthrene-3,4-dione |
| MOL007041 | HTR3A | target | 2-isopropyl-8-methylphenanthrene-3,4-dione |
| MOL007041 | CHRM4 | target | 2-isopropyl-8-methylphenanthrene-3,4-dione |
| MOL007041 | RXRA | target | 2-isopropyl-8-methylphenanthrene-3,4-dione |
| MOL007041 | ADRA1A | target | 2-isopropyl-8-methylphenanthrene-3,4-dione |
| MOL007041 | CHRM2 | target | 2-isopropyl-8-methylphenanthrene-3,4-dione |
| MOL007041 | ADRA1B | target | 2-isopropyl-8-methylphenanthrene-3,4-dione |
| MOL007041 | SLC6A3 | target | 2-isopropyl-8-methylphenanthrene-3,4-dione |
| MOL007041 | ADRB2 | target | 2-isopropyl-8-methylphenanthrene-3,4-dione |
| MOL007041 | ADRA1D | target | 2-isopropyl-8-methylphenanthrene-3,4-dione |
| MOL007041 | SLC6A4 | target | 2-isopropyl-8-methylphenanthrene-3,4-dione |
| MOL007041 | OPRM1 | target | 2-isopropyl-8-methylphenanthrene-3,4-dione |
| MOL007041 | CDK2 | target | 2-isopropyl-8-methylphenanthrene-3,4-dione |
| MOL007045 | CHRM1 | target | 3α-hydroxytanshinoneⅡa |
| MOL007045 | SCN5A | target | 3α-hydroxytanshinoneⅡa |
| MOL007045 | CHRM5 | target | 3α-hydroxytanshinoneⅡa |
| MOL007045 | PTGS2 | target | 3α-hydroxytanshinoneⅡa |
| MOL007045 | OPRD1 | target | 3α-hydroxytanshinoneⅡa |
| MOL007045 | ACHE | target | 3α-hydroxytanshinoneⅡa |
| MOL007045 | ADRB2 | target | 3α-hydroxytanshinoneⅡa |
| MOL007045 | OPRM1 | target | 3α-hydroxytanshinoneⅡa |
| MOL007045 | DPP4 | target | 3α-hydroxytanshinoneⅡa |
| MOL007048 | PTGS2 | target | (E)-3-[2-(3,4-dihydroxyphenyl)-7-hydroxy-benzofuran-4-yl]acrylic acid |
| MOL007048 | HSP90AB1 | target | (E)-3-[2-(3,4-dihydroxyphenyl)-7-hydroxy-benzofuran-4-yl]acrylic acid |
| MOL007049 | PTGS1 | target | 4-methylenemiltirone |
| MOL007049 | DRD1 | target | 4-methylenemiltirone |
| MOL007049 | CHRM3 | target | 4-methylenemiltirone |
| MOL007049 | CHRM1 | target | 4-methylenemiltirone |
| MOL007049 | ESR1 | target | 4-methylenemiltirone |
| MOL007049 | AR | target | 4-methylenemiltirone |
| MOL007049 | SCN5A | target | 4-methylenemiltirone |
| MOL007049 | PPARG | target | 4-methylenemiltirone |
| MOL007049 | CHRM5 | target | 4-methylenemiltirone |
| MOL007049 | PTGS2 | target | 4-methylenemiltirone |
| MOL007049 | ADRA2A | target | 4-methylenemiltirone |
| MOL007049 | CA2 | target | 4-methylenemiltirone |
| MOL007049 | ADRA2C | target | 4-methylenemiltirone |
| MOL007049 | CHRM4 | target | 4-methylenemiltirone |
| MOL007049 | RXRA | target | 4-methylenemiltirone |
| MOL007049 | OPRD1 | target | 4-methylenemiltirone |
| MOL007049 | ADRA1A | target | 4-methylenemiltirone |
| MOL007049 | CHRM2 | target | 4-methylenemiltirone |
| MOL007049 | ADRA1B | target | 4-methylenemiltirone |
| MOL007049 | SLC6A3 | target | 4-methylenemiltirone |
| MOL007049 | ADRB2 | target | 4-methylenemiltirone |
| MOL007049 | ADRA1D | target | 4-methylenemiltirone |
| MOL007049 | SLC6A4 | target | 4-methylenemiltirone |
| MOL007049 | DRD2 | target | 4-methylenemiltirone |
| MOL007049 | OPRM1 | target | 4-methylenemiltirone |
| MOL007050 | NOS2 | target | 2-(4-hydroxy-3-methoxyphenyl)-5-(3-hydroxypropyl)-7-methoxy-3-benzofurancarboxaldehyde |
| MOL007050 | ESR1 | target | 2-(4-hydroxy-3-methoxyphenyl)-5-(3-hydroxypropyl)-7-methoxy-3-benzofurancarboxaldehyde |
| MOL007050 | AR | target | 2-(4-hydroxy-3-methoxyphenyl)-5-(3-hydroxypropyl)-7-methoxy-3-benzofurancarboxaldehyde |
| MOL007050 | PPARG | target | 2-(4-hydroxy-3-methoxyphenyl)-5-(3-hydroxypropyl)-7-methoxy-3-benzofurancarboxaldehyde |
| MOL007050 | ESR2 | target | 2-(4-hydroxy-3-methoxyphenyl)-5-(3-hydroxypropyl)-7-methoxy-3-benzofurancarboxaldehyde |
| MOL007050 | MAPK14 | target | 2-(4-hydroxy-3-methoxyphenyl)-5-(3-hydroxypropyl)-7-methoxy-3-benzofurancarboxaldehyde |
| MOL007050 | GSK3B | target | 2-(4-hydroxy-3-methoxyphenyl)-5-(3-hydroxypropyl)-7-methoxy-3-benzofurancarboxaldehyde |
| MOL007050 | HSP90AB1 | target | 2-(4-hydroxy-3-methoxyphenyl)-5-(3-hydroxypropyl)-7-methoxy-3-benzofurancarboxaldehyde |
| MOL007050 | CDK2 | target | 2-(4-hydroxy-3-methoxyphenyl)-5-(3-hydroxypropyl)-7-methoxy-3-benzofurancarboxaldehyde |
| MOL007058 | AR | target | formyltanshinone |
| MOL007058 | PTGS2 | target | formyltanshinone |
| MOL007058 | RXRA | target | formyltanshinone |
| MOL007058 | DPP4 | target | formyltanshinone |
| MOL007059 | DRD1 | target | 3-beta-Hydroxymethyllenetanshiquinone |
| MOL007059 | CHRM1 | target | 3-beta-Hydroxymethyllenetanshiquinone |
| MOL007059 | PTGS2 | target | 3-beta-Hydroxymethyllenetanshiquinone |
| MOL007059 | CA2 | target | 3-beta-Hydroxymethyllenetanshiquinone |
| MOL007059 | RXRA | target | 3-beta-Hydroxymethyllenetanshiquinone |
| MOL007059 | OPRD1 | target | 3-beta-Hydroxymethyllenetanshiquinone |
| MOL007059 | ACHE | target | 3-beta-Hydroxymethyllenetanshiquinone |
| MOL007059 | ADRA1A | target | 3-beta-Hydroxymethyllenetanshiquinone |
| MOL007059 | ADRB2 | target | 3-beta-Hydroxymethyllenetanshiquinone |
| MOL007059 | OPRM1 | target | 3-beta-Hydroxymethyllenetanshiquinone |
| MOL007059 | DPP4 | target | 3-beta-Hydroxymethyllenetanshiquinone |
| MOL007059 | HSP90AB1 | target | 3-beta-Hydroxymethyllenetanshiquinone |
| MOL007061 | DRD1 | target | Methylenetanshinquinone |
| MOL007061 | CHRM3 | target | Methylenetanshinquinone |
| MOL007061 | CHRM1 | target | Methylenetanshinquinone |
| MOL007061 | SCN5A | target | Methylenetanshinquinone |
| MOL007061 | CHRM5 | target | Methylenetanshinquinone |
| MOL007061 | PTGS2 | target | Methylenetanshinquinone |
| MOL007061 | CA2 | target | Methylenetanshinquinone |
| MOL007061 | RXRA | target | Methylenetanshinquinone |
| MOL007061 | OPRD1 | target | Methylenetanshinquinone |
| MOL007061 | ACHE | target | Methylenetanshinquinone |
| MOL007061 | ADRA1A | target | Methylenetanshinquinone |
| MOL007061 | CHRM2 | target | Methylenetanshinquinone |
| MOL007061 | ADRB2 | target | Methylenetanshinquinone |
| MOL007061 | SLC6A4 | target | Methylenetanshinquinone |
| MOL007061 | OPRM1 | target | Methylenetanshinquinone |
| MOL007061 | DPP4 | target | Methylenetanshinquinone |
| MOL007061 | HSP90AB1 | target | Methylenetanshinquinone |
| MOL007063 | NR3C2 | target | przewalskin a |
| MOL007063 | NR3C1 | target | przewalskin a |
| MOL007064 | PTGS2 | target | przewalskin b |
| MOL007064 | NR3C2 | target | przewalskin b |
| MOL007064 | NR3C1 | target | przewalskin b |
| MOL007068 | PTGS2 | target | Przewaquinone B |
| MOL007068 | RXRA | target | Przewaquinone B |
| MOL007068 | DPP4 | target | Przewaquinone B |
| MOL007068 | HSP90AB1 | target | Przewaquinone B |
| MOL007069 | PTGS1 | target | przewaquinone c |
| MOL007069 | DRD1 | target | przewaquinone c |
| MOL007069 | CHRM3 | target | przewaquinone c |
| MOL007069 | CHRM1 | target | przewaquinone c |
| MOL007069 | SCN5A | target | przewaquinone c |
| MOL007069 | CHRM5 | target | przewaquinone c |
| MOL007069 | PTGS2 | target | przewaquinone c |
| MOL007069 | CA2 | target | przewaquinone c |
| MOL007069 | CHRM4 | target | przewaquinone c |
| MOL007069 | OPRD1 | target | przewaquinone c |
| MOL007069 | ACHE | target | przewaquinone c |
| MOL007069 | ADRA1A | target | przewaquinone c |
| MOL007069 | CHRM2 | target | przewaquinone c |
| MOL007069 | ADRB2 | target | przewaquinone c |
| MOL007069 | OPRM1 | target | przewaquinone c |
| MOL007069 | DPP4 | target | przewaquinone c |
| MOL007069 | HSP90AB1 | target | przewaquinone c |
| MOL007070 | PTGS2 | target | (6S,7R)-6,7-dihydroxy-1,6-dimethyl-8,9-dihydro-7H-naphtho[8,7-g]benzofuran-10,11-dione |
| MOL007070 | CA2 | target | (6S,7R)-6,7-dihydroxy-1,6-dimethyl-8,9-dihydro-7H-naphtho[8,7-g]benzofuran-10,11-dione |
| MOL007070 | ACHE | target | (6S,7R)-6,7-dihydroxy-1,6-dimethyl-8,9-dihydro-7H-naphtho[8,7-g]benzofuran-10,11-dione |
| MOL007070 | DPP4 | target | (6S,7R)-6,7-dihydroxy-1,6-dimethyl-8,9-dihydro-7H-naphtho[8,7-g]benzofuran-10,11-dione |
| MOL007070 | HSP90AB1 | target | (6S,7R)-6,7-dihydroxy-1,6-dimethyl-8,9-dihydro-7H-naphtho[8,7-g]benzofuran-10,11-dione |
| MOL007071 | PTGS2 | target | przewaquinone f |
| MOL007071 | DPP4 | target | przewaquinone f |
| MOL007077 | PTGS2 | target | sclareol |
| MOL007079 | DRD1 | target | tanshinaldehyde |
| MOL007079 | CHRM1 | target | tanshinaldehyde |
| MOL007079 | PTGS2 | target | tanshinaldehyde |
| MOL007079 | OPRD1 | target | tanshinaldehyde |
| MOL007079 | ACHE | target | tanshinaldehyde |
| MOL007079 | ADRB2 | target | tanshinaldehyde |
| MOL007079 | OPRM1 | target | tanshinaldehyde |
| MOL007079 | DPP4 | target | tanshinaldehyde |
| MOL007081 | PTGS2 | target | Danshenol B |
| MOL007081 | CA2 | target | Danshenol B |
| MOL007081 | OPRM1 | target | Danshenol B |
| MOL007081 | NR3C1 | target | Danshenol B |
| MOL007081 | HSP90AB1 | target | Danshenol B |
| MOL007082 | PTGS1 | target | Danshenol A |
| MOL007082 | KCNH2 | target | Danshenol A |
| MOL007082 | SCN5A | target | Danshenol A |
| MOL007082 | PTGS2 | target | Danshenol A |
| MOL007082 | RXRA | target | Danshenol A |
| MOL007085 | PTGS1 | target | Salvilenone |
| MOL007085 | ESR1 | target | Salvilenone |
| MOL007085 | AR | target | Salvilenone |
| MOL007085 | CHRM5 | target | Salvilenone |
| MOL007085 | PTGS2 | target | Salvilenone |
| MOL007085 | HTR3A | target | Salvilenone |
| MOL007085 | ESR2 | target | Salvilenone |
| MOL007088 | PTGS1 | target | cryptotanshinone |
| MOL007088 | DRD1 | target | cryptotanshinone |
| MOL007088 | CHRM3 | target | cryptotanshinone |
| MOL007088 | CHRM1 | target | cryptotanshinone |
| MOL007088 | SCN5A | target | cryptotanshinone |
| MOL007088 | CHRM5 | target | cryptotanshinone |
| MOL007088 | PTGS2 | target | cryptotanshinone |
| MOL007088 | CA2 | target | cryptotanshinone |
| MOL007088 | CHRM4 | target | cryptotanshinone |
| MOL007088 | OPRD1 | target | cryptotanshinone |
| MOL007088 | ADRA1A | target | cryptotanshinone |
| MOL007088 | CHRM2 | target | cryptotanshinone |
| MOL007088 | ADRA1B | target | cryptotanshinone |
| MOL007088 | ADRB2 | target | cryptotanshinone |
| MOL007088 | ADRA1D | target | cryptotanshinone |
| MOL007088 | OPRM1 | target | cryptotanshinone |
| MOL007088 | RELA | target | cryptotanshinone |
| MOL007088 | STAT3 | target | cryptotanshinone |
| MOL007088 | CCND1 | target | cryptotanshinone |
| MOL007088 | BCL2L1 | target | cryptotanshinone |
| MOL007088 | TNFAIP6 | target | cryptotanshinone |
| MOL007088 | APP | target | cryptotanshinone |
| MOL007088 | EDN1 | target | cryptotanshinone |
| MOL007088 | BIRC5 | target | cryptotanshinone |
| MOL007093 | NOS2 | target | dan-shexinkum d |
| MOL007093 | PTGS1 | target | dan-shexinkum d |
| MOL007093 | KCNH2 | target | dan-shexinkum d |
| MOL007093 | CHRM1 | target | dan-shexinkum d |
| MOL007093 | ESR1 | target | dan-shexinkum d |
| MOL007093 | AR | target | dan-shexinkum d |
| MOL007093 | SCN5A | target | dan-shexinkum d |
| MOL007093 | PPARG | target | dan-shexinkum d |
| MOL007093 | PTGS2 | target | dan-shexinkum d |
| MOL007093 | CA2 | target | dan-shexinkum d |
| MOL007093 | RXRA | target | dan-shexinkum d |
| MOL007093 | ACHE | target | dan-shexinkum d |
| MOL007093 | ADRA1B | target | dan-shexinkum d |
| MOL007093 | ADRB2 | target | dan-shexinkum d |
| MOL007093 | ESR2 | target | dan-shexinkum d |
| MOL007093 | DPP4 | target | dan-shexinkum d |
| MOL007093 | GSK3B | target | dan-shexinkum d |
| MOL007093 | CDK2 | target | dan-shexinkum d |
| MOL007093 | CHEK1 | target | dan-shexinkum d |
| MOL007094 | PTGS1 | target | danshenspiroketallactone |
| MOL007094 | DRD1 | target | danshenspiroketallactone |
| MOL007094 | CHRM3 | target | danshenspiroketallactone |
| MOL007094 | CHRM1 | target | danshenspiroketallactone |
| MOL007094 | ESR1 | target | danshenspiroketallactone |
| MOL007094 | SCN5A | target | danshenspiroketallactone |
| MOL007094 | CHRM5 | target | danshenspiroketallactone |
| MOL007094 | PTGS2 | target | danshenspiroketallactone |
| MOL007094 | CA2 | target | danshenspiroketallactone |
| MOL007094 | CHRM4 | target | danshenspiroketallactone |
| MOL007094 | RXRA | target | danshenspiroketallactone |
| MOL007094 | ACHE | target | danshenspiroketallactone |
| MOL007094 | ADRA1A | target | danshenspiroketallactone |
| MOL007094 | CHRM2 | target | danshenspiroketallactone |
| MOL007094 | ADRA1B | target | danshenspiroketallactone |
| MOL007094 | ADRB2 | target | danshenspiroketallactone |
| MOL007094 | ADRA1D | target | danshenspiroketallactone |
| MOL007094 | SLC6A4 | target | danshenspiroketallactone |
| MOL007094 | OPRM1 | target | danshenspiroketallactone |
| MOL007094 | DPP4 | target | danshenspiroketallactone |
| MOL007094 | HSP90AB1 | target | danshenspiroketallactone |
| MOL007098 | PTGS1 | target | deoxyneocryptotanshinone |
| MOL007098 | DRD1 | target | deoxyneocryptotanshinone |
| MOL007098 | CHRM3 | target | deoxyneocryptotanshinone |
| MOL007098 | CHRM1 | target | deoxyneocryptotanshinone |
| MOL007098 | ESR1 | target | deoxyneocryptotanshinone |
| MOL007098 | AR | target | deoxyneocryptotanshinone |
| MOL007098 | SCN5A | target | deoxyneocryptotanshinone |
| MOL007098 | CHRM5 | target | deoxyneocryptotanshinone |
| MOL007098 | PTGS2 | target | deoxyneocryptotanshinone |
| MOL007098 | CA2 | target | deoxyneocryptotanshinone |
| MOL007098 | CHRM4 | target | deoxyneocryptotanshinone |
| MOL007098 | RXRA | target | deoxyneocryptotanshinone |
| MOL007098 | OPRD1 | target | deoxyneocryptotanshinone |
| MOL007098 | ADRA1A | target | deoxyneocryptotanshinone |
| MOL007098 | CHRM2 | target | deoxyneocryptotanshinone |
| MOL007098 | ADRA1B | target | deoxyneocryptotanshinone |
| MOL007098 | ADRB2 | target | deoxyneocryptotanshinone |
| MOL007098 | ADRA1D | target | deoxyneocryptotanshinone |
| MOL007098 | OPRM1 | target | deoxyneocryptotanshinone |
| MOL007098 | GSK3B | target | deoxyneocryptotanshinone |
| MOL007098 | CDK2 | target | deoxyneocryptotanshinone |
| MOL007100 | NOS2 | target | dihydrotanshinlactone |
| MOL007100 | PTGS1 | target | dihydrotanshinlactone |
| MOL007100 | DRD1 | target | dihydrotanshinlactone |
| MOL007100 | CHRM3 | target | dihydrotanshinlactone |
| MOL007100 | CHRM1 | target | dihydrotanshinlactone |
| MOL007100 | ESR1 | target | dihydrotanshinlactone |
| MOL007100 | AR | target | dihydrotanshinlactone |
| MOL007100 | SCN5A | target | dihydrotanshinlactone |
| MOL007100 | PPARG | target | dihydrotanshinlactone |
| MOL007100 | CHRM5 | target | dihydrotanshinlactone |
| MOL007100 | PTGS2 | target | dihydrotanshinlactone |
| MOL007100 | HTR3A | target | dihydrotanshinlactone |
| MOL007100 | CA2 | target | dihydrotanshinlactone |
| MOL007100 | RXRA | target | dihydrotanshinlactone |
| MOL007100 | ACHE | target | dihydrotanshinlactone |
| MOL007100 | ADRA1A | target | dihydrotanshinlactone |
| MOL007100 | ADRA1B | target | dihydrotanshinlactone |
| MOL007100 | SLC6A3 | target | dihydrotanshinlactone |
| MOL007100 | ADRB2 | target | dihydrotanshinlactone |
| MOL007100 | ADRA1D | target | dihydrotanshinlactone |
| MOL007100 | SLC6A4 | target | dihydrotanshinlactone |
| MOL007100 | OPRM1 | target | dihydrotanshinlactone |
| MOL007100 | DPP4 | target | dihydrotanshinlactone |
| MOL007100 | GSK3B | target | dihydrotanshinlactone |
| MOL007101 | PTGS1 | target | dihydrotanshinoneⅠ |
| MOL007101 | SCN5A | target | dihydrotanshinoneⅠ |
| MOL007101 | PTGS2 | target | dihydrotanshinoneⅠ |
| MOL007101 | HTR3A | target | dihydrotanshinoneⅠ |
| MOL007101 | RXRA | target | dihydrotanshinoneⅠ |
| MOL007101 | ADRA1A | target | dihydrotanshinoneⅠ |
| MOL007101 | ADRA1B | target | dihydrotanshinoneⅠ |
| MOL007101 | ADRB2 | target | dihydrotanshinoneⅠ |
| MOL007101 | HSP90AB1 | target | dihydrotanshinoneⅠ |
| MOL007105 | PTGS1 | target | epidanshenspiroketallactone |
| MOL007105 | DRD1 | target | epidanshenspiroketallactone |
| MOL007105 | CHRM3 | target | epidanshenspiroketallactone |
| MOL007105 | CHRM1 | target | epidanshenspiroketallactone |
| MOL007105 | ESR1 | target | epidanshenspiroketallactone |
| MOL007105 | SCN5A | target | epidanshenspiroketallactone |
| MOL007105 | CHRM5 | target | epidanshenspiroketallactone |
| MOL007105 | PTGS2 | target | epidanshenspiroketallactone |
| MOL007105 | CHRM4 | target | epidanshenspiroketallactone |
| MOL007105 | RXRA | target | epidanshenspiroketallactone |
| MOL007105 | OPRD1 | target | epidanshenspiroketallactone |
| MOL007105 | ADRA1A | target | epidanshenspiroketallactone |
| MOL007105 | CHRM2 | target | epidanshenspiroketallactone |
| MOL007105 | ADRA1B | target | epidanshenspiroketallactone |
| MOL007105 | ADRB2 | target | epidanshenspiroketallactone |
| MOL007105 | ADRA1D | target | epidanshenspiroketallactone |
| MOL007105 | SLC6A4 | target | epidanshenspiroketallactone |
| MOL007105 | OPRM1 | target | epidanshenspiroketallactone |
| MOL007105 | HSP90AB1 | target | epidanshenspiroketallactone |
| MOL007105 | CDK2 | target | epidanshenspiroketallactone |
| MOL007107 | CHRM3 | target | C09092 |
| MOL007107 | CHRM1 | target | C09092 |
| MOL007107 | SCN5A | target | C09092 |
| MOL007107 | CA2 | target | C09092 |
| MOL007107 | ACHE | target | C09092 |
| MOL007107 | ADRA1A | target | C09092 |
| MOL007107 | CHRM2 | target | C09092 |
| MOL007107 | ADRA1B | target | C09092 |
| MOL007107 | ADRB2 | target | C09092 |
| MOL007107 | ADRA1D | target | C09092 |
| MOL007107 | OPRM1 | target | C09092 |
| MOL007108 | NOS2 | target | isocryptotanshi-none |
| MOL007108 | PTGS1 | target | isocryptotanshi-none |
| MOL007108 | DRD1 | target | isocryptotanshi-none |
| MOL007108 | CHRM3 | target | isocryptotanshi-none |
| MOL007108 | CHRM1 | target | isocryptotanshi-none |
| MOL007108 | ESR1 | target | isocryptotanshi-none |
| MOL007108 | AR | target | isocryptotanshi-none |
| MOL007108 | SCN5A | target | isocryptotanshi-none |
| MOL007108 | CHRM5 | target | isocryptotanshi-none |
| MOL007108 | PTGS2 | target | isocryptotanshi-none |
| MOL007108 | CA2 | target | isocryptotanshi-none |
| MOL007108 | CHRM4 | target | isocryptotanshi-none |
| MOL007108 | RXRA | target | isocryptotanshi-none |
| MOL007108 | OPRD1 | target | isocryptotanshi-none |
| MOL007108 | ACHE | target | isocryptotanshi-none |
| MOL007108 | ADRA1A | target | isocryptotanshi-none |
| MOL007108 | CHRM2 | target | isocryptotanshi-none |
| MOL007108 | ADRA1B | target | isocryptotanshi-none |
| MOL007108 | ADRB2 | target | isocryptotanshi-none |
| MOL007108 | ADRA1D | target | isocryptotanshi-none |
| MOL007108 | DRD2 | target | isocryptotanshi-none |
| MOL007108 | OPRM1 | target | isocryptotanshi-none |
| MOL007108 | CDK2 | target | isocryptotanshi-none |
| MOL007111 | NOS2 | target | Isotanshinone II |
| MOL007111 | DRD1 | target | Isotanshinone II |
| MOL007111 | CHRM3 | target | Isotanshinone II |
| MOL007111 | CHRM1 | target | Isotanshinone II |
| MOL007111 | ESR1 | target | Isotanshinone II |
| MOL007111 | AR | target | Isotanshinone II |
| MOL007111 | SCN5A | target | Isotanshinone II |
| MOL007111 | CHRM5 | target | Isotanshinone II |
| MOL007111 | PTGS2 | target | Isotanshinone II |
| MOL007111 | RXRA | target | Isotanshinone II |
| MOL007111 | OPRD1 | target | Isotanshinone II |
| MOL007111 | ACHE | target | Isotanshinone II |
| MOL007111 | ADRA1A | target | Isotanshinone II |
| MOL007111 | CHRM2 | target | Isotanshinone II |
| MOL007111 | ADRB2 | target | Isotanshinone II |
| MOL007111 | OPRM1 | target | Isotanshinone II |
| MOL007111 | ESR2 | target | Isotanshinone II |
| MOL007111 | DPP4 | target | Isotanshinone II |
| MOL007111 | GSK3B | target | Isotanshinone II |
| MOL007111 | CDK2 | target | Isotanshinone II |
| MOL007111 | CHEK1 | target | Isotanshinone II |
| MOL007119 | PTGS1 | target | miltionone Ⅰ |
| MOL007119 | CHRM3 | target | miltionone Ⅰ |
| MOL007119 | CHRM1 | target | miltionone Ⅰ |
| MOL007119 | ESR1 | target | miltionone Ⅰ |
| MOL007119 | AR | target | miltionone Ⅰ |
| MOL007119 | SCN5A | target | miltionone Ⅰ |
| MOL007119 | PTGS2 | target | miltionone Ⅰ |
| MOL007119 | CA2 | target | miltionone Ⅰ |
| MOL007119 | RXRA | target | miltionone Ⅰ |
| MOL007119 | OPRD1 | target | miltionone Ⅰ |
| MOL007119 | ADRA1A | target | miltionone Ⅰ |
| MOL007119 | CHRM2 | target | miltionone Ⅰ |
| MOL007119 | ADRA1B | target | miltionone Ⅰ |
| MOL007119 | ADRB2 | target | miltionone Ⅰ |
| MOL007119 | OPRM1 | target | miltionone Ⅰ |
| MOL007119 | NR3C1 | target | miltionone Ⅰ |
| MOL007119 | GSK3B | target | miltionone Ⅰ |
| MOL007119 | CDK2 | target | miltionone Ⅰ |
| MOL007120 | PTGS2 | target | miltionone Ⅱ |
| MOL007120 | CA2 | target | miltionone Ⅱ |
| MOL007120 | ACHE | target | miltionone Ⅱ |
| MOL007120 | NR3C1 | target | miltionone Ⅱ |
| MOL007121 | ESR1 | target | miltipolone |
| MOL007121 | ACHE | target | miltipolone |
| MOL007122 | PTGS1 | target | Miltirone |
| MOL007122 | DRD1 | target | Miltirone |
| MOL007122 | CHRM3 | target | Miltirone |
| MOL007122 | CHRM1 | target | Miltirone |
| MOL007122 | ESR1 | target | Miltirone |
| MOL007122 | AR | target | Miltirone |
| MOL007122 | DRD5 | target | Miltirone |
| MOL007122 | SCN5A | target | Miltirone |
| MOL007122 | CHRM5 | target | Miltirone |
| MOL007122 | PTGS2 | target | Miltirone |
| MOL007122 | CA2 | target | Miltirone |
| MOL007122 | ADRA2C | target | Miltirone |
| MOL007122 | CHRM4 | target | Miltirone |
| MOL007122 | RXRA | target | Miltirone |
| MOL007122 | OPRD1 | target | Miltirone |
| MOL007122 | ADRA1A | target | Miltirone |
| MOL007122 | CHRM2 | target | Miltirone |
| MOL007122 | ADRA1B | target | Miltirone |
| MOL007122 | SLC6A3 | target | Miltirone |
| MOL007122 | ADRB2 | target | Miltirone |
| MOL007122 | ADRA1D | target | Miltirone |
| MOL007122 | OPRM1 | target | Miltirone |
| MOL007124 | PTGS1 | target | neocryptotanshinone ii |
| MOL007124 | DRD1 | target | neocryptotanshinone ii |
| MOL007124 | CHRM3 | target | neocryptotanshinone ii |
| MOL007124 | CHRM1 | target | neocryptotanshinone ii |
| MOL007124 | ESR1 | target | neocryptotanshinone ii |
| MOL007124 | AR | target | neocryptotanshinone ii |
| MOL007124 | SCN5A | target | neocryptotanshinone ii |
| MOL007124 | PTGS2 | target | neocryptotanshinone ii |
| MOL007124 | CA2 | target | neocryptotanshinone ii |
| MOL007124 | CHRM4 | target | neocryptotanshinone ii |
| MOL007124 | RXRA | target | neocryptotanshinone ii |
| MOL007124 | OPRD1 | target | neocryptotanshinone ii |
| MOL007124 | ADRA1A | target | neocryptotanshinone ii |
| MOL007124 | CHRM2 | target | neocryptotanshinone ii |
| MOL007124 | ADRA1B | target | neocryptotanshinone ii |
| MOL007124 | SLC6A3 | target | neocryptotanshinone ii |
| MOL007124 | ADRB2 | target | neocryptotanshinone ii |
| MOL007124 | ADRA1D | target | neocryptotanshinone ii |
| MOL007124 | SLC6A4 | target | neocryptotanshinone ii |
| MOL007124 | OPRM1 | target | neocryptotanshinone ii |
| MOL007124 | GSK3B | target | neocryptotanshinone ii |
| MOL007124 | HSP90AB1 | target | neocryptotanshinone ii |
| MOL007124 | CDK2 | target | neocryptotanshinone ii |
| MOL007125 | PTGS1 | target | neocryptotanshinone |
| MOL007125 | CHRM3 | target | neocryptotanshinone |
| MOL007125 | CHRM1 | target | neocryptotanshinone |
| MOL007125 | SCN5A | target | neocryptotanshinone |
| MOL007125 | PPARG | target | neocryptotanshinone |
| MOL007125 | PTGS2 | target | neocryptotanshinone |
| MOL007125 | CA2 | target | neocryptotanshinone |
| MOL007125 | ADRA1B | target | neocryptotanshinone |
| MOL007125 | ADRB2 | target | neocryptotanshinone |
| MOL007125 | ADRA1D | target | neocryptotanshinone |
| MOL007125 | OPRM1 | target | neocryptotanshinone |
| MOL007127 | PTGS1 | target | 1-methyl-8,9-dihydro-7H-naphtho[5,6-g]benzofuran-6,10,11-trione |
| MOL007127 | DRD1 | target | 1-methyl-8,9-dihydro-7H-naphtho[5,6-g]benzofuran-6,10,11-trione |
| MOL007127 | CHRM3 | target | 1-methyl-8,9-dihydro-7H-naphtho[5,6-g]benzofuran-6,10,11-trione |
| MOL007127 | SCN5A | target | 1-methyl-8,9-dihydro-7H-naphtho[5,6-g]benzofuran-6,10,11-trione |
| MOL007127 | CHRM5 | target | 1-methyl-8,9-dihydro-7H-naphtho[5,6-g]benzofuran-6,10,11-trione |
| MOL007127 | PTGS2 | target | 1-methyl-8,9-dihydro-7H-naphtho[5,6-g]benzofuran-6,10,11-trione |
| MOL007127 | CA2 | target | 1-methyl-8,9-dihydro-7H-naphtho[5,6-g]benzofuran-6,10,11-trione |
| MOL007127 | RXRA | target | 1-methyl-8,9-dihydro-7H-naphtho[5,6-g]benzofuran-6,10,11-trione |
| MOL007127 | ACHE | target | 1-methyl-8,9-dihydro-7H-naphtho[5,6-g]benzofuran-6,10,11-trione |
| MOL007127 | ADRA1A | target | 1-methyl-8,9-dihydro-7H-naphtho[5,6-g]benzofuran-6,10,11-trione |
| MOL007127 | ADRB2 | target | 1-methyl-8,9-dihydro-7H-naphtho[5,6-g]benzofuran-6,10,11-trione |
| MOL007127 | OPRM1 | target | 1-methyl-8,9-dihydro-7H-naphtho[5,6-g]benzofuran-6,10,11-trione |
| MOL007127 | DPP4 | target | 1-methyl-8,9-dihydro-7H-naphtho[5,6-g]benzofuran-6,10,11-trione |
| MOL007127 | HSP90AB1 | target | 1-methyl-8,9-dihydro-7H-naphtho[5,6-g]benzofuran-6,10,11-trione |
| MOL007130 | NOS2 | target | prolithospermic acid |
| MOL007130 | PTGS1 | target | prolithospermic acid |
| MOL007130 | ESR1 | target | prolithospermic acid |
| MOL007130 | AR | target | prolithospermic acid |
| MOL007130 | PTGS2 | target | prolithospermic acid |
| MOL007130 | HSP90AB1 | target | prolithospermic acid |
| MOL007132 | ESR1 | target | (2R)-3-(3,4-dihydroxyphenyl)-2-[(Z)-3-(3,4-dihydroxyphenyl)acryloyl]oxy-propionic acid |
| MOL007132 | AR | target | (2R)-3-(3,4-dihydroxyphenyl)-2-[(Z)-3-(3,4-dihydroxyphenyl)acryloyl]oxy-propionic acid |
| MOL007132 | PPARG | target | (2R)-3-(3,4-dihydroxyphenyl)-2-[(Z)-3-(3,4-dihydroxyphenyl)acryloyl]oxy-propionic acid |
| MOL007132 | PTGS2 | target | (2R)-3-(3,4-dihydroxyphenyl)-2-[(Z)-3-(3,4-dihydroxyphenyl)acryloyl]oxy-propionic acid |
| MOL007132 | DPP4 | target | (2R)-3-(3,4-dihydroxyphenyl)-2-[(Z)-3-(3,4-dihydroxyphenyl)acryloyl]oxy-propionic acid |
| MOL007141 | PTGS2 | target | salvianolic acid g |
| MOL007143 | PTGS2 | target | salvilenone Ⅰ |
| MOL007143 | RXRA | target | salvilenone Ⅰ |
| MOL007143 | ACHE | target | salvilenone Ⅰ |
| MOL007143 | NR3C1 | target | salvilenone Ⅰ |
| MOL007145 | PTGS1 | target | salviolone |
| MOL007145 | DRD1 | target | salviolone |
| MOL007145 | CHRM3 | target | salviolone |
| MOL007145 | CHRM1 | target | salviolone |
| MOL007145 | DRD5 | target | salviolone |
| MOL007145 | SCN5A | target | salviolone |
| MOL007145 | CHRM5 | target | salviolone |
| MOL007145 | PTGS2 | target | salviolone |
| MOL007145 | ADRA2A | target | salviolone |
| MOL007145 | HTR3A | target | salviolone |
| MOL007145 | CHRM4 | target | salviolone |
| MOL007145 | OPRD1 | target | salviolone |
| MOL007145 | ACHE | target | salviolone |
| MOL007145 | SLC6A2 | target | salviolone |
| MOL007145 | ADRA1A | target | salviolone |
| MOL007145 | CHRM2 | target | salviolone |
| MOL007145 | ADRA2B | target | salviolone |
| MOL007145 | ADRA1B | target | salviolone |
| MOL007145 | SLC6A3 | target | salviolone |
| MOL007145 | ADRB2 | target | salviolone |
| MOL007145 | SLC6A4 | target | salviolone |
| MOL007145 | DRD2 | target | salviolone |
| MOL007145 | OPRM1 | target | salviolone |
| MOL007150 | PTGS2 | target | (6S)-6-hydroxy-1-methyl-6-methylol-8,9-dihydro-7H-naphtho[8,7-g]benzofuran-10,11-quinone |
| MOL007150 | CA2 | target | (6S)-6-hydroxy-1-methyl-6-methylol-8,9-dihydro-7H-naphtho[8,7-g]benzofuran-10,11-quinone |
| MOL007150 | ACHE | target | (6S)-6-hydroxy-1-methyl-6-methylol-8,9-dihydro-7H-naphtho[8,7-g]benzofuran-10,11-quinone |
| MOL007150 | DPP4 | target | (6S)-6-hydroxy-1-methyl-6-methylol-8,9-dihydro-7H-naphtho[8,7-g]benzofuran-10,11-quinone |
| MOL007150 | HSP90AB1 | target | (6S)-6-hydroxy-1-methyl-6-methylol-8,9-dihydro-7H-naphtho[8,7-g]benzofuran-10,11-quinone |
| MOL007151 | PTGS2 | target | Tanshindiol B |
| MOL007151 | CA2 | target | Tanshindiol B |
| MOL007151 | ACHE | target | Tanshindiol B |
| MOL007151 | DPP4 | target | Tanshindiol B |
| MOL007151 | HSP90AB1 | target | Tanshindiol B |
| MOL007152 | PTGS2 | target | Przewaquinone E |
| MOL007152 | CA2 | target | Przewaquinone E |
| MOL007152 | ACHE | target | Przewaquinone E |
| MOL007152 | DPP4 | target | Przewaquinone E |
| MOL007152 | HSP90AB1 | target | Przewaquinone E |
| MOL007154 | DRD1 | target | tanshinone iia |
| MOL007154 | CHRM3 | target | tanshinone iia |
| MOL007154 | CHRM1 | target | tanshinone iia |
| MOL007154 | SCN5A | target | tanshinone iia |
| MOL007154 | CHRM5 | target | tanshinone iia |
| MOL007154 | PTGS2 | target | tanshinone iia |
| MOL007154 | CHRM4 | target | tanshinone iia |
| MOL007154 | OPRD1 | target | tanshinone iia |
| MOL007154 | ACHE | target | tanshinone iia |
| MOL007154 | ADRA1A | target | tanshinone iia |
| MOL007154 | CHRM2 | target | tanshinone iia |
| MOL007154 | ADRB2 | target | tanshinone iia |
| MOL007154 | OPRM1 | target | tanshinone iia |
| MOL007154 | DPP4 | target | tanshinone iia |
| MOL007154 | RXRA | target | tanshinone iia |
| MOL007154 | RELA | target | tanshinone iia |
| MOL007154 | BCL2 | target | tanshinone iia |
| MOL007154 | FOS | target | tanshinone iia |
| MOL007154 | CDKN1A | target | tanshinone iia |
| MOL007154 | MMP9 | target | tanshinone iia |
| MOL007154 | JUN | target | tanshinone iia |
| MOL007154 | CASP3 | target | tanshinone iia |
| MOL007154 | TP53 | target | tanshinone iia |
| MOL007154 | NFKBIA | target | tanshinone iia |
| MOL007154 | EDNRA | target | tanshinone iia |
| MOL007154 | EDN1 | target | tanshinone iia |
| MOL007154 | CYP3A4 | target | tanshinone iia |
| MOL007154 | CYP1A2 | target | tanshinone iia |
| MOL007154 | MYC | target | tanshinone iia |
| MOL007154 | CYP1A1 | target | tanshinone iia |
| MOL007154 | NR1I2 | target | tanshinone iia |
| MOL007154 | NPM1 | target | tanshinone iia |
| MOL007154 | ECE1 | target | tanshinone iia |
| MOL007154 | CALCR | target | tanshinone iia |
| MOL007154 | ITGB3 | target | tanshinone iia |
| MOL007155 | CHRM1 | target | (6S)-6-(hydroxymethyl)-1,6-dimethyl-8,9-dihydro-7H-naphtho[8,7-g]benzofuran-10,11-dione |
| MOL007155 | SCN5A | target | (6S)-6-(hydroxymethyl)-1,6-dimethyl-8,9-dihydro-7H-naphtho[8,7-g]benzofuran-10,11-dione |
| MOL007155 | PTGS2 | target | (6S)-6-(hydroxymethyl)-1,6-dimethyl-8,9-dihydro-7H-naphtho[8,7-g]benzofuran-10,11-dione |
| MOL007155 | OPRD1 | target | (6S)-6-(hydroxymethyl)-1,6-dimethyl-8,9-dihydro-7H-naphtho[8,7-g]benzofuran-10,11-dione |
| MOL007155 | ACHE | target | (6S)-6-(hydroxymethyl)-1,6-dimethyl-8,9-dihydro-7H-naphtho[8,7-g]benzofuran-10,11-dione |
| MOL007155 | ADRA1A | target | (6S)-6-(hydroxymethyl)-1,6-dimethyl-8,9-dihydro-7H-naphtho[8,7-g]benzofuran-10,11-dione |
| MOL007155 | ADRB2 | target | (6S)-6-(hydroxymethyl)-1,6-dimethyl-8,9-dihydro-7H-naphtho[8,7-g]benzofuran-10,11-dione |
| MOL007155 | OPRM1 | target | (6S)-6-(hydroxymethyl)-1,6-dimethyl-8,9-dihydro-7H-naphtho[8,7-g]benzofuran-10,11-dione |
| MOL007155 | DPP4 | target | (6S)-6-(hydroxymethyl)-1,6-dimethyl-8,9-dihydro-7H-naphtho[8,7-g]benzofuran-10,11-dione |
| MOL007156 | PTGS1 | target | tanshinone Ⅵ |
| MOL007156 | ESR1 | target | tanshinone Ⅵ |
| MOL007156 | AR | target | tanshinone Ⅵ |
| MOL007156 | SCN5A | target | tanshinone Ⅵ |
| MOL007156 | PPARG | target | tanshinone Ⅵ |
| MOL007156 | PTGS2 | target | tanshinone Ⅵ |
| MOL007156 | HSP90AB1 | target | tanshinone Ⅵ |
| MOL001755 | NR3C2 | target | 24-Ethylcholest-4-en-3-one |
| MOL000358 | PTGS1 | target | beta-sitosterol |
| MOL000358 | PTGS2 | target | beta-sitosterol |
| MOL000358 | HSP90AB1 | target | beta-sitosterol |
| MOL000358 | KCNH2 | target | beta-sitosterol |
| MOL000358 | DRD1 | target | beta-sitosterol |
| MOL000358 | CHRM3 | target | beta-sitosterol |
| MOL000358 | CHRM1 | target | beta-sitosterol |
| MOL000358 | SCN5A | target | beta-sitosterol |
| MOL000358 | CHRM4 | target | beta-sitosterol |
| MOL000358 | ADRA1A | target | beta-sitosterol |
| MOL000358 | CHRM2 | target | beta-sitosterol |
| MOL000358 | ADRA1B | target | beta-sitosterol |
| MOL000358 | ADRB2 | target | beta-sitosterol |
| MOL000358 | SLC6A4 | target | beta-sitosterol |
| MOL000358 | OPRM1 | target | beta-sitosterol |
| MOL000358 | BCL2 | target | beta-sitosterol |
| MOL000358 | BAX | target | beta-sitosterol |
| MOL000358 | CASP9 | target | beta-sitosterol |
| MOL000358 | JUN | target | beta-sitosterol |
| MOL000358 | CASP3 | target | beta-sitosterol |
| MOL000358 | CASP8 | target | beta-sitosterol |
| MOL000358 | PRKCA | target | beta-sitosterol |
| MOL000358 | PON1 | target | beta-sitosterol |
| MOL000358 | MAP2 | target | beta-sitosterol |
| MOL000449 | NR3C2 | target | Stigmasterol |
| MOL000449 | ADH1C | target | Stigmasterol |
| MOL000449 | RXRA | target | Stigmasterol |
| MOL000449 | PTGS1 | target | Stigmasterol |
| MOL000449 | PTGS2 | target | Stigmasterol |
| MOL000449 | ADRA2A | target | Stigmasterol |
| MOL000449 | SLC6A2 | target | Stigmasterol |
| MOL000449 | SLC6A3 | target | Stigmasterol |
| MOL000449 | ADRB2 | target | Stigmasterol |
| MOL000449 | AKR1B1 | target | Stigmasterol |
| MOL000449 | PLAU | target | Stigmasterol |
| MOL000449 | MAOB | target | Stigmasterol |
| MOL000449 | MAOA | target | Stigmasterol |
| MOL000449 | CHRM3 | target | Stigmasterol |
| MOL000449 | CHRM1 | target | Stigmasterol |
| MOL000449 | ADRB1 | target | Stigmasterol |
| MOL000449 | SCN5A | target | Stigmasterol |
| MOL000449 | ADRA1A | target | Stigmasterol |
| MOL000449 | CHRM2 | target | Stigmasterol |
| MOL000449 | ADRA1B | target | Stigmasterol |
| MOL000354 | NOS2 | target | isorhamnetin |
| MOL000354 | PTGS1 | target | isorhamnetin |
| MOL000354 | ESR1 | target | isorhamnetin |
| MOL000354 | AR | target | isorhamnetin |
| MOL000354 | PPARG | target | isorhamnetin |
| MOL000354 | PTGS2 | target | isorhamnetin |
| MOL000354 | ESR2 | target | isorhamnetin |
| MOL000354 | DPP4 | target | isorhamnetin |
| MOL000354 | MAPK14 | target | isorhamnetin |
| MOL000354 | GSK3B | target | isorhamnetin |
| MOL000354 | HSP90AB1 | target | isorhamnetin |
| MOL000354 | CDK2 | target | isorhamnetin |
| MOL000354 | PYGM | target | isorhamnetin |
| MOL000354 | PPARD | target | isorhamnetin |
| MOL000354 | CHEK1 | target | isorhamnetin |
| MOL000354 | AKR1B1 | target | isorhamnetin |
| MOL000354 | ACHE | target | isorhamnetin |
| MOL000354 | MAOB | target | isorhamnetin |
| MOL000354 | GRIA2 | target | isorhamnetin |
| MOL000354 | RELA | target | isorhamnetin |
| MOL000354 | NCF1 | target | isorhamnetin |
| MOL000354 | OLR1 | target | isorhamnetin |
| MOL002322 | PTGS2 | target | isovitexin |
| MOL002322 | AR | target | isovitexin |
| MOL002322 | RELA | target | isovitexin |
| MOL002322 | IKBKB | target | isovitexin |
| MOL002322 | TNFAIP6 | target | isovitexin |

| Node | Type | Amomi Fructus | Santali Albi Lignum | Salviae Miltiorrhizae Radix et Rhizoma |  |  |  |  |  |  |  |  |  |  |  |  |  |  |  |  |
| --- | --- | --- | --- | --- | --- | --- | --- | --- | --- | --- | --- | --- | --- | --- | --- | --- | --- | --- | --- | --- |
| MOL007061 | Salviae Miltiorrhizae Radix et Rhizoma | 0 | 0 | 1 |  |  |  |  |  |  |  |  |  |  |  |  |  |  |  |  |
| MOL001755 | Amomi Fructus | 1 | 0 | 0 |  |  |  |  |  |  |  |  |  |  |  |  |  |  |  |  |
| MOL007125 | Salviae Miltiorrhizae Radix et Rhizoma | 0 | 0 | 1 |  |  |  |  |  |  |  |  |  |  |  |  |  |  |  |  |
| MOL002322 | Santali Albi Lignum | 0 | 1 | 0 |  |  |  |  |  |  |  |  |  |  |  |  |  |  |  |  |
| MOL000449 | Amomi Fructus | 1 | 0 | 0 |  |  |  |  |  |  |  |  |  |  |  |  |  |  |  |  |
| MOL000569 | Salviae Miltiorrhizae Radix et Rhizoma | 0 | 0 | 1 |  |  |  |  |  |  |  |  |  |  |  |  |  |  |  |  |
| MOL000006 | multiDrug | 0 | 1 | 1 |  |  |  |  |  |  |  |  |  |  |  |  |  |  |  |  |
| MOL007151 | Salviae Miltiorrhizae Radix et Rhizoma | 0 | 0 | 1 |  |  |  |  |  |  |  |  |  |  |  |  |  |  |  |  |
| MOL007070 | Salviae Miltiorrhizae Radix et Rhizoma | 0 | 0 | 1 |  |  |  |  |  |  |  |  |  |  |  |  |  |  |  |  |
| MOL007150 | Salviae Miltiorrhizae Radix et Rhizoma | 0 | 0 | 1 |  |  |  |  |  |  |  |  |  |  |  |  |  |  |  |  |
| MOL007127 | Salviae Miltiorrhizae Radix et Rhizoma | 0 | 0 | 1 |  |  |  |  |  |  |  |  |  |  |  |  |  |  |  |  |
| MOL007071 | Salviae Miltiorrhizae Radix et Rhizoma | 0 | 0 | 1 |  |  |  |  |  |  |  |  |  |  |  |  |  |  |  |  |
| MOL007045 | Salviae Miltiorrhizae Radix et Rhizoma | 0 | 0 | 1 |  |  |  |  |  |  |  |  |  |  |  |  |  |  |  |  |
| MOL007082 | Salviae Miltiorrhizae Radix et Rhizoma | 0 | 0 | 1 |  |  |  |  |  |  |  |  |  |  |  |  |  |  |  |  |
| MOL002222 | Salviae Miltiorrhizae Radix et Rhizoma | 0 | 0 | 1 |  |  |  |  |  |  |  |  |  |  |  |  |  |  |  |  |
| MOL007048 | Salviae Miltiorrhizae Radix et Rhizoma | 0 | 0 | 1 |  |  |  |  |  |  |  |  |  |  |  |  |  |  |  |  |
| MOL007111 | Salviae Miltiorrhizae Radix et Rhizoma | 0 | 0 | 1 |  |  |  |  |  |  |  |  |  |  |  |  |  |  |  |  |
| MOL007094 | Salviae Miltiorrhizae Radix et Rhizoma | 0 | 0 | 1 |  |  |  |  |  |  |  |  |  |  |  |  |  |  |  |  |
| MOL007063 | Salviae Miltiorrhizae Radix et Rhizoma | 0 | 0 | 1 |  |  |  |  |  |  |  |  |  |  |  |  |  |  |  |  |
| MOL007100 | Salviae Miltiorrhizae Radix et Rhizoma | 0 | 0 | 1 |  |  |  |  |  |  |  |  |  |  |  |  |  |  |  |  |
| MOL001942 | Salviae Miltiorrhizae Radix et Rhizoma | 0 | 0 | 1 |  |  |  |  |  |  |  |  |  |  |  |  |  |  |  |  |
| MOL007101 | Salviae Miltiorrhizae Radix et Rhizoma | 0 | 0 | 1 |  |  |  |  |  |  |  |  |  |  |  |  |  |  |  |  |
| MOL007124 | Salviae Miltiorrhizae Radix et Rhizoma | 0 | 0 | 1 |  |  |  |  |  |  |  |  |  |  |  |  |  |  |  |  |
| MOL007130 | Salviae Miltiorrhizae Radix et Rhizoma | 0 | 0 | 1 |  |  |  |  |  |  |  |  |  |  |  |  |  |  |  |  |
| MOL007050 | Salviae Miltiorrhizae Radix et Rhizoma | 0 | 0 | 1 |  |  |  |  |  |  |  |  |  |  |  |  |  |  |  |  |
| MOL007122 | Salviae Miltiorrhizae Radix et Rhizoma | 0 | 0 | 1 |  |  |  |  |  |  |  |  |  |  |  |  |  |  |  |  |
| MOL002651 | Salviae Miltiorrhizae Radix et Rhizoma | 0 | 0 | 1 |  |  |  |  |  |  |  |  |  |  |  |  |  |  |  |  |
| MOL007145 | Salviae Miltiorrhizae Radix et Rhizoma | 0 | 0 | 1 |  |  |  |  |  |  |  |  |  |  |  |  |  |  |  |  |
| MOL007088 | Salviae Miltiorrhizae Radix et Rhizoma | 0 | 0 | 1 |  |  |  |  |  |  |  |  |  |  |  |  |  |  |  |  |
| MOL007049 | Salviae Miltiorrhizae Radix et Rhizoma | 0 | 0 | 1 |  |  |  |  |  |  |  |  |  |  |  |  |  |  |  |  |
| MOL007098 | Salviae Miltiorrhizae Radix et Rhizoma | 0 | 0 | 1 |  |  |  |  |  |  |  |  |  |  |  |  |  |  |  |  |
| MOL001659 | Salviae Miltiorrhizae Radix et Rhizoma | 0 | 0 | 1 |  |  |  |  |  |  |  |  |  |  |  |  |  |  |  |  |
| MOL007085 | Salviae Miltiorrhizae Radix et Rhizoma | 0 | 0 | 1 |  |  |  |  |  |  |  |  |  |  |  |  |  |  |  |  |
| MOL007141 | Salviae Miltiorrhizae Radix et Rhizoma | 0 | 0 | 1 |  |  |  |  |  |  |  |  |  |  |  |  |  |  |  |  |
| MOL001601 | Salviae Miltiorrhizae Radix et Rhizoma | 0 | 0 | 1 |  |  |  |  |  |  |  |  |  |  |  |  |  |  |  |  |
| MOL007081 | Salviae Miltiorrhizae Radix et Rhizoma | 0 | 0 | 1 |  |  |  |  |  |  |  |  |  |  |  |  |  |  |  |  |
| MOL007119 | Salviae Miltiorrhizae Radix et Rhizoma | 0 | 0 | 1 |  |  |  |  |  |  |  |  |  |  |  |  |  |  |  |  |
| MOL000354 | Santali Albi Lignum | 0 | 1 | 0 |  |  |  |  |  |  |  |  |  |  |  |  |  |  |  |  |
| MOL007154 | Salviae Miltiorrhizae Radix et Rhizoma | 0 | 0 | 1 |  |  |  |  |  |  |  |  |  |  |  |  |  |  |  |  |
| MOL007105 | Salviae Miltiorrhizae Radix et Rhizoma | 0 | 0 | 1 |  |  |  |  |  |  |  |  |  |  |  |  |  |  |  |  |
| MOL007152 | Salviae Miltiorrhizae Radix et Rhizoma | 0 | 0 | 1 |  |  |  |  |  |  |  |  |  |  |  |  |  |  |  |  |
| MOL007156 | Salviae Miltiorrhizae Radix et Rhizoma | 0 | 0 | 1 |  |  |  |  |  |  |  |  |  |  |  |  |  |  |  |  |
| MOL007069 | Salviae Miltiorrhizae Radix et Rhizoma | 0 | 0 | 1 |  |  |  |  |  |  |  |  |  |  |  |  |  |  |  |  |
| MOL007036 | Salviae Miltiorrhizae Radix et Rhizoma | 0 | 0 | 1 |  |  |  |  |  |  |  |  |  |  |  |  |  |  |  |  |
| MOL007058 | Salviae Miltiorrhizae Radix et Rhizoma | 0 | 0 | 1 |  |  |  |  |  |  |  |  |  |  |  |  |  |  |  |  |
| MOL007079 | Salviae Miltiorrhizae Radix et Rhizoma | 0 | 0 | 1 |  |  |  |  |  |  |  |  |  |  |  |  |  |  |  |  |
| MOL007093 | Salviae Miltiorrhizae Radix et Rhizoma | 0 | 0 | 1 |  |  |  |  |  |  |  |  |  |  |  |  |  |  |  |  |
| MOL007107 | Salviae Miltiorrhizae Radix et Rhizoma | 0 | 0 | 1 |  |  |  |  |  |  |  |  |  |  |  |  |  |  |  |  |
| MOL007064 | Salviae Miltiorrhizae Radix et Rhizoma | 0 | 0 | 1 |  |  |  |  |  |  |  |  |  |  |  |  |  |  |  |  |
| MOL007108 | Salviae Miltiorrhizae Radix et Rhizoma | 0 | 0 | 1 |  |  |  |  |  |  |  |  |  |  |  |  |  |  |  |  |
| MOL007143 | Salviae Miltiorrhizae Radix et Rhizoma | 0 | 0 | 1 |  |  |  |  |  |  |  |  |  |  |  |  |  |  |  |  |
| MOL007041 | Salviae Miltiorrhizae Radix et Rhizoma | 0 | 0 | 1 |  |  |  |  |  |  |  |  |  |  |  |  |  |  |  |  |
| MOL007132 | Salviae Miltiorrhizae Radix et Rhizoma | 0 | 0 | 1 |  |  |  |  |  |  |  |  |  |  |  |  |  |  |  |  |
| MOL007077 | Salviae Miltiorrhizae Radix et Rhizoma | 0 | 0 | 1 |  |  |  |  |  |  |  |  |  |  |  |  |  |  |  |  |
| MOL000358 | Amomi Fructus | 1 | 0 | 0 |  |  |  |  |  |  |  |  |  |  |  |  |  |  |  |  |
| MOL007121 | Salviae Miltiorrhizae Radix et Rhizoma | 0 | 0 | 1 |  |  |  |  |  |  |  |  |  |  |  |  |  |  |  |  |
| MOL007120 | Salviae Miltiorrhizae Radix et Rhizoma | 0 | 0 | 1 |  |  |  |  |  |  |  |  |  |  |  |  |  |  |  |  |
| MOL007155 | Salviae Miltiorrhizae Radix et Rhizoma | 0 | 0 | 1 |  |  |  |  |  |  |  |  |  |  |  |  |  |  |  |  |
| MOL007059 | Salviae Miltiorrhizae Radix et Rhizoma | 0 | 0 | 1 |  |  |  |  |  |  |  |  |  |  |  |  |  |  |  |  |
| MOL007068 | Salviae Miltiorrhizae Radix et Rhizoma | 0 | 0 | 1 |  |  |  |  |  |  |  |  |  |  |  |  |  |  |  |  |

| MOL007061 |
| --- |
| MOL001755 |
| MOL007125 |
| MOL002322 |
| MOL000449 |
| MOL000569 |
| MOL000006 |
| MOL007151 |
| MOL007070 |
| MOL007150 |
| MOL007127 |
| MOL007071 |
| MOL007045 |
| MOL007082 |
| MOL002222 |
| MOL007048 |
| MOL007111 |
| MOL007094 |
| MOL007063 |
| MOL007100 |
| MOL001942 |
| MOL007101 |
| MOL007124 |
| MOL007130 |
| MOL007050 |
| MOL007122 |
| MOL002651 |
| MOL007145 |
| MOL007088 |
| MOL007049 |
| MOL007098 |
| MOL001659 |
| MOL007085 |
| MOL007141 |
| MOL001601 |
| MOL007081 |
| MOL007119 |
| MOL000354 |
| MOL007154 |
| MOL007105 |
| MOL007152 |
| MOL007156 |
| MOL007069 |
| MOL007036 |
| MOL007058 |
| MOL007079 |
| MOL007093 |
| MOL007107 |
| MOL007064 |
| MOL007108 |
| MOL007143 |
| MOL007041 |
| MOL007132 |
| MOL007077 |
| MOL000358 |
| MOL007121 |
| MOL007120 |
| MOL007155 |
| MOL007059 |
| MOL007068 |

| PTGS1 |
| --- |
| CHRM3 |
| CHRM1 |
| SCN5A |
| CHRM5 |
| PTGS2 |
| HTR3A |
| CA2 |
| CHRM4 |
| RXRA |
| OPRD1 |
| ADRA1A |
| CHRM2 |
| ADRA1B |
| SLC6A3 |
| ADRB2 |
| ADRA1D |
| OPRM1 |
| HSP90AB1 |
| DRD1 |
| SLC6A4 |
| NR3C2 |
| ACHE |
| DRD2 |
| ESR1 |
| AR |
| PPARG |
| DPP4 |
| AKR1B1 |
| RELA |
| EGFR |
| AKT1 |
| VEGFA |
| CCND1 |
| BCL2L1 |
| CDKN1A |
| CASP9 |
| MMP2 |
| MMP9 |
| MAPK1 |
| RB1 |
| TNFAIP6 |
| JUN |
| IL6R |
| CASP3 |
| TP53 |
| NFKBIA |
| MDM2 |
| APP |
| MMP1 |
| PCNA |
| ERBB2 |
| HMOX1 |
| CASP7 |
| ICAM1 |
| MCL1 |
| BIRC5 |
| IL2RA |
| TYR |
| IFNG |
| IL4 |
| GSTP1 |
| XIAP |
| SLC2A4 |
| CD40LG |
| MET |
| CDK2 |
| ADRA2A |
| ADRA2C |
| NOS2 |
| ESR2 |
| MAPK14 |
| GSK3B |
| NR3C1 |
| KCNH2 |
| STAT3 |
| EDN1 |
| CHEK1 |
| DRD5 |
| SLC6A2 |
| ADRA2B |
| BCL2 |
| FOS |
| EDNRA |
| CYP3A4 |
| CYP1A2 |
| MYC |
| CYP1A1 |
| NR1I2 |
| NPM1 |
| ECE1 |
| CALCR |
| ITGB3 |
| BAX |
| CASP8 |
| PRKCA |
| PON1 |
| MAP2 |
| ADH1C |
| PLAU |
| MAOB |
| MAOA |
| ADRB1 |
| PYGM |
| PPARD |
| GRIA2 |
| NCF1 |
| OLR1 |
| IKBKB |

| **Table S4.** Core gene target score (1) | | | | | | | |
| --- | --- | --- | --- | --- | --- | --- | --- |
| **Shared name** | **Betweenness** | **Closeness** | **Degree** | **Eigenvector** | **LAC** | **name** | **Network** |
| ADRA1A | 0 | 0.207423581 | 8 | 0.035928573 | 7 | ADRA1A | 8 |
| CHRM3 | 0 | 0.207423581 | 8 | 0.035928406 | 7 | CHRM3 | 8 |
| APP | 822.6831028 | 0.240506329 | 20 | 0.098475233 | 6 | APP | 14.40789474 |
| CHRM1 | 112.5867222 | 0.224056604 | 10 | 0.055572696 | 6.2 | CHRM1 | 7.916666667 |
| ADRA1B | 21.42857143 | 0.209713024 | 9 | 0.037951276 | 6.666666667 | ADRA1B | 8 |
| EDNRA | 124.6068356 | 0.230024213 | 10 | 0.064363807 | 6 | EDNRA | 7.652777778 |
| EDN1 | 942.1728042 | 0.245478036 | 17 | 0.122177474 | 4.941176471 | EDN1 | 9.430769231 |
| ADRA1D | 0 | 0.207423581 | 8 | 0.03592848 | 7 | ADRA1D | 8 |
| CHRM5 | 0 | 0.207423581 | 8 | 0.03592848 | 7 | CHRM5 | 8 |
| ADRB2 | 724.1364472 | 0.216400911 | 9 | 0.029426498 | 2.444444444 | ADRB2 | 5.25 |
| ADRA2A | 33.81536797 | 0.211581292 | 9 | 0.02913251 | 6.666666667 | ADRA2A | 7.625 |
| OPRD1 | 121.2994345 | 0.223529412 | 9 | 0.040993538 | 6.444444444 | OPRD1 | 7.375 |
| ADRA2B | 33.81536797 | 0.211581292 | 9 | 0.029132495 | 6.666666667 | ADRA2B | 7.625 |
| ADRA2C | 33.81536797 | 0.211581292 | 9 | 0.029132495 | 6.666666667 | ADRA2C | 7.625 |
| CHRM2 | 140.7267339 | 0.214932127 | 10 | 0.035812248 | 5.8 | CHRM2 | 7.263888889 |
| CHRM4 | 0 | 0.205627706 | 8 | 0.024421005 | 7 | CHRM4 | 8 |
| DRD2 | 184 | 0.206073753 | 9 | 0.024553079 | 6.222222222 | DRD2 | 7.125 |
| PRKCA | 514.2611063 | 0.232273839 | 11 | 0.069654353 | 2 | PRKCA | 2.594444444 |
| OPRM1 | 121.2994345 | 0.223529412 | 9 | 0.040993538 | 6.444444444 | OPRM1 | 7.375 |
| ADRB1 | 0 | 0.179924242 | 4 | 0.002807112 | 3 | ADRB1 | 4 |
| DRD5 | 0 | 0.179924242 | 4 | 0.002807112 | 3 | DRD5 | 4 |
| CALCR | 0 | 0.179924242 | 4 | 0.002807112 | 3 | CALCR | 4 |
| DRD1 | 0 | 0.179924242 | 4 | 0.002807112 | 3 | DRD1 | 4 |
| EGFR | 852.5333995 | 0.24611399 | 14 | 0.138880715 | 3.428571429 | EGFR | 4.414180264 |
| AKT1 | 1116.753932 | 0.25606469 | 29 | 0.283643961 | 6.413793103 | AKT1 | 16.3152381 |
| MAPK1 | 1053.881033 | 0.259562842 | 26 | 0.265765905 | 6.153846154 | MAPK1 | 13.6152077 |
| CCND1 | 96.91158783 | 0.238693467 | 15 | 0.198007643 | 6.933333333 | CCND1 | 9.273823399 |
| MAPK14 | 403.3078306 | 0.246753247 | 19 | 0.212323129 | 5.789473684 | MAPK14 | 10.0079712 |
| MDM2 | 15.00007215 | 0.215419501 | 7 | 0.08444614 | 4.285714286 | MDM2 | 5.666666667 |
| STAT3 | 1382.018152 | 0.254010695 | 28 | 0.246387109 | 5.285714286 | STAT3 | 16.81008177 |
| RB1 | 50.5633353 | 0.230024213 | 13 | 0.164456874 | 6 | RB1 | 8.00959596 |
| TP53 | 938.542906 | 0.25 | 31 | 0.305089325 | 7.741935484 | TP53 | 23.39863988 |
| BCL2L1 | 72.91002374 | 0.230024213 | 11 | 0.122567967 | 5.454545455 | BCL2L1 | 7.866666667 |
| CASP3 | 94.63828632 | 0.225118483 | 12 | 0.118529692 | 4.833333333 | CASP3 | 7.5 |
| SLC2A4 | 1.29038961 | 0.213963964 | 3 | 0.034584161 | 1.333333333 | SLC2A4 | 2 |
| GSK3B | 2.320601414 | 0.219399538 | 7 | 0.111091621 | 4.571428571 | GSK3B | 5.333333333 |
| NOS2 | 49.37758802 | 0.230024213 | 8 | 0.105391443 | 3.75 | NOS2 | 4.476190476 |
| CASP9 | 11.96349206 | 0.213483146 | 6 | 0.051834423 | 4 | CASP9 | 5.2 |
| CD40LG | 7.272343139 | 0.219399538 | 5 | 0.061661113 | 2.4 | CD40LG | 3 |
| XIAP | 17.94141414 | 0.215419501 | 7 | 0.055727009 | 4 | XIAP | 5.4 |
| JUN | 547.4498419 | 0.248691099 | 23 | 0.26205501 | 7.47826087 | JUN | 14.37519425 |
| HSP90AB1 | 49.20115089 | 0.219399538 | 8 | 0.089474127 | 3 | HSP90AB1 | 4.523809524 |
| AR | 67.98699105 | 0.225118483 | 10 | 0.118915357 | 4 | AR | 5.53968254 |
| IL2RA | 8.102863287 | 0.227272727 | 5 | 0.078521289 | 2.8 | IL2RA | 3.5 |
| BCL2 | 107.9014384 | 0.233415233 | 11 | 0.142850742 | 5.454545455 | BCL2 | 7.1 |
| CDKN1A | 58.13642848 | 0.231143552 | 12 | 0.156266674 | 6 | CDKN1A | 7.617099567 |
| ESR1 | 103.4561505 | 0.235148515 | 15 | 0.192099437 | 6.266666667 | ESR1 | 8.608091908 |
| RXRA | 986.4884802 | 0.232273839 | 13 | 0.108079299 | 2.461538462 | RXRA | 3.963924964 |
| IKBKB | 25.34881105 | 0.220417633 | 6 | 0.057851546 | 2 | IKBKB | 2.4 |
| ITGB3 | 90.05556111 | 0.220930233 | 5 | 0.057513703 | 1.2 | ITGB3 | 1.5 |
| VEGFA | 209.0249743 | 0.242966752 | 12 | 0.119063973 | 3 | VEGFA | 5.189393939 |
| MYC | 121.7148914 | 0.238693467 | 17 | 0.227736413 | 8 | MYC | 10.7161963 |
| RELA | 322.5020267 | 0.23929471 | 17 | 0.167522177 | 4.705882353 | RELA | 8.354677267 |
| CASP8 | 295.3679817 | 0.2375 | 13 | 0.11451517 | 3.538461538 | CASP8 | 6.082323232 |
| NR3C1 | 147.9645873 | 0.230582524 | 12 | 0.147133902 | 4.833333333 | NR3C1 | 6.626984127 |
| NR3C2 | 0 | 0.193089431 | 3 | 0.025589341 | 2 | NR3C2 | 3 |
| BAX | 0 | 0.206521739 | 4 | 0.046157897 | 3 | BAX | 4 |
| MCL1 | 11.2459818 | 0.217391304 | 6 | 0.070685975 | 3.666666667 | MCL1 | 4.8 |
| BIRC5 | 0 | 0.211581292 | 2 | 0.03972882 | 1 | BIRC5 | 2 |
| CASP7 | 0 | 0.197916667 | 4 | 0.024534676 | 3 | CASP7 | 4 |
| PCNA | 0.971428571 | 0.204741379 | 6 | 0.080985121 | 4 | PCNA | 4.8 |
| FOS | 105.7722885 | 0.2375 | 14 | 0.184295669 | 6.714285714 | FOS | 8.60955711 |
| CDK2 | 29.38422039 | 0.219907407 | 9 | 0.10933245 | 4.666666667 | CDK2 | 6.466666667 |
| NFKBIA | 46.20358436 | 0.227272727 | 10 | 0.1108955 | 4.4 | NFKBIA | 5.471428571 |
| IL4 | 473.2994987 | 0.226190476 | 9 | 0.081071369 | 2.888888889 | IL4 | 4.916666667 |
| CHEK1 | 0 | 0.20212766 | 3 | 0.03589588 | 2 | CHEK1 | 3 |
| CYP1A1 | 309.6080446 | 0.193877551 | 3 | 0.007943505 | 0 | CYP1A1 | 0 |
| CYP3A4 | 230.6142438 | 0.172101449 | 5 | 0.001620502 | 0.4 | CYP3A4 | 1.5 |
| GSTP1 | 0 | 0.163230241 | 1 | 5.72E-04 | 0 | GSTP1 | 0 |
| CYP1A2 | 0 | 0.147515528 | 1 | 1.17E-04 | 0 | CYP1A2 | 0 |
| NR1I2 | 125.6080446 | 0.193089431 | 2 | 0.007902394 | 0 | NR1I2 | 0 |
| MAOB | 0 | 0.163230241 | 2 | 5.49E-04 | 1 | MAOB | 2 |
| MAOA | 282.0033552 | 0.190763052 | 3 | 0.005998557 | 0.666666667 | MAOA | 1.5 |
| SLC6A3 | 0 | 0.171790235 | 1 | 0.001852798 | 0 | SLC6A3 | 0 |
| ECE1 | 0 | 0.198329854 | 1 | 0.008916256 | 0 | ECE1 | 0 |
| MMP2 | 17.88925261 | 0.216400911 | 5 | 0.039896637 | 2.4 | MMP2 | 3.5 |
| MMP9 | 201.8892526 | 0.216894977 | 6 | 0.040093623 | 2 | MMP9 | 3.2 |
| ERBB2 | 12.22948163 | 0.224586288 | 6 | 0.075267248 | 3 | ERBB2 | 3.9 |
| PLAU | 187 | 0.203426124 | 3 | 0.014271532 | 0 | PLAU | 0 |
| IL6R | 1.082163188 | 0.217391304 | 3 | 0.04236722 | 1.333333333 | IL6R | 2 |
| ESR2 | 0.733846154 | 0.213004484 | 4 | 0.05272134 | 2.5 | ESR2 | 3.333333333 |
| HMOX1 | 2.025174825 | 0.208333333 | 4 | 0.053306039 | 2.5 | HMOX1 | 3.333333333 |
| PPARG | 42.3454758 | 0.224056604 | 7 | 0.088323765 | 2.285714286 | PPARG | 3 |
| GRIA2 | 0 | 0.189620758 | 1 | 0.005054284 | 0 | GRIA2 | 0 |
| ICAM1 | 0 | 0.203862661 | 1 | 0.017767465 | 0 | ICAM1 | 0 |
| IFNG | 0 | 0.209713024 | 3 | 0.036821447 | 2 | IFNG | 3 |
| KCNH2 | 0 | 0.010526316 | 1 | 0 | 0 | KCNH2 | 0 |
| SCN5A | 0 | 0.010526316 | 1 | 0 | 0 | SCN5A | 0 |
| MET | 0.361111111 | 0.217391304 | 3 | 0.045544792 | 1.333333333 | MET | 2 |
| NCF1 | 3.30530925 | 0.209713024 | 2 | 0.02035146 | 0 | NCF1 | 0 |
| MMP1 | 0 | 0.208333333 | 3 | 0.023566954 | 2 | MMP1 | 3 |
| TNFAIP6 | 0 | 0.179245283 | 1 | 0.002906934 | 0 | TNFAIP6 | 0 |
| PTGS2 | 1.852380952 | 0.219399538 | 4 | 0.055925041 | 2 | PTGS2 | 2.666666667 |
| NPM1 | 0 | 0.201271186 | 1 | 0.02196135 | 0 | NPM1 | 0 |
| OLR1 | 0 | 0.169946333 | 1 | 0.001032854 | 0 | OLR1 | 0 |
| PPARD | 0 | 0.189620758 | 1 | 0.007785862 | 0 | PPARD | 0 |

| **Table S4.** Core gene target score (2) | | | | | | | |
| --- | --- | --- | --- | --- | --- | --- | --- |
| **Shared name** | **Betweenness** | **Closeness** | **Degree** | **Eigenvector** | **LAC** | **name** | **Network** |
| NFKBIA | 0.166666667 | 0.545454545 | 5 | 0.114070423 | 3.6 | NFKBIA | 4.5 |
| CDK2 | 0.860714286 | 0.545454545 | 6 | 0.133268639 | 4 | CDK2 | 4.8 |
| CASP3 | 4.244047619 | 0.571428571 | 7 | 0.133673996 | 3.714285714 | CASP3 | 4.333333333 |
| BCL2L1 | 2.133333333 | 0.571428571 | 7 | 0.137423083 | 4.571428571 | BCL2L1 | 5.333333333 |
| TP53 | 41.38419913 | 0.8 | 18 | 0.326362342 | 8.444444444 | TP53 | 16.33479298 |
| NR3C1 | 3.035714286 | 0.585365854 | 8 | 0.169549271 | 5 | NR3C1 | 5.761904762 |
| RB1 | 7.78531746 | 0.631578947 | 10 | 0.200827345 | 5.6 | RB1 | 7.105555556 |
| FOS | 2.676984127 | 0.615384615 | 10 | 0.21874246 | 7 | FOS | 7.968253968 |
| STAT3 | 7.726659452 | 0.648648649 | 11 | 0.235313788 | 6.727272727 | STAT3 | 8.072619048 |
| CASP8 | 14.52626263 | 0.6 | 8 | 0.126950353 | 3 | CASP8 | 3.70952381 |
| MAPK14 | 29.45952381 | 0.705882353 | 14 | 0.256224513 | 6 | MAPK14 | 9.085775336 |
| RELA | 15.63690476 | 0.631578947 | 10 | 0.180627331 | 4.6 | RELA | 6.034920635 |
| CCND1 | 12.5781746 | 0.666666667 | 12 | 0.237668902 | 6.333333333 | CCND1 | 8.269949495 |
| MAPK1 | 62.81172439 | 0.774193548 | 17 | 0.287898958 | 6.352941176 | MAPK1 | 12.04224664 |
| AKT1 | 56.35934343 | 0.75 | 16 | 0.28678593 | 7.125 | AKT1 | 11.8773643 |
| EDN1 | 10.93109668 | 0.585365854 | 7 | 0.113300711 | 3.142857143 | EDN1 | 4.766666667 |
| EDNRA | 2.28459596 | 0.5 | 4 | 0.045443702 | 2 | EDNRA | 2.666666667 |
| MYC | 18.4540404 | 0.705882353 | 14 | 0.276754856 | 7.714285714 | MYC | 11.19614275 |
| CHRM1 | 1.801190476 | 0.489795918 | 4 | 0.046155877 | 2 | CHRM1 | 2.666666667 |
| APP | 12.58268398 | 0.558139535 | 6 | 0.070417047 | 2.333333333 | APP | 3.333333333 |
| ESR1 | 4.46038961 | 0.631578947 | 10 | 0.221763059 | 6.6 | ESR1 | 7.619047619 |
| CDKN1A | 4.791269841 | 0.6 | 9 | 0.186838359 | 5.777777778 | CDKN1A | 6.925 |
| BCL2 | 3.122222222 | 0.585365854 | 8 | 0.166930854 | 5.25 | BCL2 | 6.119047619 |
| AR | 3.933333333 | 0.571428571 | 7 | 0.141012311 | 3.428571429 | AR | 4 |
| JUN | 52.2536075 | 0.75 | 16 | 0.295343995 | 7.625 | JUN | 12.38429903 |

| **Table S4.** Core gene target score (3) | | | | | | | |
| --- | --- | --- | --- | --- | --- | --- | --- |
| **Shared name** | **Betweenness** | **Closeness** | **Degree** | **Eigenvector** | **LAC** | **name** | **Network** |
| MYC | 1.2 | 0.875 | 6 | 0.347482681 | 4 | MYC | 5.25 |
| TP53 | 1.533333333 | 1 | 7 | 0.396472156 | 4.857142857 | TP53 | 7 |
| RB1 | 1.2 | 0.875 | 6 | 0.34748286 | 4 | RB1 | 5.25 |
| MAPK14 | 0.333333333 | 0.777777778 | 5 | 0.309268594 | 3.6 | MAPK14 | 4.5 |
| CCND1 | 0.333333333 | 0.777777778 | 5 | 0.309268564 | 3.6 | CCND1 | 4.5 |
| MAPK1 | 0.333333333 | 0.777777778 | 5 | 0.309268564 | 3.6 | MAPK1 | 4.5 |
| JUN | 1.533333333 | 1 | 7 | 0.396472126 | 4.857142857 | JUN | 7 |
| AKT1 | 1.533333333 | 1 | 7 | 0.396472126 | 4.857142857 | AKT1 | 7 |


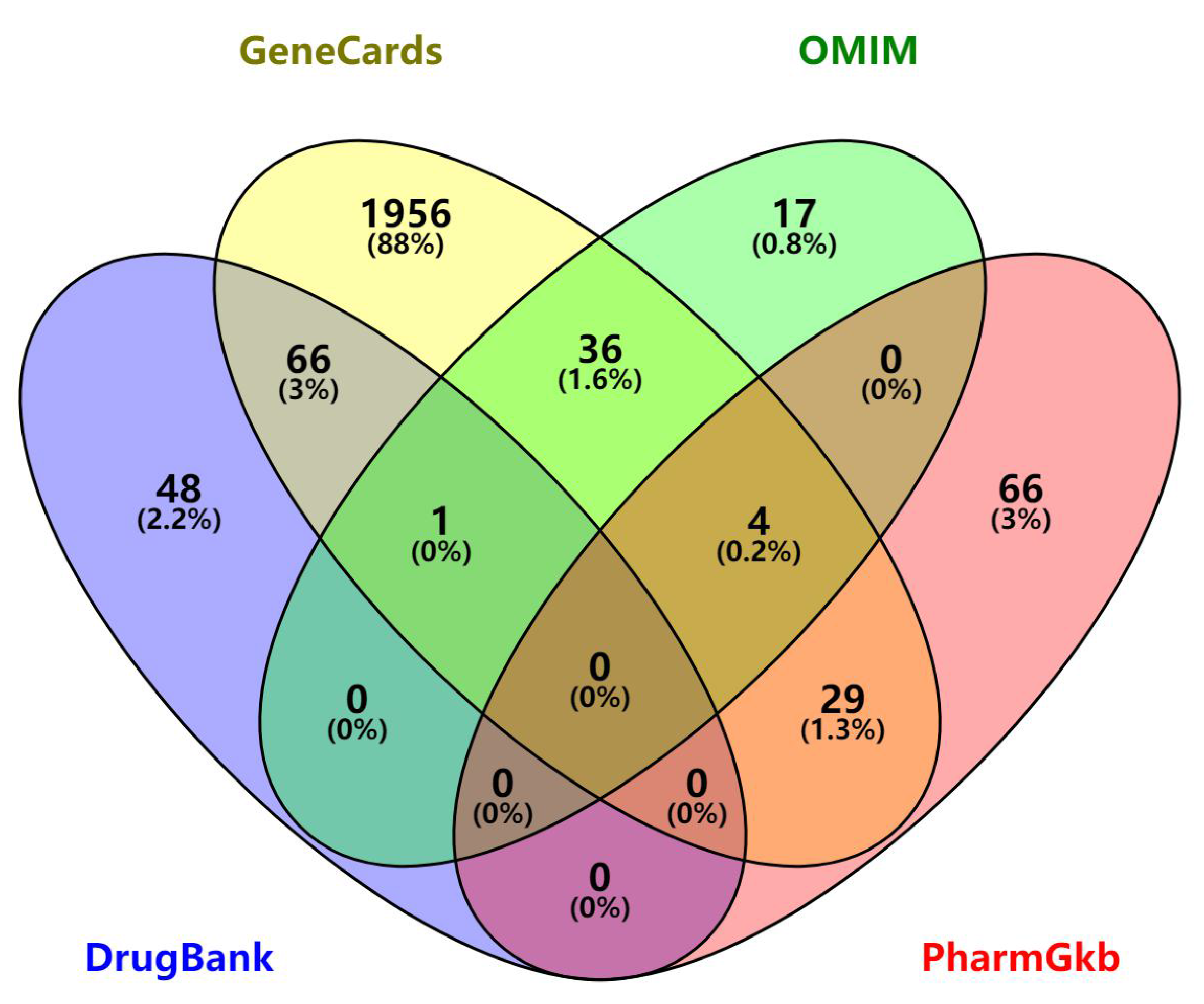
Figure S1

Figure S2


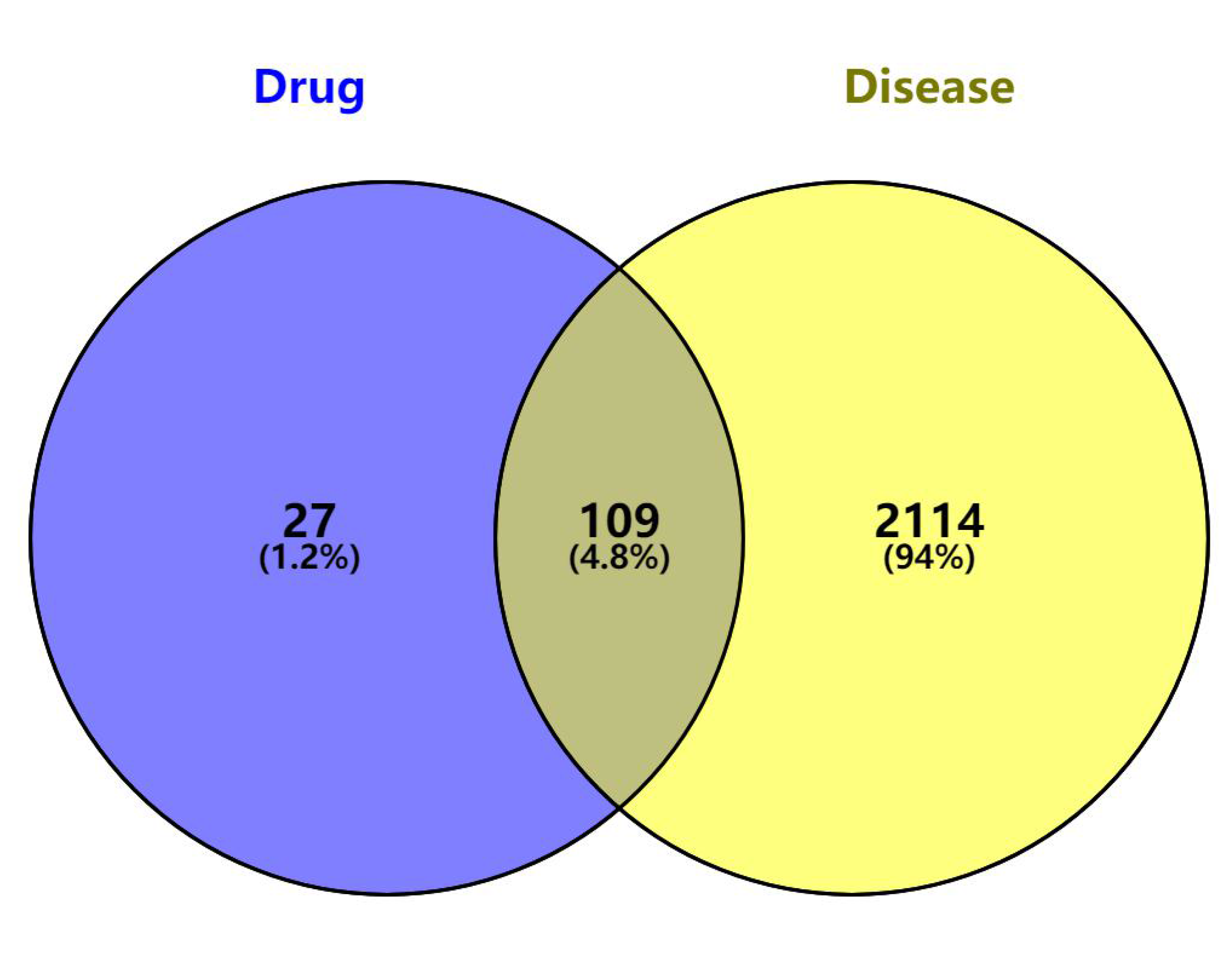


Figure S

3
